# Microsatellite markers: what they mean and why they are so useful

**DOI:** 10.1590/1678-4685-GMB-2016-0027

**Published:** 2016-08-04

**Authors:** Maria Lucia Carneiro Vieira, Luciane Santini, Augusto Lima Diniz, Carla de Freitas Munhoz

**Affiliations:** 1 Departamento de Genética, Escola Superior de Agricultura "Luiz de Queiroz" (ESALQ), Universidade de São Paulo (USP), Piracicaba, SP, Brazil

**Keywords:** SSR biological function, genomic distribution, genotyping approaches, molecular marker, practical utility

## Abstract

Microsatellites or Single Sequence Repeats (SSRs) are extensively employed in plant genetics studies, using both low and high throughput genotyping approaches. Motivated by the importance of these sequences over the last decades this review aims to address some theoretical aspects of SSRs, including definition, characterization and biological function. The methodologies for the development of SSR loci, genotyping and their applications as molecular markers are also reviewed. Finally, two data surveys are presented. The first was conducted using the main database of Web of Science, prospecting for articles published over the period from 2010 to 2015, resulting in approximately 930 records. The second survey was focused on papers that aimed at SSR marker development, published in the American Journal of Botany's Primer Notes and Protocols in Plant Sciences (over 2013 up to 2015), resulting in a total of 87 publications. This scenario confirms the current relevance of SSRs and indicates their continuous utilization in plant science.

## Brief introduction

Ongoing technological advances in all fields of knowledge mean that we cannot be sure which technologies will survive the impact of innovation, and for how long. Over the years, advances in molecular genetics methodology have lead to widespread use of codominant molecular markers, especially Simple Sequence Repeats (SSRs) and, more recently, Single Nucleotide Polymorphisms (SNPs). This paper attempts to present an overview of how the concept of SSRs has evolved and how their biological functions were discovered. We also address the development of methods for identifying polymorphic SSRs, and the application of these markers in genetic analysis. It reveals that much remains to be explored regarding these sequences, particularly in relation to cultivated and wild plants.

## Definition and genome occurrence of microsatellites and their use as genetic markers

Microsatellites (1 to 10 nucleotides) and minisatellites (> 10 nucleotides) are subcategories of tandem repeats (TRs) that, together with the predominant interspersed repeats (or remnants of transposable elements), make up genomic repetitive regions. TRs are evolutionarily relevant due to their instability. They mutate at rates between 10^3^ and 10^6^ per cell generation i.e., up to 10 orders of magnitude greater than point mutations (Gemayel *et al*., 2012).

Microsatellites, Simple Sequence Repeats (SSR), Short Tandem Repeats (STR) and Simple Sequence Length Polymorphisms (SSLP) are found in prokaryotes and eukaryotes. They are widely distributed throughout the genome, especially in the euchromatin of eukaryotes, and coding and non-coding nuclear and organellar DNA (Pérez-Jiménez *et al*., 2013; Phumichai *et al*., 2015).

There is a lot of evidence to back up the hypothesis that SSRs are not randomly distributed along the genome. In a comparative study, SSR distribution was found to be highly non-random and to vary a great deal in different regions of the genes of *Arabidopsis thaliana* and rice (Lawson and Zhang, 2006). In the major cereals, for instance, authors have tended to categorize microsatellites based on different criteria. In barley and *Avena* species, SSRs were classified in two types: those with unique sequences on either flank and those intimately associated with retrotransposons and other dispersed repetitive elements. The second type was found to be less polymorphic in oat cultivars (Ramsay *et al*., 1999; Li *et al*., 2000). Using publicly available DNA sequence information on the rice genome, Temnykh *et al*. (2001) categorized microsatellites based on length and noticed that longer perfect repeats (≥ 20 nucleotides) were highly polymorphic. Microsatellites with SSRs shorter than 12 bp were found to have a mutation potential no different from that of most unique sequences. Moreover, authors reported that ~80% of GC-rich trinucleotides occurred in exons, whereas AT-rich trinucleotides were distributed roughly evenly throughout all genomic components (coding sequences, untranslated regions, introns and intergenic spaces). Tetranucleotide SSRs were predominantly situated in non-coding, mainly intergenic regions of the rice genome. It was later established that the SSR distributions in different regions of the maize genome were non-random, and that density was highest in untranslated regions (UTR), gradually falling off in the promotor, intron, intergenic, and coding sequence regions, in that order (Qu and Liu, 2013).

On the other hand, comparisons of microsatellite distributions in *Rumex acetosa* and *Silene latifolia* chromosomes showed that some motifs (e.g. CAA or TAA) are strongly accumulated in non-recombining regions of the sex chromosome (Y) in both plant species (Kejnovsky *et al*., 2009). Similarly, a very large accumulation consisting mainly of microsatellites on the heterochromatic W chromosome was reported in a group of fish species (*Leporinus* spp.) that share a ZW sex system, showing an interconnection between heterochromatinization and the accumulation of repetitive sequences, which has been proposed as the basis of sex chromosome evolution (Poltronieri *et al.*, 2014).

Generally speaking, it can be affirmed that the occurrence of SSRs is lower in gene regions, due to the fact that SSRs have a high mutation rate that could compromise gene expression. Studies indicate that in coding regions there is a predominance of SSRs with gene motifs of the tri- and hexanucleotide type, the result of selection pressure against mutations that alter the reading frame (Zhang *et al*., 2004; Xu *et al*., 2013b). In humans, the consensus is that SSRs can also originate in coding regions, leading to the appearance of repetitive patterns in protein sequences. In protein sequence database studies, it was reported that tandem repeats are common in many proteins, and the mechanisms involved in their genesis may contribute to the rapid evolution of proteins (Katti *et al*., 2000; Huntley and Golding, 2000).

Repeat polymorphisms usually result from the addition or deletion of the entire repeat units or motifs. Therefore, different individuals exhibit variations as differences in repeat numbers. In other words, the polymorphisms observed in SSRs are the result of differences in the number of repeats of the motif caused by polymerase strand-slippage in DNA replication or by recombination errors. Strand-slippage replication is a DNA replication error in which the template and nascent strands are mismatched. This means that the template strand can loop out, causing contraction. The nascent strand can also loop out, leading to repeat expansion. Recombination events, such as unequal crossing over and gene conversion, may additionally lead to SSR sequence contractions and expansions. According to several authors, the longer and purer the repeat, the higher the mutation frequency, whereas shorter repeats with lower purity have a lower mutation frequency.

Mutations that have evaded correction by the DNA mismatch repair system form new alleles at SSR loci. For this reason, different alleles may exist at a given SSR locus, which means that SSRs are more informative than other molecular markers, including SNPs.

As for their composition, SSRs can be classified according to motif as: *i)* perfect if composed entirely of repeats of a single motif; *ii)* imperfect if a base pair not belonging to the motif occurs between repeats; *iii)* interrupted if a sequence of a few base pairs is inserted into the motif; or *iv)* composite if formed by multiple, adjacent, repetitive motifs (reviewed in Oliveira *et al*., 2006; revisited by Mason, 2015).

SSRs have been the most widely used markers for genotyping plants over the past 20 years because they are highly informative, codominant, multi-allele genetic markers that are experimentally reproducible and transferable among related species (Mason, 2015). In particular, SSRs are useful for wild species (*i*) in studies of diversity measured on the basis of genetic distance; (*ii*) to estimate gene flow and crossing over rates; and (*iii*) in evolutionary studies, above all to infer infraspecific genetic relations. On the other hand, for cultivated plants SSRs are commonly used for (*i*) constructing linkage maps; (*ii*) mapping loci involved in quantitative traits (QTL); (*iii*) estimating the degree of kinship between genotypes; (*iv*) using marker-assisted selection; and (*v*) defining cultivar DNA fingerprints (Jonah *et al*., 2011; Kalia *et al*., 2011). SSRs have been particularly useful for generating integrated maps for plant species in which full-sib families are used for constructing linkage maps (Garcia *et al*., 2006; Souza *et al*., 2013; Pereira *et al*., 2013), and for combining genetic, physical, and sequence-based maps (Temnykh, 2001), providing breeders and geneticists with a tool to link phenotypic and genotypic variation (see Mammadov *et al*., 2012; Hayward *et al*., 2015 for review articles).

These markers are enormously useful in studies of population structure, genetic mapping, and evolutionary processes. SSRs with core repeats 3 to 5 nucleotides long are preferred in forensics and parentage analysis. It is worth noting that a number of SSR search algorithms have been developed, including TRF (Benson, 1999), SSRIT (Temnykh, 2001), MISA (Thiel *et al*., 2003), SSRFinder (Gao *et al*., 2003), TROLL (Castelo *et al*., 2002) and SciRoKo (Kofler *et al*., 2007).

## Detailing the biological functions of SSRs

Despite the wide applicability of SSRs as genetic markers since their discovery in the 1980s, little is known about the biological importance of microsatellites (Tautz and Renz, 1984), especially in plants. Morgante *et al*. (2002) estimated the density of SSRs in *Arabidopsis thaliana*, rice (*Oryza sativa*), soybean (*Glycine max*), maize (*Zea mays*) and wheat (*Triticum aestivum*) and observed a high frequency of SSRs in transcribed regions, especially in untranslated regions (UTRs). Interestingly, there are substantial data indicating that SSR expansions or contractions in protein-coding regions can lead to a gain or loss of gene function via frameshift mutation or expanded toxic mRNAs. SSR variations in 5'-UTRs could regulate gene expression by affecting transcription and translation, but expansions in the 3'-UTRs cause transcription slippage and produce expanded mRNA, which can disrupt splicing and may disrupt other cellular functions. Intronic SSRs can affect gene transcription, mRNA splicing, or export to cytoplasm. Triplet SSRs located in UTRs or introns can also induce heterochromatin-mediated-like gene silencing. All these effects can eventually lead to phenotypic changes (Li *et al*., 2004; Nalavade *et al*., 2013).

In fact, variation in the length of DNA triplet repeats has been linked to phenotypic variability in microbes and to several human disorders, including Huntington's disease which is caused mainly by (CAG)_n_ expansions. Moreover, the frequencies of different codon repeats vary considerably depending on the type of encoded amino acid. In plants, a triplet repeat-associated genetic defect was identified in a wild variety of *A. thaliana* that carries a dramatically expanded TTC/GAA repeat in the intron of the gene encoding the large subunit 1 of the isopropyl malate isomerase. Expansion of the repeat causes an environment-dependent reduction in the enzyme's activity and severely impairs plant growth, whereas contraction of the expanded repeat can reverse the detrimental effect on the phenotype (Sureshkumar *et al*., 2009).

Historically, tandem repeats have been designated as nonfunctional DNA, mainly because they are highly unstable. With the exception of tandem repeats involved in human neurodegenerative diseases, repeat variation was often believed to be neutral with no phenotypic consequences (see Gemayel *et al*., 2012).

The detection of microsatellites in transcripts and regulatory regions of the genome encouraged scientific interest in discovering their possible biological functions. More and more publications have presented evidence that microsatellites play a role in relevant processes, such as the regulation of transcription and translation, organization of chromatin, genome size and the cell cycle (Nevo, 2001; Li *et al*., 2004; Gao *et al*., 2013).

As mentioned above, most of the knowledge acquired on microsatellites occurring in genes was obtained by studying humans and animals, indicating their relationship with the manifestation of disease. In bacteria, maintaining numerous microsatellite variants provides a source of highly mutable sequences that enable prompt generation of novel variations, ensuring the survival of the bacterial population in widely varying environments, and adaptation to pathogenesis and virulence. Nevertheless, few studies have focused on whether the typical instability of microsatellites is linked to phenotypic effects in plants (Li *et al*., 2004; Gao *et al*., 2013). However, thanks to whole genome sequencing the important role repeats might play in genomes is being elucidated.

The consensus is that the biological function of a microsatellite is related to its position in the genome. For instance, SSRs in 5'-UTRs serve as protein binding sites, thereby regulating gene translation and protein component and function, as classically demonstrated for the human genes for thymidylate synthase (Horie *et al*., 1995) and calmodulin-1 (Toutenhoofd *et al*., 1998). Ten years later, SSR densities in different regions (5'-UTRs, introns, coding exons, 3'-UTRs, and upstream regions) in housekeeping and tissue-specific genes in human and mouse were compared. Specifically, SSRs in the 5'-UTRs of housekeeping genes are more abundant than in tissue-specific genes. Additionally, it was suggested that SSRs may have an effect on gene expression and may play an important role in contributing to the different expression profiles of housekeeping and tissue-specific genes (Lawson and Zhang, 2008).

In plants, despite the fact that a high density of SSRs has been detected in 5'-UTR regions (Fujimori *et al*., 2003; Tranbarger *et al*., 2012; Zhao *et al*., 2014), there are few studies verifying their effect on the regulation of gene expression. Additionally, tri- and hexanucleotide coding repeats appear to be controlled by stronger mutation pressure in coding regions than in other gene regions. Consequently, in plants there is less allele variability in exonic SSRs than in intronic SSRs. The biased distribution of microsatellites and microsatellite motifs also suggests that microsatellites of different types play different roles in different gene regions, such as within promoters, introns and exons in plants (Li *et al*., 2004; Gemayel *et al*., 2012; Gao *et al*., 2013).

Comparison among SSRs located in CDS, 5' UTR and 3' UTR in the transcriptome of *Sargassum thunbergii*, an economically important brown macroalgae has confirmed that UTR regions harbored more microsatellite compared to the CDS, and the length variation of microsatellite was significantly affected by repeat motif size. Remarkably were the results relative to the function of microsatellite-containing transcripts. After an enrichment analysis, four pathways, i.e. ubiquitin-mediated proteolysis, RNA degradation, spliceosome and terpenoid backbone biosynthesis were obtained, providing new insights into the function and evolution of microsatellite in transcript sequences (Liu *et al*., 2016).

Microsatellites located in introns can play a role in the transport and alternative splicing of mRNA and in gene silencing, as well as in the regulation of transcription, acting independently or in combination with SSRs present in 5'-UTR regions (Kalia *et al*., 2011). A number of examples of the effects of intronic SSRs in humans were reviewed by Li *et al*. (2004), including an increase in the expression of the type I collagen alpha2 gene, caused by the presence of (CA)_n_ repeats in the 5'-UTR region and (GT)_n_ repeats in the first intron.

The 3'-UTR region is also subject to alterations due to the presence of SSRs which cause slippage during the transcription or modification of target regions whose translation is controlled by miRNAs (Li *et al*., 2004; Gao *et al*., 2013). An example of the effect of polymerase slippage in 3'-UTR regions is the multisystem disorder myotonic dystrophy type 1, caused by expansion of a CTG trinucleotide repeat. Normal alleles have 5 to 34 CTG repeats, but alleles with > 50 CTG repeats are associated with disease manifestations (see Ranum and Day, 2002; Li *et al*., 2004; Bird, 2015).

Finally, microsatellites are known to affect expression if present in gene promoters and intergenic regions. In the promotor, SSRs render gene expression vulnerable to possible alterations caused by expansion or contraction of repeat sequences. These alterations result in an increase or reduction in the level of gene expression caused by changes in transcription factor linkage sites and can even culminate in gene silencing. Tandem repeats in intergenic regions can cause changes in the secondary structure of the DNA by forming loops and altering the chromatin, which indirectly results in alterations in the expression of nearby genes (Gao *et al*., 2013).

**Figure 1:**
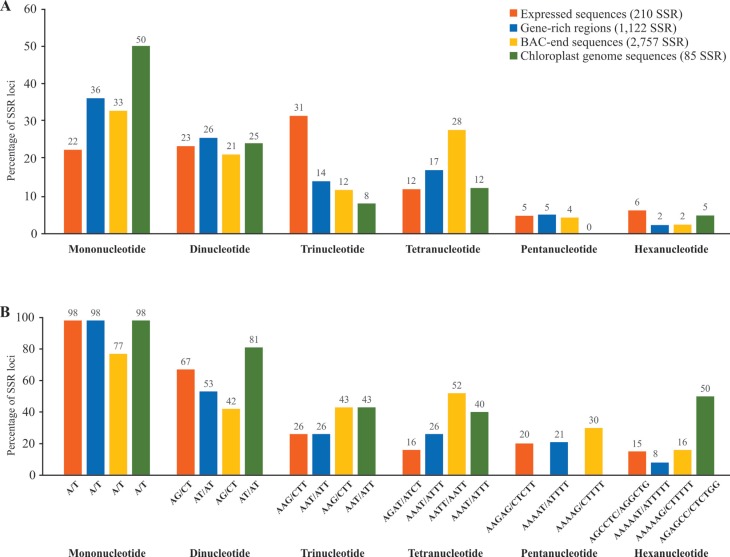
The percentage of mono-, di-, tri-, tetra-, penta- and hexanucleotides in the microsatellites found in expressed sequences, gene-rich regions, BAC-end sequences and in the chloroplast genome of *Passiflora edulis* (Passifloraceae) (A); the percentage of the most common motif is displayed for each case (B).

In spite of the scarcity of studies on the functional changes brought about by SSRs in plants, their effects are believed to be similar to those found in humans. For instance, the occurrence of trinucleotide repeats in *Arabidopsis* genome was found to be twice as frequent in coding regions, suggesting selection for certain stretches of amino acids (Morgante *et al*., 2002). Using data generated in our laboratory, we have compared the percentage of SSRs having mono-, di-, tri, tetra-, penta and hexanucleotide motifs in expressed sequences, gene-rich regions, BAC-end sequences and chloroplast genome sequences of *Passiflora edulis*, and identified the prevalent motif in each case. We also noticed the prevalence of tri- and hexanucleotide motifs in expressed sequences (Figure 1).

Recently, based on the genomes available in the Phytozome database, Zhao *et al*. (2014) analyzed the distribution of tandem repeats in 29 species of terrestrial plants and two species of algae, in which the density of repeat sequences was higher in introns and coding sequences; in plants, 5'-UTR and upstream intergenic 200 nucleotide regions had the first and second highest densities.

In cDNA libraries constructed using plant and reproductive tissues of *Elaeis guineensis*, SSRs were observed in both coding regions and UTRs (Tranbarger *et al*., 2012). The majority were identified in open reading frames, indicating a possible effect on the gene product and consequently on gene function. On the other hand, mutations in SSRs located in UTRs could affect transcription, translation or transcript splicing (Tranbarger *et al*., 2012).

An important example of the functioning of SSRs in plants was reported by Liu *et al*. (2014b) using a high-throughput sequencing approach to characterize miRNAs and their targeted transcripts in different tissues of sweet orange. These miRNAs were evenly distributed across the genome in several small clusters, and 69 pre-miRNAs were co-localized with SSRs. Noticeably, the loop size of a particular pre-miRNA was influenced by the repeat number of the CUU codon. Another important aspect is the instability of microsatellites. Studies conducted on transgenic plants of *A. thaliana* showed that this instability increases as the plant ages, mainly due to a drop in the efficiency of DNA repair mechanisms (Golubov *et al*., 2010). This peculiarity means that SSR markers can be used to assess the impacts of mutagenic contaminants. Mutagenesis induced in *Pisum sativum* by high doses of lead was detected based on the instability of microsatellites at a locus involved in metabolizing glutamine (Rodriguez *et al*., 2013).

Microsatellite alterations associated with diseases in humans are widely known and can give the false impression that the effects of these mutations are predominantly adverse. On the contrary, some examples provide evidence that SSR alleles can offer potential selective advantages (Kashi and King, 2006). It was therefore time to abandon the presumption that SSRs are junk DNA. SSRs are currently qualified as relevant to population adaptation and phenotypic plasticity within and across generations and gene-associated tandem repeats act as evolutionary facilitators, providing abundant, robust variation and thus enabling rapid development of new forms (Nevo, 2001; Kashi and King, 2006).

## Development of SSR markers, including *de novo* nucleotide sequences for finding SSRs

**Figure 2:**
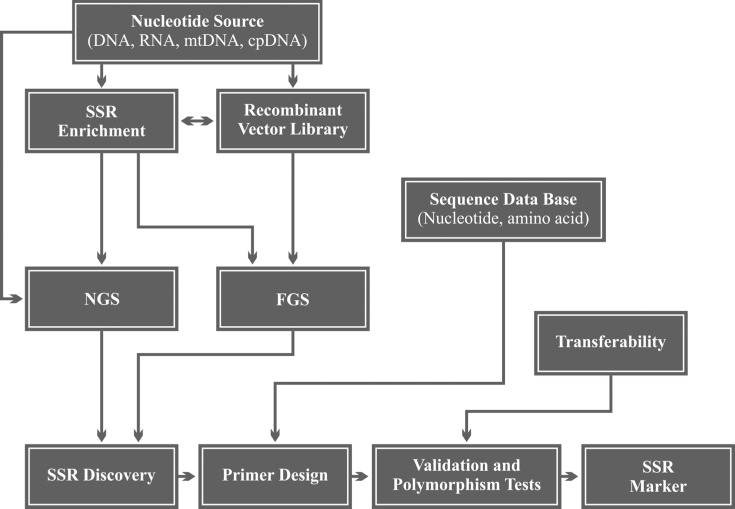
Workflow steps of SSR marker development.

The development of SSR markers can basically be divided into the following stages: (*i*) prior knowledge of nucleotide sequences in which SSRs occur; (*ii*) design of oligonucleotides (or primers) complementary to the regions flanking the SSR; (*iii*) validation of primers by PCR and electrophoresis of the product of the reaction, and (*iv*) detection of polymorphisms among individuals (Mason, 2015). A schematic workflow showing how an SSR marker can be obtained is given in Figure 2. Interestingly, the efficiency of SSR marker development was found to be associated with the microsatellite class. In rice, for instance, the rate of successful amplification varied from 31.7% (AT repeats) up to 87% (GAA repeats). The following figures were observed for other SSR classes: GA, 83.8%; CA, 71.8%; GC-rich trinucleotides, 64.45%; ATT, 78,3%; CAT and CAA, 83,3% and tetranucleotides, 71.4% (Temnykh *et al*., 2001).

Microsatellites were originally developed from both coding and non-coding regions of plant genomes, and several sources were used to search for SSRs, including a variety of DNA libraries (genomic, genomic-enriched for SSR, bacterial artificial chromosome and cDNA libraries), as well as public databases, including expressed sequence tag (EST) databases (see Hanai *et al*., 2007).

In prospecting for SSRs, the first step consists of constructing enriched genomic libraries and various enrichment methods have been successfully developed (Billotte *et al*., 1999; Maio and Castro, 2013). To construct and sequence genomic libraries, the DNA is fragmented, ligated to adaptors and inserted into vectors for transforming *Escherichia coli*. Most protocols involve a stage of enrichment for repetitive sequences that can be achieved using selective hybridization, PCR or both techniques (Senan *et al*., 2014). In enrichment by hybridization, positive clones are detected using radioactively or chemically labeled SSR probes. Finally, these clones are selected by PCR amplification and sequencing (Semagn *et al*., 2006; Blair *et al*., 2009). Another way of enriching a library is to use biotinized SSR probes that are captured by streptavidin-coated beads (Nunome *et al*., 2006). The captured DNA is eluted, amplified, cloned and sequenced. The enriched libraries are screened to identify clones containing SSRs, producing the subsample of repetitive sequences that is intrinsic to this approach. PCR-based methods can bias the sampling of repetitive sequences in non-enriched libraries, since fragment selection and amplification are dependent on complementarity with specific primers for the SSR and cloning vector. However, non-enriched libraries and alternative methods derived from other molecular markers (e.g. RAPD and AFLP) have also been used to find SSRs (see Senan *et al*., 2014).

The advances made in Next Generation Sequencing (NGS) have provided a new scenario for detecting microsatellites. Various NGS-based projects have been developed over the last few decades, generating an enormous quantity of sequences made available in public databases and widely used for prospecting for microsatellites. Automation of the original sequencing method proposed by Sanger and Coulson (1975) has made it possible to sequence the complete genome of *A. thaliana* (Arabidopsis Genome Initiative, 2000). However, because of the high cost of the Sanger method when sequencing complete genomes, it has been replaced by NGS platforms or a combination of both methods (Schnable *et al*., 2009).

NGS has been very useful for various studies, including prospecting for new SSR markers. Successors of the Sanger sequencing method include the 454 FLX (Roche), Solexa (Illumina), SOLiD (Applied Biosystems) and HeliScope True Single Molecule Sequencing (Helicos) platforms. Third generation platforms are also currently available, including a platform developed by Pacific Biosciences (PacBio), based on a new sequencing technology, SMRT sequencing, which has the advantage of producing longer DNA reads.

Each platform has specific characteristics in terms of the number and size of reads generated, run time, as well as the accuracy and cost of each base read, with both advantages and disadvantages compared to other platforms (Egan *et al*., 2012). In order to advice researchers in sequencing technology choice, Alic *et al*. (2016) published a review about different high-throughput sequencing methods and 50 stand-alone softwares used to control errors. Control error analysis is one of the most important steps in sequencing data analysis, mainly in *de novo* sequencing projects, that lack a reference genome. Furthermore, sequences that contain repetitive regions are challenges to be overcome by error correction methods, due to their vulnerability to errors. Initiatives for sequencing the complete genomes of various species use combinations of different platforms with the aim of incorporating the best features of each and extracting the maximum amount of information.

Currently, 454 and Illumina are the NGS platforms most widely used for developing SSR markers. However, the PacBio SMRT sequencing technology is being considered an economically viable alternative for discovering microsatellites (Grohme *et al*., 2013).

## *In-silico* prospecting and transferability of SSR markers

With the advent of NGS, it was necessary to create databases for storing the information generated. In addition to genomic sequences, a large quantity of expressed sequence tags (EST) derived from cDNA libraries (i.e. originating from mRNA) were also generated. The online database platforms for nucleotide, protein and transcript data available for the majority of plant species are relatively small when compared to model species, such as *A. thaliana*, *Glycine max, O. sativa* and *Z. mays*. Since the protocols for obtaining and isolating *de novo* SSR loci can be expensive and not viable in some cases, the investigation of these elements *in silico* (i.e. in the actual databases) is a promising strategy. This approach is possible only because SSR loci primers are transferable among different, phylogenetically matching species (Kuleung *et al*., 2004).

The possibility of interchanging this genetic information is ascribed to the synteny between matching species. Although there are some exceptions resulting from structural rearrangements, synteny is an import attribute of plant genomes and is inversely proportional to the phylogenetic distance between species (Kaló *et al*., 2004). The conservation of this information could indicate that these loci confer evolutionary advantages, and are therefore subject to low selection pressure (Zhu, 2005).

Microsatellites found in the chloroplast genome of higher plants (cpSSRs) consist basically of mononucleotide repeats (A and T) (Bryan *et al*., 1999). Contrarily, we have found 50, 25, 8, 12 and 5% of mono-, di-, tri-, tetra- and hexanucleotides respectively in the microsatellites of the chloroplast genome of *Passiflora edulis* (Figure 1A), but we have confirmed that mononucleotide repeats consisted predominantly of A/T repeats (98%, Figure 1B). In terms of transferability, cpSSRs are particularly promising for the study of phylogenetically distant species, since the regions flanking them are strongly conserved, so that universal primers can be developed (Weising and Gardner, 1999; Ebert and Peakall, 2009).

## Genotyping

After identifying the sequences containing SSRs, specific primers must be synthesized (18 and 25 bp in length), complementary to the flanking regions, followed by amplification and polymorphism testing. According to Guichoux *et al*. (2011), a number of experimental problems can arise during SSR amplification, which can compromise allele calling and binning, resulting in increased error rates or the need for extensive manual corrections. These authors itemized possible solutions for aiding researchers to solve these problems, such as stuttering or shadow bands, non-template addition of a nucleotide by the *Taq* polymerase, primer mispriming, etc.

Once the SSR markers have been produced, genotyping can begin. It is a relatively easy and low-cost procedure. The allele variants of a given SSR locus can be identified by agarose gel electrophoresis (AGE) or polyacrylamide gel electrophoresis (PAGE), low-complexity methods used routinely in molecular genetics laboratories. PAGE genotyping is more labor intensive but provides better resolution, allowing identification of given polymorphisms for a single base pair (Penha *et al*., 2013; Mason, 2015). Alternatively, marked SSR primers can be synthesized with fluorescent markers for genotyping by capillary electrophoresis using conventional sequencers (Araújo *et al*., 2007; Csencsics *et al*., 2010; Agarwal *et al*., 2015). In this case, each DNA sample is loaded into a capillary containing a polyacrylamide matrix in which the electrophoresis is performed. The fluorescence emitted by the marked primer is captured and the molecular mass of the amplified fragment is determined. The result is an electropherogram showing luminescence peaks corresponding to each amplified allele. Lastly, the genotyping stage consists of comparing the electropherograms of different individuals (see Culley *et al*., 2013; Mason, 2015), a technique that is particularly widely used when working with complex genome species, such as sugarcane and other polyploids (Morais TBR de, 2012, Doctoral Thesis. Escola Superior de Agricultura "Luiz de Queiroz, University of São Paulo, Piracicaba, SP, Brazil).

The most appropriate genotyping method for each project is defined according to the species under investigation, the sensitivity required in determining allele variations, the availability of the equipment and cost effectiveness. The amplification and genotyping stages can be perfected to multiplex different SSR loci, cutting costs and saving time, and allowing large scale analysis (Brown *et al*., 1996; Guichoux *et al*., 2011; Lepais and Bacles, 2011). There are two ways of performing multiplexed analysis of microsatellite loci. The first is by multiplexed PCR, in which different SSR primers are placed in the same reaction tube. The following stages are essential: *i)* determining the length (in bp) of the alleles at each SSR locus; *ii)* selecting loci whose allele lengths are not superimposed; *iii)* *in silico* testing at melting temperature (T_m_) and the possible formation of secondary structures between the primers of the SSR loci selected. The second multiplexed SSR loci analysis method entails multiplexed genotyping. In this case, amplifications are performed separately, but the amplified products of a biological sample are mixed and loaded into the same electrophoresis gel channel or sequencing capillary.

Guichoux *et al*. (2011) have published an outstanding analysis of current trends in microsatellite genotyping. Several aspects are reviewed, including the overall cost of SSR genotyping as a function of the degree of multiplexing and the number of genotyped samples. For instance, the most widely cited commercial kit has a cost per sample of 1.88. The authors then suggest solutions to cut the final cost per sample. According to these authors, most of the work done to develop and optimize SSR multiplexing actually consists of phases common to all SSR development projects.

In the past, alternative methods have been developed to facilitate genotyped PCR multiplexing by capillary electrophoresis, such as the M13 tailed primer method (Oetting *et al*., 1995). In this method, the sequencing reaction is performed as a multiplexed PCR using the M13 (reverse) primer, conjugated with a fluorescent colorant and various modified SSR (forward) primers. The SSR primers are modified by a 19-bp extension at the 5' end, identical to the M13 nucleotide sequence. In the first PCR cycle, amplification is based on the SSR primers, forming an M13 annealing site at the 3' end, used in the second amplification cycle. A variant of this technique (Multiplex-Ready PCR) was subsequently published with the aim of cutting the cost of primer marking, which is usually 5 to 10 times that of conventional primer synthesis (Hayden *et al*., 2008).

## Current overview

**Figure 3:**
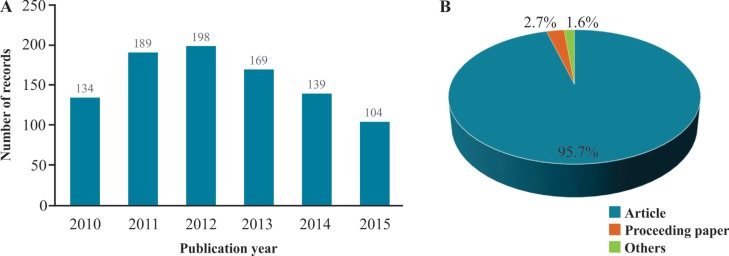
Number of publications relating to the use of microsatellites in crop genetic studies from 2010 to 2015 according to the Web of Science database (A). Distribution of records according to the type of publication (B).

Microsatellite genomic distribution, biological function and practical utility have been reviewed in a number of articles over the past two decades, some of which are highlighted here: Jarne and Lagoda (1996); Schlötterer (1998); Li *et al*. (2002); Buschiazzo and Gemmell (2006); Oliveira *et al*. (2006); Sun *et al*. (2009); Guichoux *et al*. (2011); Gemayel *et al*. (2012); Senan *et al*. (2014); Mason (2015). With the aim of investigating the use of microsatellite markers over the period from 2010 to 2015 in the genetic analysis of cultivated plants, we conducted a search in the main database of Web of Science (Web of Science^TM^ Core Collection). We entered "microsatellite" or "SSR marker" in the title field and "crop*" in the topic field. To avoid selecting records related to plant pathogens and insect pests, the following terms were excluded from the topic field: bacteria (bacter*), fungi (fung*), insect (insect*) and pathogen (pathogen*). Finally, the search was refined by selecting the field of Plant Science, and all resulting hits were manually checked. We found 933 unique records (Figure 3, Supplementary Material Table S1) showing that microsatellites continue to be used as high-relevance molecular markers in the genetic analysis of cultivated plants. The number of publications rose steadily until 2012, and then fell back, possibly due to the ease with which genetic studies could be carried out using SNPs.

Recent studies have shown that the easiest way of identifying SSR loci is by using NGS to sequence the genome or transcriptome. Zalapa *et al*. (2012) reviewed papers published in the American Journal of Botany's Primer Notes and Protocols in Plant Sciences, an important monthly journal that centralizes a significant number of publications related to the discovery and use of SSRs in plants. Note that the use of the Sanger method was predominant up to this time, as well as the use of genomic libraries enriched with sequences containing microsatellites. Since then, there is a tendency to replace this method by NGS genome or transcriptome sequencing.

With the aim of comparing this scenario with the current situation, we conducted a similar survey based on papers published in the AJB from January 2013 to December 2015, selecting only those papers in which sequences were generated by developing SSR markers. A total of 87 papers were published during this period, the majority of which involved using the Sanger method to sequence genomic libraries enriched with SSRs. It is worth noting that the use of NGS for prospecting for and generating SSR markers has been on the increase, surpassing the Sanger method in 2015 (Table 1). We also realized that the enrichment stage might no longer be advantageous, due to the number of sequences generated by NGS. On the contrary, since the composition of the nucleotide base and the frequency of SSR motifs can actually vary among plant genomes (Li *et al*., 2002), the enrichment stage with a small number of motifs should allow curtailment or subsampling of the population of SSRs to be discovered.

**Table 1:** Recent studies involved in the detection and development of SSR markers in plants, using different sequencing technologies.

Technology	Source^1^	Library^2^	Enrichment	Species	Reference
Sanger	G	Y	CA repeats and (GA)_10_	*Aniba rosaeodora*	Angrizani *et al*., 2013
	T	**N**	Expressed sequence tags from roots	*Callerya speciosa*	Li *et al*., 2013
	G	Y	(AG)_10_, (GT)_15_, (CAG)_8_ and (AC)_6_(AG)_5_	*Canavalia cathartica* and *C. lineata*	Yamashiro *et al*., 2013
	G	Y	(CT)_8_ and (GT)_8_	*Cariniana legalis*	Tambarussi *et al*., 2013
	G	Y	CA, AAC, ATG, and TAGA	*Ceanothus megacarpus*	Ishibashi *et al*., 2013
	G	Y	(GT)_12_	*Cornus sanguinea*	Wadl *et al*., 2013
	G	Y	(AC)_15_, (AG)_15_, and (AAG)_10_	*Diplarche multiflora*	Zhang *et al*., 2013
	G	Y	(CT)_8_ and (GT)_8_	*Encholirium horridum*	Hmeljevski *et al*., 2013
	G	Y	Not informed	*Lagerstroemia indica*	Liu *et al*., 2013c
	G	Y	(AC)_6_(AG)_7_ or (AC)_6_(TC)_7_	*Leptospermum recurvum*	Ando *et al*., 2013
	G	Y	(AC)_6_(AG)_5_ or (TC)_6_(AC)_5_	*Lilium longiflorum*	Sakazono *et al*., 2013
	G	Y	(AG)_15_ and (AC)_15_	*Melastoma dodecandrum*	Liu *et al*., 2013b
	G	Y	TC_n_, TG_n_, and GATA_n_	*Miscanthus* ssp. and *Saccharum* ssp.	Hodkinson *et al*., 2013
	G	Y	(AC)_18_/(AG)_18_/(ATG)_12_	*Myriophyllum spicatum*	Wu *et al*., 2013
	G	Y	(GT)_15_ and (AG)_15_	*Phellodendron amurense*	Yu *et al*., 2013
	G	Y	(CAT)_11_, (GCA)_6_, (GATA)_11_, (AAC)_12_, (ATTT)_10_(GC)_8_, (GCGA)_5_, (TTC)_15_ and (GGT)_7_	*Pinus edulis* and *P. monophylla*	Krohn *et al*., 2013
	G	Y	(AC)_n_	*Pinus wangii*	Dou *et al*., 2013
	T	**N**	Expressed sequence tags	*Pisum sativum*	Jain and McPhee 2013
	G	Y	(GA)_12_ and (CA)_12_	*Prunus sibirica*	Liu *et al*., 2013a
	G	Y	(AG)_13_ and (TC)_13_	*Rhizophora mangle*	Ribeiro *et al*., 2013
	G	Y	(CCG)_6_, (AAG)_8_, (AGG)_6_, (CT)_13_, (AGC)_6_, (AC)_10,_ and (ATC)_6_	*Rhodiola ssp*	You *et al*., 2013
	G	Y	(CT)_8_ and (GT)_8_	*Smilax brasiliensis*	Martins *et al*., 2013
	G	Y	(AC)_6_(AG)_5_ or (TC)_6_(AC)_5_	*Tricyrtis macrantha*	Ohki and Setoguchi 2013
	G	Y	(AC)_13_ and (AG)_13_	*Vernicia fordii*	Pan *et al*., 2013
Illumina	T	**N**	Expressed sequence tags from leaves	*Firmiana danxiaensis*	Fan *et al*., 2013
	G	**N**	*	*Gleditsia triacanthos*	Owusu *et al*., 2013
	T	**N**	Expressed sequence tags	*Spartina alterniflora*	Guo *et al*., 2013
454	G	Y	CT and GT	*Anthyllis vulneraria*	Kesselring *et al*., 2013
	G	Y	CT and GT	*Berberis microphylla*	Varas *et al*., 2013
	G	Y	(GT)_8_ (TC)_9.5_, (GTT)_6.33_, (TTC)_7_, (GTA)_8.33_, (GTG)_4.67_, (TCC)_5_, (TTTG)_2.5_, (TTTC)_6_, (TTAC)_6.75_, and (GATG)_4.25_	*Elaeagnus angustifolia*	Gaskin *et al*., 2013
	G	**N**	*	*Melaleuca argentea*	Nevill *et al*., 2013
	G	Y	Not informed	*Pachyrhizus* Rich. ex DC.	Delêtre *et al*., 2013
	G	Y	(GT)_15_, (CT)_15_, (GATA)_10_, (GACA)_8_, and (GATGT)_5_	*Phoradendron californicum*	Arroyo *et al*., 2013
	T	**N**	Expressed sequence tags from stem	*Pisum sativum*	Zhuang *et al*., 2013
	G	**N**	*	*Prosopis alba* and *P. chilensis*	Bessega *et al*., 2013
	G	Y	(CT)_13_ and (GT)_13_	*Sebaea aurea*	Kissling *et al*., 2013
	G	Y	TG,TC, AAC, AAG, AGG, ACG, ACAT and ACTC	*Thuja occidentalis*	Xu *et al*., 2013a
	G	**N**	*	*Virola sebifera*	Wei *et al*., 2013
					
	G	Y	(GA)_15_, (GTA)_8_, and (TTC)_8_	*Argania spinosa*	Bahloul *et al*., 2014
Sanger	G	Y	(CT)_8_, (GT)_8_	*Byrsonima cydoniifolia*	Bernardes *et al*., 2014
	Cp	**N**	*	*Lemna minor*	Wani *et al*., 2014
	G	Y	CT	*Lobelia inflata*	Hughes *et al*., 2014
	G	Y	(GT)_8_ and (CT)_8_	*Passiflora* ssp.	Cerqueira-Silva *et al*., 2014
	G	Y	(CT)_8_ and (GT)_8_	*Piper solmsianum*	Yoshida *et al*., 2014
	G	Y	(AC)_6_(AG)_5_ or (TC)_6_(AC)_5_	*Scrophularia incisa*	Wang *et al*., 2014
	G	Y	(AC)_15_ and (AG)_15_	*Spiraea* ssp	Khan *et al*., 2014
	G	Y	(AC)_6_(AG)_5_, (TC)_6_(AC)_5_	*Vitex rotundifolia*	Ohtsuki *et al*., 2014
	G	Y	(GA)_n_ and (GT)_n_	*Xanthosoma sagittifolium*	Cathebras *et al*., 2014
Illumina	T	**N**	Expressed sequence tags from roots	*Buxus* spp.	Thammina *et al*., 2014
	G	**N**	*	*Macadamia* ssp.	Nock *et al*., 2014
	T	**N**	Expressed sequence tags from leaves	*Ostryopsis* ssp.	Liu *et al*., 2014a
	G	**N**	*In silico* mining	*Phoenix dactylifera*	Aberlenc-Bertossi *et al*., 2014
	G	**N**	*	*Solidago* L.	Beck *et al*., 2014
	G	**N**	*	*Saxifraga granulata*	Meer *et al*., 2014
454	G	Y	(GA)_15_, (GTA)_8_, and (TTC)_8_	*Argania spinosa*	Bahloul *et al*., 2014
	G	**N**	*	*Agave utahensis*	Byers *et al*., 2014
	G	**N**	*	*Bidens alba*	Lu *et al*., 2014
	G	Y	CT and GT	*Nephroma* ssp.	Belinchón *et al*., 2014
	G	Y	TG, TC, AAC, AAG, AGG, ACG, ACAT, and ACTC	*Parietaria judaica*	Bossu *et al*., 2014
Sanger	G	Y	(CT)_8_ and (GT)_8_	*Cabomba aquatica*	Barbosa *et al*., 2015
	G	Y	(AT)_8_, (GA)_8_, and (GAA)_8_	*Calibrachoa heterophylla*	Silva-Arias *et al*., 2015
	G	Y	GA, GT, AGA, ACT, and ATC	*Campanula pyramidalis*	Radosavljeviæ *et al*., 2015
	G	Y	(AC)_15_ and (AG)_15_	*Commelina communis*	Li *et al*., 2015
	G	Y	(AG)_10_	*Ilex chinensis*	Chen *et al*., 2015
	G	Y	Not informed	*Fothergilla intermedia*	Hatmaker *et al*., 2015
	G	Y	(AC)_6_(AG)_5_ or (GA)_5_(CA)_5_	*Hepatica nobilis var. japonica*	Kameoka *et al*., 2015
	G	Y	(CT)_8_ and (GT)_8_	*Philcoxia minensis*	Scatigna *et al*., 2015
	G	Y	(AG)_17_, (AC)_17_, (AAC)_10_, (CCG)_10_, (CTG)_10_, and (AAT)_10_	*Psittacanthus schiedeanus*	González *et al*., 2015
	G	Y	(AG)_17_, (AC)_17_, (AAC)_10_, (CCG)_10_, (CTG)_10_, and (AAT)_10_	*Quillaja saponaria*	Letelier *et al*., 2015
	G	Y	(AC)_15_ and (AG)_15_	*Saxifraga egregia*	Zhang *et al*., 2015
	G	Y	(TTC)_10_, (CG)_10_, and (GT)_10_	*Vellozia squamata*	Duarte-Barbosa *et al*., 2015
Illumina	T/Cp	**N**	Expressed sequence tags from leaves	*Artocarpus moraceae*	Gardner *et al*., 2015
	T	**N**	Expressed sequence tags from leaves	*Bombax ceiba*	Ju *et al*., 2015
	T	**N**	Expressed sequence tags from leaves	*Carallia brachiata*	Qiang *et al*., 2015
	G	**N**	*	*Dendrobium calamiforme*	Trapnell *et al*., 2015
	T	**N**	Expressed sequence tags from leaves	*Lablab purpureus, Lathyrus sativus*	Chapman 2015
				*Psophocarpus tetragonolobus* and *Vigna subterranea*	
	T	**N**	Expressed sequence tags from leaves and cambium	*Thujopsis dolabrata var. hondae*	Sato *et al*., 2015
454	G	**N**	*	*Cyperus fuscus*	Böckelmann *et al*., 2015
	G	**N**	*In silico* mining	*Metasequoia glyptostroboides*	Jin *et al*., 2015
	G	**N**	*	*Pilosella alpicola*	Vít *et al*., 2015
	G	**N**	*	*Pulsatilla vulgaris*	DiLeo *et al*., 2015
	G	Y	(AG)_10_, (AC)_10_, (AAC)_8_, (ACG)_8_, (AAG)_8_, (AGG)_8_, (ACAT)_6_, and (ATCT)_6_	*Quercus variabilis*	Wang *et al*., 2015
	G	Y	CT and GT	*Salix humboldtiana*	Bozzi *et al*., 2015
	G	**N**	*	*Silene acaulis*	Müller *et al*., 2015
	G	Y	TG, TC, AAC, AAG, AGG, ACG, ACAT, and ACTC	*Veronica* subsect*. Pentasepalae*	López-González *et al*., 2015
	G	**N**	*	*Vinca minor*	Moeller *et al*., 2015

1 G = genome, T = transcriptome, Cp = chloroplast DNA

2 SSR enrichment library: Y, yes; **N**, no

* Total genomic DNA sequencing

Another interesting trend is that the Illumina platform is being routinely used for transcriptome sequencing. The advantage of developing SSR markers from transcribed sequences includes the possibility of finding associations with genes and phenotypes (Li *et al*., 2002). As observed by Zalapa *et al*. (2012), a common factor of all the papers, irrespective of the sequencing method, is that only a small fraction of the SSR loci discovered have been assessed. As mentioned earlier, obtaining a sequence is only the first stage in the marker development process. Primer design and PCR optimization still represent a bottleneck. Furthermore, there is always the possibility that the locus is monomorphic, i.e. non-informative.

One strategy for working around this limitation is to track loci polymorphisms *in silico*, during the stage at which regions that contain SSRs are identified. This can be done using two or more genetically contrasting individuals or their progeny (F_1_) for performing NGS, increasing the possibility of sampling alleles based on the alignment of the sequences obtained, and thereby avoiding the synthesis and testing of primers for monomorphic loci (Iorizzo *et al*., 2011).

Finally, visiting the website of the 24^th^ edition of the Plant and Animal Genome (PAG) Conference (San Diego, CA) held in January 2016, we were able to find 63 workshops, abstracts and posters in which the term SSR was employed. We categorized these studies according to the groups of species analyzed and found the great majority of them (~90%) related to plants. We also checked references to SNPs and found about 150 studies, two-thirds related to plants and a third to domesticated animals (cattle, chicken, horse, pig, sheep and fish). A few (1.5% for SSRs and 5% for SNPs) proposed advances in experimental approaches or novel bioinformatics tools (https://pag.confex.com/pag/xxiv/meetingapp.cgi). Are SNPs destined to replace SSRs as the preferred marker? It seems clear that this will occur, but we do believe that SSRs will still be applicable in future plant genetic and genomic studies.

## Supplementary Material

The following online material is available for this article:

Table S1 - List of 933 publications on microsatellite markers (WOS 2010-2015)
