# Supplementary material for: Microsatellite markers: what they mean and why they are so useful
Source: Genet Mol Biol. 2016 Aug 4;39(3):312–28. doi: 10.1590/1678-4685-GMB-2016-0027 (PMC5004837; doi:10.1590/1678-4685-GMB-2016-0027)
Supplement: Supplementary file 1 [file 1415-4757-gmb-1678-4685-GMB-2016-0027-Suppl01.pdf]

Table S1 - List of 933 publications in which microsatellite markers were used in plant genetic analyses, published over the period 2010–2015. Records were found in the Web of Science^{TM} Core Collection

	Author	Publication title	Source	Publication type	ISSN	Publication date	Vol	Issue	Pages	DOI
1	An, D et al.	EST-PCR, EST-SSR and ISSR markers to identify a set of wild cranberries and evaluate their relationships	Canadian Journal of Plant Science	Article	0008-4220	Nov 2015	95	6	1155 1165	10.4141/CJPS-2015-158
2	Chen, WW et al.	Development and characterization of 25 microsatellite primers for *Ilex chinensis* (Aquifoliaceae)	Applications in Plant Sciences	Article	2168-0450	Oct 2015	3	10	NA	10.3732/apps.1500057
3	Jiao, Z et al.	Isolation and characterization of microsatellite loci in *Rehmannia glutinosa* (Scrophulariaceae), a medicinal herb	Applications in Plant Sciences	Article	2168-0450	Oct 2015	3	10	NA	10.3732/apps.1500054
4	Li, Y; Zhang, W	Isolation and characterization of microsatellite markers for *Jasminum sambac* (Oleaceae) using Illumina shotgun sequencing	Applications in Plant Sciences	Article	2168-0450	Oct 2015	3	10	NA	10.3732/apps.1500063
5	Prinz, K; Finkeldey, R	Characterization and transferability of microsatellite markers developed for *Carpinus betulus* (Betulaceae)	Applications in Plant Sciences	Article	2168-0450	Oct 2015	3	10	NA	10.3732/apps.1500053
6	Chen, H et al.	Development of SSR markers and assessment of genetic diversity of adzuki bean in the Chinese germplasm collection	Molecular Breeding	Article	1380-3743	Oct 2015	35	10	NA	10.1007/s11032-015-0383-5
7	Andeden, EE et al.	Development, characterization and mapping of microsatellite markers for lentil (*Lens culinaris* Medik.)	Plant Breeding	Article	0179-9541	Oct 2015	134	5	589 598	10.1111/pbr.12296
8	Thomson, AM et al.	Despite introgressive hybridization, North American birches (*Betula* spp.) maintain strong differentiation at nuclear microsatellite loci	Tree Genetics & Genomes	Article	1614-2942	Oct 2015	11	5	NA	10.1007/s11295-015-0922-6
9	Dixon, GB; DeWald, LE	Microsatellite survey reveals possible link between triploidy and mortality of quaking aspen in Kaibab National Forest, Arizona	Canadian Journal of Forest Research	Article	0045-5067	Oct 2015	45	10	1369 1375	10.1139/cjfr-2014-0566
10	Arriesgado, DM et al.	Isolation and characterization of novel microsatellite markers for *Cymodocea serrulata* (Cymodoceaceae), a seagrass distributed widely in the Indo-Pacific region	Plant Species Biology	Editorial material	0913-557X	Oct 2015	30	4	297 299	10.1111/1442-1984.12064
11	Amagai, Y et al.	Microsatellite mapping of the mutant gene conferring interrupted development of leaf blade in *Triticum aestivum* L.	Genetic Resources & Crop Evolution	Article	0925-9864	Oct 2015	62	7	985 989	10.1007/s10722-015-0302-y
12	Amagai, Y et al.	Microsatellite mapping of the gene for sham ramification in spikelets derived from a hexaploid wheat (*Triticum* spp.) accession171ACS	Genetic Resources & Crop Evolution	Article	0925-9864	Oct 2015	62	7	1079 1084	10.1007/s10722-014-0213-3
13	Rijal, DP et al.	Microsatellite markers for *Heracleum persicum* (Apiaceae) and allied taxa: application of next-generation sequencing to develop genetic resources for invasive species management	Plant Molecular Biology Reporter	Article	0735-9640	Oct 2015	33	5	1381 1390	10.1007/s11105-014-0841-y
14	Phumichai, C et al.	Novel chloroplast microsatellite (cpSSR) markers for genetic diversity assessment of cultivated and wild *Hevea* rubber	Plant Molecular Biology Reporter	Article	0735-9640	Oct 2015	33	5	1486 1498	10.1007/s11105-014-0850-x
15	Gurcan, K et al.	Evaluation of turkish apricot germplasm using SSR markers: Genetic diversity assessment and search for *Plum pox* virus resistance alleles	Scientia Horticulturae	Article	0304-4238	Sep 2015	193		155 164	10.1016/j.scienta.2015.07.012
16	Kumar, M et al.	Molecular breeding in *Brassica* for salt tolerance: importance of microsatellite (SSR) markers for molecular breeding in *Brassica*	Frontiers in Plant Science	Review	1664-462X	Sep 2015	6		NA	10.3389/fpls.2015.00688
17	Gardner, EM et al.	Chloroplast microsatellite markers for *Artocarpus* (Moraceae) developed from transcriptome sequences	Applications in Plant Sciences	Article	2168-0450	Sep 2015	3	9	NA	10.3732/apps.1500049
18	Muller, E et al.	Characterization of 14 microsatellite markers for *Silene acaulis* (Caryophyllaceae)	Applications in Plant Sciences	Article	2168-0450	Sep 2015	3	9	NA	10.3732/apps.1500036
19	Nowell, VJ et al.	Development and characterization of 11 microsatellite primers for the sedge *Trichophorum planifolium* (Cyperaceae)	Applications in Plant Sciences	Article	2168-0450	Sep 2015	3	9	NA	10.3732/apps.1500050
20	Shang, H et al.	Development and characterization of microsatellite loci in the pantropical fern *Hypolepis punctata* (Dennstaedtiaceae)	Applications in Plant Sciences	Article	2168-0450	Sep 2015	3	9	NA	10.3732/apps.1500047
21	Zhang, X et al.	Development of microsatellite loci for the endangered seagrass *Zostera japonica* (Zosteraceae)	Applications in Plant Sciences	Article	2168-0450	Sep 2015	3	9	NA	10.3732/apps.1500064
22	Pratap, A et al.	Genome scanning of Asiatic *Vigna* species for discerning population genetic structure based on microsatellite variation	Molecular Breeding	Article	1380-3743	Sep 2015	35	9	NA	10.1007/s11032-015-0355-9

	Author	Publication title	Source	Publication type	ISSN	Publication date	Vol	Issue	Pages	DOI
23	Onoue, N et al.	Kinship and inbreeding estimates based on microsatellite markers in breeding of Japanese pear (*Pyrus pyrifolia* Nakai)	Euphytica	Article	0014-2336	Sep 2015	205	2	539 555	10.1007/s10681-015-1427-y
24	Bhawna Abdin, MZ et al.	Development of novel gene-based microsatellite markers for robust genotyping purposes in *Lagenaria siceraria*	Scientia Horticulturae	Article	0304-4238	Aug 2015	191	NA	15 24	10.1016/j.scienta.2015.05.006
25	Fuller, RS et al.	Characterization of 13 microsatellite markers for *Calochortus gunnisonii* (Liliaceae) from Illumina MiSeq sequencing	Applications in Plant Sciences	Article	2168-0450	Aug 2015	3	8	NA	10.3732/apps.1500051
26	Merritt, BJ et al.	An empirical review: characteristics of plant microsatellite markers that confer higher levels of genetic variation	Applications in Plant Sciences	Review	2168-0450	Aug 2015	3	8	NA	10.3732/apps.1500025
27	Qin, LF et al.	Development of microsatellite markers in *Ilex kaushue* (Aquifoliaceae), a medicinal plant species	Applications in Plant Sciences	Article	2168-0450	Aug 2015	3	8	NA	10.3732/apps.1500040
28	Sakaguchi, S et al.	Development of nuclear and chloroplast microsatellite markers for the endangered conifer *Callitris sulcata* (Cupressaceae)	Applications in Plant Sciences	Article	2168-0450	Aug 2015	3	8	NA	10.3732/apps.1500045
29	Scatigna, AV et al.	Microsatellite markers for studies with the carnivorous plant *Philcoxia minensis* (Plantaginaceae)	Applications in Plant Sciences	Article	2168-0450	Aug 2015	3	8	NA	10.3732/apps.1500035
30	Vit, P et al.	Microsatellite markers for the *Pilosella alpicola* group (Hieraciinae, Asteraceae) and their cross-amplification in other Hieraciinae genera	Applications in Plant Sciences	Article	2168-0450	Aug 2015	3	8	NA	10.3732/apps.1500048
31	Zhang, FQ et al.	Development and characterization of polymorphic microsatellite loci for *Saxifraga egregia* (Saxifragaceae)	Applications in Plant Sciences	Article	2168-0450	Aug 2015	3	8	NA	10.3732/apps.1500037
32	Zhang, L et al.	Development of microsatellite markers in tung tree (*Vernicia fordii*) using cassava genomic sequences	Plant Molecular Biology Reporter	Article	0735-9640	Aug 2015	33	4	893 904	10.1007/s11105-014-0804-3
33	Wang, D et al.	Comparative transcriptome analyses of drought-resistant and -susceptible *Brassica napus* L. and development of EST-SSR markers by RNA-Seq	Journal of Plant Biology	Article	1226-9239	Aug 2015	58	4	259 269	10.1007/s12374-015-0113-x
34	Mongkolporn, O et al.	Establishment of a core collection of chilli germplasm using microsatellite analysis	Plant Genetic Resources	Article	1479-2621	Aug 2015	13	2	104 110	10.1017/S1479262114000768
35	Zhai, C et al.	Development of *Gossypium anomalum*-derived microsatellite markers and their use for genome-wide identification of recombination between the *G. anomalum* and *G. hirsutum* genomes	Theoretical & Applied Genetics	Article	0040-5752	Aug 2015	128	8	1531 1540	10.1007/s00122-015-2528-7
36	Di Leo, MF et al.	Highly polymorphic microsatellite markers in *Pulsatilla vulgaris* (Ranunculaceae) using next-generation sequencing	Applications in Plant Sciences	Article	2168-0450	Jul 2015	3	7	NA	10.3732/apps.1500031
37	Zheng, LN et al.	Development and characterization of microsatellite loci for *Ficus hirta* (Moracea)	Applications in Plant Sciences	Article	2168-0450	Jul 2015	3	7	NA	10.3732/apps.1500034
38	Sharma, RK et al.	Identification and cross-species amplification of microsatellite markers derived from expressed sequence data of rose species	Journal of Plant Biochemistry & Biotechnology	Article	0971-7811	Jul 2015	24	3	359 364	10.1007/s13562-014-0287-1
39	Abakemal, D et al.	Genetic purity and patterns of relationships among tropical highland adapted quality protein and normal maize inbred lines using microsatellite markers	Euphytica	Article	0014-2336	Jul 2015	204	1	49 61	10.1007/s10681-014-1332-9
40	Li, C et al.	Genetic diversity and structure of American lotus (*Nelumbo lutea* Willd.) in North America revealed from microsatellite markers	Scientia Horticulturae	Article	0304-4238	Jun 2015	189	NA	17 21	10.1016/j.scienta.2015.03.026
41	Ntuli, NR et al.	Genetic diversity in *Cucurbita pepo* landraces revealed by RAPD and SSR markers	Scientia Horticulturae	Article	0304-4238	Jun 2015	189	NA	192 200	10.1016/j.scienta.2015.03.020
42	Klips, RA	DNA microsatellite analysis of sporophytes of the short-lived moss *Physcomitrium pyriforme* reveals a predominantly self-fertilizing mating pattern	Bryologist	Article	0007-2745	Summer 2015	118	2	200 211	10.1639/0007-2745-118.2.200
43	Li, Y et al.	Development and characterization of microsatellite markers for *Veratrum maackii* (Melanthiaceae)	Applications in Plant Sciences	Article	2168-0450	Jun 2015	3	6	NA	10.3732/apps.1500030
44	Prebble, JM et al.	Microsatellite markers for the New Zealand endemic myosotis *Pygmaea* species group (Boraginaceae) amplify across species	Applications in Plant Sciences	Article	2168-0450	Jun 2015	3	6	NA	10.3732/apps.1500027
45	Saeki, I et al.	Development and evaluation of microsatellite markers for *Acer miyabei* (Sapindaceae), a threatened maple species in East Asia	Applications In Plant Sciences	Article	2168-0450	Jun 2015	3	6	NA	10.3732/apps.1500020

	Author	Publication title	Source	Publication type	ISSN	Publication date	Vol	Issue	Pages	DOI
46	Trapnell, DW et al.	Characterization of microsatellite loci for an Australian epiphytic orchid, *Dendrobium calamiforme*, using Illumina sequencing	Applications In Plant Sciences	Article	2168-0450	Jun 2015	3	6	NA	10.3732/apps.1500016
47	Wang, X et al.	Isolation and characterization of microsatellite markers for an endemic tree in East Asia, *Quercus variabilis* (Fagaceae)	Applications In Plant Sciences	Article	2168-0450	Jun 2015	3	6	NA	10.3732/apps.1500032
48	You, YN et al.	Development and characterisation of EST-SSR markers by transcriptome sequencing in taro (*Colocasia esculenta* (L.) Schoot)	Molecular Breeding	Article	1380-3743	Jun 2015	35	6	NA	10.1007/s11032-015-0307-4
49	Hu, J et al.	Microsatellite diversity, population structure, and core collection formation in melon germplasm	Plant Molecular Biology Reporter	Article	0735-9640	Jun 2015	33	3	439 447	10.1007/s11105-014-0757-6
50	Ravishankar, KV et al.	Genetic diversity and population structure analysis of mango (*Mangifera indica*) cultivars assessed by microsatellite markers	Trees	Article	0931-1890	Jun 2015	29	3	775 783	10.1007/s00468-015-1155-x
51	Maurya, R et al.	Genomic-derived microsatellite markers for diversity analysis in *Jatropha curcas*	Trees	Article	0931-1890	Jun 2015	29	3	849 858	10.1007/s00468-015-1166-7
52	Iwaizumi, MG et al.	Highly polymorphic nuclear microsatellite markers reveal detailed patterns of genetic variation in natural populations of Yezo spruce in Hokkaido	Journal of Forest Research	Correction	1341-6979	Jun 2015	20	3	364 364	10.1007/s10310-015-0489-y
53	Tubic, NK et al.	Microsatellite DNA variation within and among invasive populations of *Ambrosia artemisiifolia* from the southern Pannonian Plain	Weed Research	Article	0043-1737	Jun 2015	55	3	268 277	10.1111/wre.12139
54	Durgesh, K et al.	Assessment of genetic diversity based on agro-morphological traits and genic microsatellite markers in inter-specific derivatives and cultivars of pigeonpea	Indian Journal of Genetics & Plant Breeding	Article	0019-5200	May 2015	75	2	215 224	10.5958/0975-6906.2015.00033.4
55	Dossett, M et al.	Development and transferability of black and red raspberry microsatellite markers from short-read sequences	Journal of theAmerican Society for Horticultural Science	Article	0003-1062	May 2015	140	3	243 252	NA
56	Celik, M et al.	Development of microsatellite primers in the protected species *Viola elatior* (Violaceae) using next-generation sequencing	Applications in Plant Sciences	Article	2168-0450	May 2015	3	5	NA	10.3732/apps.1500011
57	Guillemaud, T et al.	Development of 23 polymorphic microsatellite loci in invasive silver wattle, *Acacia dealbata* (Fabaceae)	Applications in Plant Sciences	Article	2168-0450	May 2015	3	5	NA	10.3732/apps.1500018
58	Letelier, L et al.	Isolation and characterization of 12 microsatellite loci in soapbark, *Quillaja saponaria* (Quillajaceae)	Applications in Plant Sciences	Article	2168-0450	May 2015	3	5	NA	10.3732/apps.1500024
59	Shehzad, T; Okuno, K	QTL mapping for yield and yield-contributing traits in sorghum (*Sorghum bicolor* (L.) Moench) with genome-based SSR markers	Euphytica	Article	0014-2336	May 2015	203	1	17 31	10.1007/s10681-014-1243-9
60	Yousaf, Z et al.	Systematic validation of medicinally important genus *Epimedium* species based on microsatellite markers	Pakistan Journal of Botany	Article	0556-3321	Apr 2015	47	2	477 484	NA
61	Sharma, V et al.	Development of SSR and ILP markers in horsegram (*Macrotyloma uniflorum*), their characterization, cross-transferability and relevance for mapping	Molecular Breeding	Article	1380-3743	Apr 2015	35	4	NA	10.1007/s11032-015-0297-2
62	Hossain, F et al.	Mapping and validation of microsatellite markers linked to sugary1 and shrunken2 genes in maize (*Zea mays* L.)	Journal of Plant Biochemistry & Biotechnology	Article	0971-7811	Apr-Jun 2015	24	2	135 142	10.1007/s13562-013-0245-3
63	Forrest, CN et al.	Microsatellite primers for vulnerable and thriving *Acacia* (Fabaceae) species from Australias arid zone	Applications In Plant Sciences	Article	2168-0450	Apr 2015	3	4	NA	10.3732/apps.1400121
64	Ricono, A et al.	Development and characterization of microsatellite loci for the endangered scrub lupine, *Lupinus aridorum* (Fabaceae)	Applications in Plant Sciences	Article	2168-0450	Apr 2015	3	4	NA	10.3732/apps.1500013
65	Itagaki, T et al.	Development of microsatellite markers for *Aquilegia buergeriana* var. *Oxysepala* (Ranunculaceae), a vulnerable Japanese herb	Plant Species Biology	Article	0913-557X	Apr 2015	30	2	159 162	10.1111/1442-1984.12044
66	Stack, JC et al.	Assessing microsatellite linkage disequilibrium in wild, cultivated, and mapping populations of *Theobroma cacao* L. and its impact on association mapping	Tree Genetics & Genomes	Article	1614-2942	Apr 2015	11	2	NA	10.1007/s11295-015-0839-0
67	Zarouri, B et al.	Whole-genome genotyping of grape using a panel of microsatellite multiplex PCRs	Tree Genetics & Genomes	Article	1614-2942	Apr 2015	11	2	NA	10.1007/s11295-015-0843-4
68	Tiwari, KK et al.	Identification of a diverse mini-core panel of Indian rice germplasm based on genotyping using microsatellite markers	Plant Breeding	Article	0179-9541	Apr 2015	134	2	164 171	10.1111/pbr.12252

	Author	Publication title	Source	Publication type	ISSN	Publication date		Vol	Issue	Pages		DOI
69	Iwazumi, MG et al.	Highly polymorphic nuclear microsatellite markers reveal detailed patterns of genetic variation in natural populations of Yezo spruce in Hokkaido	Journal of Forest Research	Article	1341-6979	Apr	2015	20	2	301	307	10.1007/s10310-014-0477-7
70	Manyasa, EO et al.	Genetic diversity in East African finger millet (*Eleusine coracana* (L.) Gaertn) landraces based on SSR markers and some qualitative traits	Plant Genetic Resources	Article	1479-2621	Apr	2015	13	1	45	55	10.1017/S1479262114000628
71	Armbruster, GFJ; Stocklin, J	New microsatellite markers for *Campanula scheuchzeri* (Campanulaceae), with cross-amplification in *C. Rotundifolia*	Applications in Plant Sciences	Article	2168-0450	Mar	2015	3	3	NA		10.3732/apps.1400118
72	Kameoka, S et al.	Development of polymorphic microsatellite loci in the perennial herb *Hepatica nobilis* var. *japonica* (Ranunculaceae)	Applications in Plant Sciences	Article	2168-0450	Mar	2015	3	3	NA		10.3732/apps.1400114
73	Qiang, Y et al.	Development of microsatellite markers for *Carallia brachiata* (Rhizophoraceae)	Applications in Plant Sciences	Article	2168-0450	Mar	2015	3	3	NA		10.3732/apps.1400125
74	Radosavljevic, I et al.	New microsatellite markers for *Campanula pyramidalis* (Campanulaceae) and cross-amplification in closely related species	Applications in Plant Sciences	Article	2168-0450	Mar	2015	3	3	NA		10.3732/apps.1400117
75	Nubankoh, P et al.	Genetic diversity and population structure of pencil yam (*Vigna lanceolata*) (Phaseoleae, Fabaceae), a wild herbaceous legume endemic to Australia, revealed by microsatellite markers	Botany	Article	1916-2790	Mar	2015	93	3	183	191	10.1139/cjb-2014-0222
76	Bajaj, D et al.	Genome-wide conserved non-coding microsatellite (CNMS) marker-based integrative genetical genomics for quantitative dissection of seed weight in chickpea	Journal of Experimental Botany	Article	0022-0957	Mar	2015	66	5	1271	1290	10.1093/jxb/eru478
77	Miao, Y-C et al.	Microsatellite markers indicate genetic differences between cultivated and natural populations of endangered *Taxus yunnanensis*	Botanical Journal of the Linnean Society	Article	0024-4074	Mar	2015	177	3	450	461	10.1111/boj.12249
78	Ravishankar, KV et al.	Development and characterization of microsatellite markers in mango (*Mangifera indica*) using next-generation sequencing technology and their transferability across species	Molecular Breeding	Article	1380-3743	Mar	2015	35	3	NA		10.1007/s11032-015-0289-2
79	Moscoe, LJ; Emshwiller, E	Diversity of *Oxalis tuberosa* Molina: a comparison between AFLP and microsatellite markers	Genetic Resources & Crop Evolution	Article	0925-9864	Mar	2015	62	3	335	347	10.1007/s10722-014-0154-x
80	Bakoume, C et al.	Genetic diversity of the world's largest oil palm (*Elaeis guineensis* Jacq.) field genebank accessions using microsatellite markers	Genetic Resources & Crop Evolution	Article	0925-9864	Mar	2015	62	3	349	360	10.1007/s10722-014-0156-8
81	Salem, KFM et al.	Assessing genetic diversity of Egyptian hexaploid wheat (*Triticum aestivum* L.) using microsatellite markers	Genetic Resources & Crop Evolution	Article	0925-9864	Mar	2015	62	3	377	385	10.1007/s10722-014-0159-5
82	Hua, W et al.	A study of genetic diversity of colored barley (*Hordeum vulgare* L.) using SSR markers	Genetic Resources & Crop Evolution	Article	0925-9864	Mar	2015	62	3	395	406	10.1007/s10722-014-0165-7
83	Filippi, CV et al.	Population structure and genetic diversity characterization of a sunflower association mapping population using SSR and SNP markers	BMC Plant Biology	Article	1471-2229	Feb	2015	15	NA	NA		10.1186/s12870-014-0360-x
84	Badgley, EM et al.	Microsatellite marker development for the coastal dune shrub *Prunus maritima* (Rosaceae)	Applications in Plant Sciences	Article	2168-0450	Feb	2015	3	2	NA		10.3732/apps.1400119
85	Duarte-Barbosa, M et al.	Development and characterization of 47 novel microsatellite markers for *Vellozia squamata* (Velloziaceae)	Applications in Plant Sciences	Article	2168-0450	Feb	2015	3	2	NA		10.3732/apps.1400087
86	Grando, C et al.	Development and characterization of microsatellite markers for *Piptadenia gonoacantha* (Fabaceae)	Applications in Plant Sciences	Article	2168-0450	Feb	2015	3	2	NA		10.3732/apps.1400107
87	Li, J-K et al.	Development and characterization of microsatellite loci for the pseudometallophyte *Commelina communis* (Commelinaceae)	Applications in Plant Sciences	Article	2168-0450	Feb	2015	3	2	NA		10.3732/apps.1400098
88	Zeisek, V et al.	Microsatellite variation, sexual reproduction and taxonomic revision of *Taraxacum* sect. *Dioszegia*: relationships at a large spatial scale	Preslia	Article	0032-7786	Feb	2015	87	1	55	85	NA
89	Marti, AFI et al.	Molecular analyses of evolution and population structure in a worldwide almond [*Prunus dulcis* (Mill.) DA Webb syn. *P. amygdalus* Batsch] pool assessed by microsatellite markers	Genetic Resources & Crop Evolution	Article	0925-9864	Feb	2015	62	2	205	219	10.1007/s10722-014-0146-x
90	Naik, BK et al.	Molecular mapping and validation of the microsatellite markers linked to the *Secale cereale*-	Molecular Breeding	Article	1380-3743	Feb	2015	35	2	NA		10.1007/s11032-015-0234-4

	Author	Publication title	Source	Publication type	ISSN	Publication date	Vol	Issue	Pages	DOI
		derived leaf rust resistance gene *Lr45* in wheat								
91	Wang, LX et al.	The transferability and polymorphism of mung bean SSR markers in rice bean germplasm	Molecular Breeding	Article	1380-3743	Feb 2015	35	2	NA	10.1007/s11032-015-0280-y
92	Liesebach, H et al.	FDR and SDR processes in meiosis and diploid gamete formation in poplars (*Populus* L.) detected by centromere-associated microsatellite markers	Tree Genetics & Genomes	Article	1614-2942	Feb 2015	11	1	NA	10.1007/s11295-014-0801-6
93	Kurokochi, H et al.	Development of 18 microsatellite markers in *Pieris japonica*, a poisonous tree insulated from the browsing pressure of herbivores, using a next-generation sequencer	Journal of Forest Research	Article	1341-6979	Feb 2015	20	1	244 247	10.1007/s10310-014-0456-z
94	Marti, AFI et al.	Genetic relationships and population structure of local olive tree accessions from Northeastern Spain revealed by SSR markers	Acta Physiologiae Plantarum	Article	0137-5881	Jan 2015	37	1	NA	10.1007/s11738-014-1726-2
95	Donkpegan, ASL et al.	Microsatellite development and flow cytometry in the african tree genus *Afzelia* (Fabaceae, Caesalpinioideae) reveal a polyploid complex	Applications in Plant Sciences	Article	2168-0450	Jan 2015	3	1	NA	10.3732/apps.1400097
96	Duwe, VK et al.	Fourteen polymorphic microsatellite markers for the threatened *Arnica montana* (Asteraceae)	Applications in Plant Sciences	Article	2168-0450	Jan 2015	3	1	NA	10.3732/apps.1400091
97	Gonzalez, C et al.	Development and characterization of microsatellite loci in the mistletoe *Psittacanthus schiedeanus* (Loranthaceae)	Applications in Plant Sciences	Article	2168-0450	Jan 2015	3	1	NA	10.3732/apps.1400099
98	Harris-Shultz, K et al.	Development and characterization of microsatellite markers for a little bluestem collection	Journal of theAmerican Society for Horticultural Science	Article	0003-1062	Jan 2015	140	1	78 87	NA
99	Addisalem, AB et al.	Genomic sequencing and microsatellite marker development for *Boswellia papyrifera*, an economically important but threatened tree native to dry tropical forests	AoB Plants	Article	2041-2851	NA 2015	7	NA	NA	10.1093/aobpla/plu086
100	Boccacci, P et al.	*In silico* mining, characterization and cross-species transferability of EST-SSR markers for European hazelnut (*Corylus avellana* L.)	Molecular Breeding	Article	1380-3743	Jan 2015	35	1	NA	10.1007/s11032-015-0195-7
101	Ganie, SA; Mondal, TK	Genome-wide development of novel miRNA-based microsatellite markers of rice (*Oryza sativa*) for genotyping applications	Molecular Breeding	Article	1380-3743	Jan 2015	35	1	NA	10.1007/s11032-015-0207-7
102	Verma, P et al.	Development, characterization and cross-species transferability of genomic SSR markers in berseem (*Trifolium alexandrinum* L.), an important multi-cut annual forage legume	Molecular Breeding	Article	1380-3743	Jan 2015	35	1	NA	10.1007/s11032-015-0223-7
103	Contreras-Negrete, G et al.	Genetic diversity and structure of wild and managed populations of *Polaskia chende* (Cactaceae) in the Tehuacan-Cuicatlan Valley, Central Mexico: insights from SSR and allozyme markers	Genetic Resources & Crop Evolution	Article	0925-9864	Jan 2015	62	1	85 101	10.1007/s10722-014-0137-y
104	Zhao, YL et al.	Genetic diversity and population structure of elite cotton (*Gossypium hirsutum* L.) germplasm revealed by SSR markers	Plant Systematics & Evolution	Article	0378-2697	Jan 2015	301	1	327 336	10.1007/s00606-014-1075-z
105	Xiao, Y et al.	Exploiting transcriptome data for the development and characterization of gene-based SSR markers related to cold tolerance in oil palm (*Elaeis guineensis*)	BMC Plant Biology	Article	1471-2229	Dec 2014	14	NA	NA	10.1186/s12870-014-0384-2
106	Caruso, T et al.	Genetic diversity and clonal variation within the main Sicilian olive cultivars based on morphological traits and microsatellite markers	Scientia Horticulturae	Article	0304-4238	Dec 2014	180	NA	130 138	10.1016/j.scienta.2014.10.019
107	von Crautlein, M et al.	Development and characterization of chloroplast microsatellite markers in a fine-leaved fescue, *Festuca rubra* (Poaceae)	Applications in Plant Sciences	Article	2168-0450	Dec 2014	2	12	NA	10.3732/apps.1400094
108	Wu, J et al.	Characterisation and development of EST-SSR markers in tree peony using transcriptome sequences	Molecular Breeding	Article	1380-3743	Dec 2014	34	4	1853 1866	10.1007/s11032-014-0144-x
109	Xanthopoulou, A et al.	Microsatellite high-resolution melting (SSR-HRM) analysis for genotyping and molecular characterization of an *Olea europaea* germplasm collection	Plant Genetic Resources	Article	1479-2621	Dec 2014	12	3	273 277	10.1017/S147926211400001X
110	Taniguchi, F et al.	Worldwide core collections of tea (*Camellia sinensis*) based on SSR markers	Tree Genetics & Genomes	Article	1614-2942	Dec 2014	10	6	1555 1565	10.1007/s11295-014-0779-0
111	Asari, NS et al.	Standalone EST microsatellite mining and analysis tool (SEMAT): for automated EST-SSR analysis in plants	Tree Genetics & Genomes	Article	1614-2942	Dec 2014	10	6	1755 1757	10.1007/s11295-014-0785-2

	Author	Publication title	Source	Publication type	ISSN	Publication date		Vol	Issue	Pages		DOI
112	Linden, L; Iwarsson, M	Identification of weeping crabapple cultivars by microsatellite DNA markers and morphological traits	Scientia Horticulturae	Article	0304-4238	Nov	2014	179	NA	221	226	10.1016/j.scienta.2014.09.027
113	Adhikari, P et al.	Interspecific hybrid identification of *Vitis aestivalis*-derived Norton-based populations using microsatellite markers	Scientia Horticulturae	Article	0304-4238	Nov	2014	179	NA	363	366	10.1016/j.scienta.2014.09.048
114	Tiwari, KK et al.	Allelic variation in the microsatellite marker locus RM6100 linked to fertility restoration of WA based male sterility in rice	Indian Journal of Genetics & Plant Breeding	Article	0019-5200	Nov	2014	74	4	409	413	10.5958/0975-6906.2014.00863.3
115	Sarkar, S et al.	Analysis of genetic diversity among the Indian bread wheat cultivars using microsatellite (SSR) markers	Indian Journal of Genetics &Plant Breeding	Article	0019-5200	Nov	2014	74	4	502	505	10.5958/0975-6906.2014.00877.3
116	Kolahi-Zonoozi, S et al.	Development of 12 new SSR markers for genetic diversity and structure analysis in pistachio (*Pistacia vera* L.)	Journal of Horticultural Science & Biotechnology	Article	1462-0316	Nov	2014	89	6	707	711	NA
117	Aguilar-Barajas, E et al.	Isolation and characterization of polymorphic microsatellite loci in *Spondias radlkoferi* (Anacardiaceae)	Applications in Plant Sciences	Article	2168-0450	Nov	2014	2	11	NA		10.3732/apps.1400079
118	Grubisha, LC et al.	Characterization of microsatellite markers for pinedrops, *Pterospora andromedea* (Ericaceae), from Illumina MiSeq sequencing	Applications in Plant Sciences	Article	2168-0450	Nov	2014	2	11	NA		10.3732/apps.1400072
119	van Dijk et al.	Development of multiplex microsatellite PCR panels for the seagrass *Thalassia hemprichii* (Hydrocharitaceae)	Applications in Plant Sciences	Article	2168-0450	Nov	2014	2	11	NA		10.3732/apps.1400078
120	Wu, J et al.	High-density genetic linkage map construction and identification of fruit-related QTLs in pear using SNP and SSR markers	Journal of Experimental Botany	Article	0022-0957	Nov	2014	65	20	5771	5781	10.1093/jxb/eru311
121	Sumathi, M; Yasodha, R	Microsatellite resources of *Eucalyptus*: current status and future perspectives	Botanical Studies	Review	1999-3110	Oct	2014	55	NA	NA		10.1186/s40529-014-0073-3
122	Tsai, CC et al.	Analysis of microsatellites in the vulnerable orchid *Gastrodia flavilabella*: the development of microsatellite markers, and cross-species amplification in *Gastrodia*	Botanical Studies	Article	1999-3110	Oct	2014	55	NA	NA		10.1186/s40529-014-0072-4
123	Bijak, AL et al.	Development of microsatellite markers for a tropical seagrass, *Syringodium filiforme* (Cymodoceaceae)	Applications in Plant Sciences	Article	2168-0450	Oct	2014	2	10	NA		10.3732/apps.1400082
124	Chatwin, WB et al.	Microsatellite primer development for post oak, *Quercus stellata* (Fagaceae)	Applications in Plant Sciences	Article	2168-0450	Oct	2014	2	10	NA		10.3732/apps.1400070
125	Lopez-Villalobos, A et al.	Microsatellite primers for *Camissoniopsis cheiranthifolia* (Onagraceae) and cross-amplification in related species	Applications in Plant Sciences	Article	2168-0450	Oct	2014	2	10	NA		10.3732/apps.1400057
126	Tan, ML et al.	Developing and characterising *Ricinus communis* SSR markers by data mining of whole-genome sequences	Molecular Breeding	Article	1380-3743	Oct	2014	34	3	893	904	10.1007/s11032-014-0083-6
127	Surapaneni, M et al.	Development and characterization of microsatellite markers in Indian sesame (*Sesamum indicum* L.)	Molecular Breeding	Article	1380-3743	Oct	2014	34	3	1185	1200	10.1007/s11032-014-0109-0
128	Semagn, K et al.	Genetic relationships and structure among open-pollinated maize varieties adapted to Eastern and Southern Africa using microsatellite markers	Molecular Breeding	Article	1380-3743	Oct	2014	34	3	1423	1435	10.1007/s11032-014-0126-z
129	Wu, TQ et al.	The first Illumina-based *de novo* transcriptome sequencing and analysis of pumpkin (*Cucurbita moschata* Duch.) and SSR marker development	Molecular Breeding	Article	1380-3743	Oct	2014	34	3	1437	1447	10.1007/s11032-014-0128-x
130	Raji, R et al.	Investigation of variability of apricot (*Prunus armeniaca* L.) using morphological traits and microsatellite markers	Scientia Horticulturae	Article	0304-4238	Sep	2014	176	NA	225	231	10.1016/j.scienta.2014.06.033
131	Liu, YC et al.	Exploiting EST databases for the development and characterization of EST-SSR markers in blueberry (*Vaccinium*) and their cross-species transferability in *Vaccinium* spp	Scientia Horticulturae	Article	0304-4238	Sep	2014	176	NA	319	329	10.1016/j.scienta.2014.07.026
132	Ribeiro, PCC et al.	Transferability and characterization of nuclear microsatellite markers in populations of *Annona coriacea* (Annonaceae), a tree from the Brazilian cerrado	Brazilian Journal of Botany	Article	1806-9959	Sep	2014	37	3	353	356	10.1007/s40415-014-0074-1
133	Bossu, A et al.	Microsatellite primers in *Parietaria judaica* (Urticaceae) to assess genetic diversity and structure in urban landscapes	Applications in Plant Sciences	Article	2168-0450	Sep	2014	2	9	NA		10.3732/apps.1400036
134	Byers, C et al.	Microsatellite primers in *Agave utahensis*	Applications in Plant Sciences	Article	2168-0450	Sep	2014	2	9	NA		10.3732/apps.1400047

	Author	Publication title	Source	Publication type	ISSN	Publication date	Vol	Issue	Pages	DOI
		(Asparagaceae), a keystone species in the Mojave desert and Colorado plateau								
135	Mochizuki, K et al.	Isolation and characterization of 11 microsatellite markers for *Glochidion acuminatum* (Phyllanthaceae)	Applications in Plant Sciences	Article	2168-0450	Sep 2014	2	9	NA	10.3732/apps.1400045
136	van der Meer, S et al.	Microsatellite primers for the gynodioecious grassland perennial *Saxifraga granulata* (Saxifragaceae)	Applications in Plant Sciences	Article	2168-0450	Sep 2014	2	9	NA	10.3732/apps.1400040
137	Wei, N; Dick, CW	Polymorphic microsatellite markers for a wind-dispersed tropical tree species, *Triplaris cumingiana* (Polygonaceae)	Applications in Plant Sciences	Article	2168-0450	Sep 2014	2	9	NA	10.3732/apps.1400051
138	Seeber, E et al.	Ploidy in the alpine sedge *Kobresia pygmaea* (Cyperaceae) and related species: combined application of chromosome counts, new microsatellite markers and flow cytometry	Botanical Journal of the Linnean Society	Article	0024-4074	Sep 2014	176	1	22 35	10.1111/boj.12189
139	Ferriol, M et al.	Microsatellite evidence for low genetic diversity and reproductive isolation in tetraploid *Centaurea seridis* (Asteraceae) coexisting with diploid *Centaurea aspera* and triploid hybrids in contact zones	Botanical Journal of the Linnean Society	Article	0024-4074	Sep 2014	176	1	82 98	10.1111/boj.12194
140	Linos, A et al.	Genetic structure of the Greek olive germplasm revealed by RAPD, ISSR and SSR markers	Scientia Horticulturae	Article	0304-4238	Aug 2014	175	NA	33 43	10.1016/j.scienta.2014.05.034
141	Kuwahara, K et al.	An analysis of genetic differentiation and geographical variation of spinach germplasm using SSR markers	Plant Genetic Resources	Article	1479-2621	Aug 2014	12	2	185 190	10.1017/S1479262113000464
142	Ince, AG et al.	New microsatellite and CAPS-microsatellite markers for clarifying taxonomic and phylogenetic relationships within *Origanum* L.	Molecular Breeding	Article	1380-3743	Aug 2014	34	2	643 654	10.1007/s11032-014-0064-9
143	Ouattara, B et al.	Genetic diversity of *Jatropha curcas* L. in Senegal compared with exotic accessions based on microsatellite markers	Genetic Resources & Crop Evolution	Article	0925-9864	Aug 2014	61	6	1039 1045	10.1007/s10722-014-0106-5
144	Muehlbauer, MF et al.	Characterization of Eastern filbert blight-resistant hazelnut germplasm using microsatellite markers	Journal of the American Society for Horticultural Science	Article	0003-1062	Jul 2014	139	4	399 432	NA
145	Laosatit, K et al.	Development of interspecific and intergeneric hybrids among *Jatropha*-related species and verification of the hybrids using EST-SSR markers	Plant Genetic Resources	Article	1479-2621	Jul 2014	12	NA	S58 S61	10.1017/S1479262114000276
146	Lee, GA et al.	Development of microsatellite markers at the National Agrobiodiversity Center in Korea for the genetic assessment of underutilized crops	Plant Genetic Resources	Article	1479-2621	Jul 2014	12	NA	S125 S129	10.1017/S1479262114000525
147	Fugate, KK et al.	Generation and characterization of a sugarbeet transcriptome and transcript-based SSR markers	Plant Genome	Article	1940-3372	Jul 2014	7	2	NA	10.3835/plantgenome2013.11.0038
148	Hamann, E et al.	Novel microsatellite markers for the high-alpine *Geum reptans* (Rosaceae)	Applications in Plant Sciences	Article	2168-0450	Jun 2014	2	6	NA	10.3732/apps.1400021
149	Klabunde, GHF et al.	Characterization of 10 new nuclear microsatellite markers in *Acca sellowiana* (Myrtaceae)	Applications in Plant Sciences	Article	2168-0450	Jun 2014	2	6	NA	10.3732/apps.1400020
150	Lassen, KM et al.	Microsatellite primers for *Parkia biglobosa* (Fabaceae: Mimosoideae) reveal that a single plant sires all seeds per pod	Applications in Plant Sciences	Article	2168-0450	Jun 2014	2	6	NA	10.3732/apps.1400024
151	Fayyaz, L et al.	Genetic diversity analysis of *Brassica napus*/*Brassica campestris* progenies using microsatellite markers	Pakistan Journal of Botany	Article	0556-3321	Jun 2014	46	3	779 787	NA
152	Kujur, A et al.	An efficient and cost-effective approach for genic microsatellite marker-based large-scale trait association mapping: identification of candidate genes for seed weight in chickpea	Molecular Breeding	Article	1380-3743	Jun 2014	34	1	241 265	10.1007/s11032-014-0033-3
153	Balas, FC et al.	*Ex situ* conservation of underutilised fruit tree species: establishment of a core collection for *Ficus carica* L. using microsatellite markers (SSRs)	Tree Genetics & Genomes	Article	1614-2942	Jun 2014	10	3	703 710	10.1007/s11295-014-0715-3
154	Postolache, D et al.	Transcriptome versus genomic microsatellite markers: highly informative multiplexes for genotyping *Abies alba* Mill. and congeneric species	Plant Molecular Biology Reporter	Article	0735-9640	Jun 2014	32	3	750 760	10.1007/s11105-013-0688-7
155	Mallor, C et al.	Assessing the genetic diversity of Spanish *Allium cepa* landraces for onion breeding using microsatellite markers	Scientia Horticulturae	Article	0304-4238	May 2014	170	NA	24 31	10.1016/j.scienta.2014.02.040
156	Ahn, YK et al.	Microsatellite marker information from high-throughput next-generation sequence data of *Capsicum annuum* varieties Mandarin and Blackcluster	Scientia Horticulturae	Article	0304-4238	May 2014	170	NA	123 130	10.1016/j.scienta.2014.03.007

	Author	Publication title	Source	Publication type	ISSN	Publication date	Vol	Issue	Pages	DOI
157	Ando, H et al.	Development of microsatellite markers for the coastal shrub *Scaevola taccada* (Goodeniaceae)	Applications in Plant Sciences	Article	2168-0450	May 2014	2	5	NA	10.3732/apps.1300094
158	Bernardes, V et al.	Isolation and characterization of microsatellite loci in *Byrsonima cydoniifolia* (Malpighiaceae) and cross-amplification in *B. crassifolia*	Applications in Plant Sciences	Article	2168-0450	May 2014	2	5	NA	10.3732/apps.1400016
159	Kameyama, Y; Hirao, AS	Development and evaluation of microsatellite markers for the gynodioecious shrub *Daphne jezoensis* (Thymelaeaceae)	Applications in Plant Sciences	Article	2168-0450	May 2014	2	5	NA	10.3732/apps.1400001
160	Lu, YB et al.	Microsatellite markers for the invasive species *Bidens alba* (Asteraceae)	Applications in Plant Sciences	Article	2168-0450	May 2014	2	5	NA	10.3732/apps.1400008
161	Yamauchi, S; Ohsako, T	Isolation and characterization of microsatellite loci in *Fimbristylis sericea* (cyperaceae)	Applications in Plant Sciences	Article	2168-0450	May 2014	2	5	NA	10.3732/apps.1400026
162	Martinez-Castillo, J et al.	Genetic structure within the Mesoamerican gene pool of wild *Phaseolus lunatus* (Fabaceae) from Mexico as revealed by microsatellite markers: implications for conservation and the domestication of the species	American Journal of Botany	Article	0002-9122	May 2014	101	5	851 864	10.3732/ajb.1300412
163	Dias, EF et al.	Microsatellite markers unravel the population genetic structure of the *Azorean leontodon*: implications in conservation	Plant Systematics & Evolution	Article	0378-2697	May 2014	300	5	987 1001	10.1007/s00606-013-0937-0
164	Hodaei, M et al.	Plasmon analysis in wheat alloplasmic lines using morphological and chloroplast microsatellite markers	Plant Systematics & Evolution	Article	0378-2697	May 2014	300	5	1137 1145	10.1007/s00606-013-0951-2
165	Satya, P et al.	Comparative analysis of diversification and population structure of kenaf (*Hibiscus cannabinus* L.) and roselle (*H. sabdariffa* L.) using SSR and RGA (Resistance Gene Analogue) markers	Plant Systematics & Evolution	Article	0378-2697	May 2014	300	5	1209 1218	10.1007/s00606-013-0956-x
166	Lee, JH et al.	Genetic differentiation and introgression among Korean evergreen *Quercus* (Fagaceae) are revealed by microsatellite markers	Annales Botanici Fennici	Article	0003-3847	Apr 2014	51	1-2	39 48	NA
167	Krishnan, RR et al.	Microsatellite marker analysis reveals the events of the introduction and spread of cultivated mulberry in the Indian subcontinent	Plant Genetic Resources	Article	1479-2621	Apr 2014	12	1	129 139	10.1017/S1479262113000415
168	Sarao, NK et al.	Microsatellite-based DNA fingerprinting and genetic diversity of bottle gourd genotypes	Plant Genetic Resources	Article	1479-2621	Apr 2014	12	1	156 159	10.1017/S1479262113000385
169	Ioannis, G et al.	Microsatellite high-resolution melting (SSR-HRM) analysis for identification of sweet cherry rootstocks in Greece	Plant Genetic Resources	Article	1479-2621	Apr 2014	12	1	160 163	10.1017/S1479262113000403
170	Beck, JB et al.	Genus-wide microsatellite primers for the goldenrods (*Solidago*; Asteraceae)	Applications in Plant Sciences	Article	2168-0450	Apr 2014	2	4	NA	10.3732/apps.1300093
171	El Bahloul, Y et al.	Development and characterization of microsatellite loci for the Moroccan endemic endangered species *Argania spinosa* (sapotaceae)	Applications in Plant Sciences	Article	2168-0450	Apr 2014	2	4	NA	10.3732/apps.1300071
172	Hughes, PW et al.	Development of polymorphic microsatellite markers for Indian tobacco, *Lobelia inflata* (Campanulaceae)	Applications in Plant Sciences	Article	2168-0450	Apr 2014	2	4	NA	10.3732/apps.1300096
173	Nock, CJ et al.	Whole genome shotgun sequences for microsatellite discovery and application in cultivated and wild *Macadamia* (Proteaceae)	Applications in Plant Sciences	Article	2168-0450	Apr 2014	2	4	NA	10.3732/apps.1300089
174	Ohtsuki, T et al.	Development of microsatellite markers for *Vitex rotundifolia* (Verbenaceae), an endangered coastal plant in lake biwa, Japan	Applications in Plant Sciences	Article	2168-0450	Apr 2014	2	4	NA	10.3732/apps.1300100
175	Yoshida, NC et al.	Isolation and characterization of nine polymorphic microsatellite loci in *Piper solmsianum* (Piperaceae)	Applications in Plant Sciences	Article	2168-0450	Apr 2014	2	4	NA	10.3732/apps.1300092
176	Saito, Y et al.	Isolation and characterisation of eight microsatellite markers in *Paraserianthes falcataria*, a fast-growing tropical leguminous tree species	Journal of Tropical Forest Science	Editorial Material	0128-1283	Apr 2014	26	2	295 297	NA
177	Jannatabadi, AA et al.	Genetic diversity of Iranian landrace chickpea (*Cicer arietinum* L.) accessions from different geographical origins as revealed by morphological and sequence tagged microsatellite markers	Journal of Plant Biochemistry And Biotechnology	Article	0971-7811	Apr 2014	23	2	225 229	10.1007/s13562-013-0206-x
178	Bai, TD et al.	Characterization of masson pine (*Pinus massoniana* Lamb.) microsatellite DNA by 454 genome shotgun sequencing	Tree Genetics & Genomes	Article	1614-2942	Apr 2014	10	2	429 437	10.1007/s11295-013-0684-y
179	Jelinkova, H et al.	The use of digital morphometrics and spring phenology for clone recognition in trembling aspen	Trees-Structure And Function	Article	0931-1890	Apr 2014	28	2	389 398	10.1007/s00468-013-0957-y

	Author	Publication title	Source	Publication type	ISSN	Publication date	Vol	Issue	Pages	DOI
		(*Populus tremuloides* Michx.) and its comparison to microsatellite markers								
180	Moses, M et al.	Microsatellite based analysis of the genetic structure and diversity of *Capsicum chinense* in the Neotropics	Genetic Resources & Crop Evolution	Article	0925-9864	Apr 2014	61	4	741 755	10.1007/s10722-013-0069-y
181	Sitther, V et al.	Genetic characterization of guava (*Psidium guajava* L.) germplasm in the United States using microsatellite markers	Genetic Resources & Crop Evolution	Article	0925-9864	Apr 2014	61	4	829 839	10.1007/s10722-014-0078-5
182	Pineda-Martos, R et al.	Identification, characterisation and discriminatory power of microsatellite markers in the parasitic weed *Orobanche cumana*	Weed Research	Article	0043-1737	Apr 2014	54	2	120 132	10.1111/wre.12062
183	Yang, T et al.	Large-scale microsatellite development in grasspea (*Lathyrus sativus* L.), an orphan legume of the arid areas	BMC Plant Biology	Article	1471-2229	Mar 2014	14	NA	NA	10.1186/1471-2229-14-65
184	Aoki-Goncalves, F et al.	Microsatellite loci for *Orthophytum ophiuroides* (Bromelioideae, Bromeliaceae) species adapted to neotropical rock outcrops	Applications in Plant Sciences	Article	2168-0450	Mar 2014	2	3	NA	10.3732/apps.1300073
185	Manoel, RO et al.	Development and characterization of 32 microsatellite loci in *Genipa americana* (Rubiaceae)	Applications in Plant Sciences	Article	2168-0450	Mar 2014	2	3	NA	10.3732/apps.1300084
186	Pan, L et al.	Development of 12 chloroplast microsatellite markers in *Vigna unguiculata* (Fabaceae) and amplification in *Phaseolus vulgaris*	Applications in Plant Sciences	Article	2168-0450	Mar 2014	2	3	NA	10.3732/apps.1300075
187	Van Etten, ML et al.	*Sophora microphylla* (Fabaceae) microsatellite markers and their utility across the genus	Applications in Plant Sciences	Article	2168-0450	Mar 2014	2	3	NA	10.3732/apps.1300081
188	Nemati, Z et al.	Phylogenetic relationships among Iranian and Spanish date palms (*Phoenix dactylifera* L.) revealed by microsatellite markers	Journal of Horticultural Science & Biotechnology	Article	1462-0316	Mar 2014	89	2	114 120	NA
189	Fakhrian, P et al.	Assessment of genetic diversity and genetic relationships among 46 Iranian and non-Iranian dwarfing rootstocks of apple (*Malus* X *domestica* Borkh.) using microsatellite markers	Journal of Horticultural Science & Biotechnology	Article	1462-0316	Mar 2014	89	2	121 129	NA
190	Miao, YC et al.	Phylogeography and genetic effects of habitat fragmentation on endangered *Taxus yunnanensis* in southwest China as revealed by microsatellite data	Plant Biology	Article	1435-8603	Mar 2014	16	2	365 374	10.1111/plb.12059
191	Talve, T et al.	Population genetic diversity and species relationships in the genus *Rhinanthus* L. based on microsatellite markers	Plant Biology	Article	1435-8603	Mar 2014	16	2	495 502	10.1111/plb.12057
192	Mena, A et al.	Recovery, identification and relationships by microsatellite analysis of ancient grapevine cultivars from Castilla-La Mancha: the largest wine growing region in the world	Genetic Resources & Crop Evolution	Article	0925-9864	Mar 2014	61	3	625 637	10.1007/s10722-013-0064-3
193	Yahya, AF et al.	Genetic variation and population genetic structure of *Rhizophora apiculata* (Rhizophoraceae) in the greater Sunda Islands, Indonesia using microsatellite markers	Journal of Plant Research	Article	0918-9440	Mar 2014	127	2	287 297	10.1007/s10265-013-0613-z
194	Wang, Z et al.	Mining new microsatellite markers for Siberian apricot (*Prunus sibirica* L.) From SSR-enriched genomic library	Scientia Horticulturae	Article	0304-4238	Feb 2014	166	NA	65 69	10.1016/j.scienta.2013.12.004
195	Zhao, ZQ et al.	Genetic diversity and relationships among loose-curd cauliflower and related varieties as revealed by microsatellite markers	Scientia Horticulturae	Article	0304-4238	Feb 2014	166	NA	105 110	10.1016/j.scienta.2013.12.024
196	Cerqueira-Silva, CBM et al.	New microsatellite markers for wild and commercial species of *Passiflora* (Passifloraceae) and cross-amplification	Applications in Plant Sciences	Article	2168-0450	Feb 2014	2	2	NA	10.3732/apps.1300061
197	Flores, CG et al.	Development and characterization of 10 microsatellite loci in the giant cardon cactus, *Pachycereus pringlei* (Cactaceae)	Applications in Plant Sciences	Article	2168-0450	Feb 2014	2	2	NA	10.3732/apps.1300066
198	Petersen, JJ et al.	Ten polymorphic microsatellite primers in the tropical tree caimito, *Chrysophyllum cainito* (Sapotaceae)	Applications in Plant Sciences	Article	2168-0450	Feb 2014	2	2	NA	10.3732/apps.1300079
199	Rocha, OJ et al.	Isolation and characterization of microsatellite loci from amur honeysuckle, *Lonicera maackii* (Caprifoliaceae)	Applications in Plant Sciences	Article	2168-0450	Feb 2014	2	2	NA	10.3732/apps.1300030
200	Wang, RH et al.	Development of microsatellite loci in *Scrophularia incisa* (Scrophulariaceae) and cross-amplification in congeneric species	Applications in Plant Sciences	Article	2168-0450	Feb 2014	2	2	NA	10.3732/apps.1300077

	Author	Publication title	Source	Publication type	ISSN	Publication date	Vol	Issue	Pages	DOI
201	Sivaranjani, R et al.	Microsatellite-based genetic diversity in selected exotic and indigenous maize (*Zea mays* L.) inbred lines differing in total kernel carotenoids	Indian Journal of Genetics & Plant Breeding	Article	0019-5200	Feb 2014	74	1	34 41	10.5958/j.0975-6906.74.1.005
202	Amagai, Y et al.	Microsatellite mapping of genes for branched spike and soft glumes in *Triticum monococcum* L.	Genetic Resources & Crop Evolution	Article	0925-9864	Feb 2014	61	2	465 471	10.1007/s10722-013-0050-9
203	Amagai, Y et al.	Microsatellite mapping of the genes for sham ramification and extra glume in spikelets of tetraploid wheat	Genetic Resources & Crop Evolution	Article	0925-9864	Feb 2014	61	2	491 498	10.1007/s10722-013-0052-7
204	Moriguchi, Y et al.	Establishment of a microsatellite panel covering the sugi (*Cryptomeria japonica*) genome, and its application for localization of a male-sterile gene (*ms-2*)	Molecular Breeding	Article	1380-3743	Feb 2014	33	2	315 325	10.1007/s11032-013-9951-8
205	Tomar, RSS et al.	Development of chloroplast-specific microsatellite markers for molecular characterization of alloplasmic lines and phylogenetic analysis in wheat	Plant Breeding	Article	0179-9541	Feb 2014	133	1	12 18	10.1111/pbr.12116
206	Nandha, PS; Singh, J	Comparative assessment of genetic diversity between wild and cultivated barley using gSSR and EST-SSR markers	Plant Breeding	Article	0179-9541	Feb 2014	133	1	28 35	10.1111/pbr.12118
207	Stajner, N et al.	Microsatellite inferred genetic diversity and structure of Western Balkan grapevines (*Vitis vinifera* L.)	Tree Genetics & Genomes	Article	1614-2942	Feb 2014	10	1	127 140	10.1007/s11295-013-0670-4
208	Trujillo, I et al.	Identification of the worldwide olive germplasm bank of Cordoba (Spain) using SSR and morphological markers	Tree Genetics & Genomes	Article	1614-2942	Feb 2014	10	1	141 155	10.1007/s11295-013-0671-3
209	Melo, WMC et al.	Genetic control of the performance of maize hybrids using complex pedigrees and microsatellite markers	Euphytica	Article	0014-2336	Feb 2014	195	3	331 344	10.1007/s10681-013-0999-7
210	Cosson, P et al.	Development and characterization of 96 microsatellite markers suitable for QTL mapping and accession control in an *Arabidopsis* core collection	Plant Methods	Article	1746-4811	Jan 2014	10	NA	NA	10.1186/1746-4811-10-2
211	Zhao, DW et al.	Genetic diversity and domestication origin of tea plant *Camellia taliensis* (Theaceae) as revealed by microsatellite markers	BMC Plant Biology	Article	1471-2229	Jan 2014	14	NA	NA	10.1186/1471-2229-14-14
212	Rawat, A et al.	Association mapping for resin yield in *Pinus roxburghii* Sarg. using microsatellite markers	Silvae Genetica	Article	0037-5349	NA 2014	63	6	253 266	NA
213	Hiraoka, Y et al.	Evaluation of the growth traits of *Toxicodendron vernicifluum* progeny based on their genetic groups assigned using new microsatellite markers	Silvae Genetica	Article	0037-5349	NA 2014	63	6	267 274	NA
214	Eusemann, P et al.	Three microsatellite multiplex PCR assays allowing high resolution genotyping of white spruce, *Picea glauca*	Silvae Genetica	Article	0037-5349	NA 2014	63	5	230 234	NA
215	Medina-Macedo, L et al.	Investigating the Mendelian inheritance, genetic linkage, and genotypic disequilibrium for ten microsatellite loci of *Araucaria angustifolia*	Silvae Genetica	Article	0037-5349	NA 2014	63	5	234 239	NA
216	Gandara, FB et al.	Development and characterization of microsatellite loci for *Cedrela fissilis* Vell (Meliaceae), an endangered tropical tree species	Silvae Genetica	Article	0037-5349	NA 2014	63	5	240 243	NA
217	Fritzmann, C et al.	A microsatellite (SSR)-based genetic method to identify strawberry (*Fragaria* x *ananassa*) cultivars	VII International Strawberry Symposium, Acta Horticulturae	Proceedings Paper	0567-7572BN 978-94-62610-37-8	Feb 2014	1049	NA	359 364	NA
218	Torres, MR et al.	Development of a microsatellite database for identification of olive (*Olea europaea* L.) cultivars in Mendoza, Argentina	VII International Symposium on Olive Growing, Acta Horticulturae	Proceedings Paper	0567-7572BN 978-94-62610-47-7	Sep 2014	1057	NA	521 524	NA
219	Essalouh, L et al.	Genomic and EST microsatellite loci development and use in olive: Molecular tools for genetic mapping and association studies	VII International Symposium on Olive Growing, Acta Horticulturae	Proceedings Paper	0567-7572BN 978-94-62610-47-7	Sep 2014	1057	NA	543 550	NA
220	Wang, LX et al.	Detecting seed purity of wheat varieties using microsatellite markers based on eliminating the influence of non-homozygous loci	Seed Science & Technology	Article	0251-0952	NA 2014	42	3	393 413	NA
221	Oppong, A et al.	Bulk genetic characterization of Ghanaian maize landraces using microsatellite markers	Maydica	Article	0025-6153	NA 2014	59	1-4	1 8	NA
222	Zhang, DD et al.	Short Note: Isolation and characterization of 12 polymorphic microsatellite markers in *Engelhardia roxburghiana* (Juglandaceae)	Silvae Genetica	Article	0037-5349	NA 2014	63	3	109 112	NA
223	Leisova-Svobodova, L et al.	The application of microsatellite analysis in barley	Czech Journal of Genetics &	Article	1212-1975	NA 2014	50	4	268 277	NA

	Author	Publication title	Source	Publication type	ISSN	Publication date		Vol	Issue	Pages		DOI
		malting quality breeding programmes	Plant Breeding									
224	Pardo, C et al.	Development and multiplexing of the first microsatellite markers in a coralline red alga (*Phymatolithon calcareum*, Rhodophyta)	Phycologia	Article	0031-8884	NA	2014	53	5	474	479	10.2216/14-031.1
225	Tsai, CC	Identification and characterization of 16 polymorphic microsatellite markers from *Mangifera indica* L. (Anacardiaceae)	II International Symposium on Biotechnology of Fruit Species Acta Horticulturae	Proceedings Paper	0567-7572BN 978-94-62610-36-1	Mar	2014	1048	NA	187	192	NA
226	Jahnke, G et al.	Analysis of grape rootstocks by microsatellite markers	X International Conference on Grapevine Breeding & Genetics, Acta Horticulturae	Proceedings Paper	0567-7572BN 978-94-62610-34-7	Aug	2014	1046	NA	617	626	NA
227	Jahnke, G et al.	Analysis of pinot cultivars by microsatellite markers	X International Conference on Grapevine Breeding & Genetics, Acta Horticulturae	Proceedings Paper	0567-7572BN 978-94-62610-34-7	Aug	2014	1046	NA	627	638	NA
228	Yamamoto, S et al.	Morphological and microsatellite analysis of putative natural hybrid population between *Lilium japonicum* and *L. auratum* in Izu Peninsula, Japan	III International Symposium on the Genus *Liliumse*, Acta Horticulturae	Proceedings Paper	0567-7572BN 978-94-62610-05-7	Apr	2014	1027	NA	47	54	NA
229	Madhou, M et al.	Comparison of accessions conserved in different litchi germplasm collections using microsatellite markers	IV International Symposium on Lychee, Longan and other Sapindaceae Fruits, Acta Horticulturae	Proceedings Paper	0567-7572BN 978-94-62610-15-6	Dec	2014	1029	NA	93	99	NA
230	Xiang, X et al.	Core EST-SSR marker selection based on genetic linkage map construction and their application in genetic diversity analysis of litchi (*Litchi chinensis* Sonn.) germplasm resources	IV International Symposium on Lychee, Longan and other Sapindaceae Fruits, Acta Horticulturae	Proceedings Paper	0567-7572BN 978-94-62610-15-6	Dec	2014	1029	NA	109	115	NA
231	Salazar, JA et al.	Random Amplified Microsatellite Polymorphism (RAMP) application in *Prunus* characterization and mapping	VI International Symposium on Almonds and Pistachiosse, Acta Horticulturae	Proceedings Paper	0567-7572BN 978-94-62610-13-2	May	2014	1028	NA	61	64	NA
232	Ahrens, CW; James, EA	Characterization of 13 microsatellite markers for *Diuris basaltica* (Orchidaceae) and related species	Applications in Plant Sciences	Article	2168-0450	Jan	2014	2	1	NA		10.3732/apps.1300069
233	Khan, G et al.	Isolation of 16 microsatellite markers for *Spiraea alpina* and *S. mongolica* (Rosaceae) of the qinghai tibet plateau	Applications in Plant Sciences	Article	2168-0450	Jan	2014	2	1	NA		10.3732/apps.1300059
234	Singh, T	Development and characterization of microsatellite markers in *Sagina nodosa* (Caryophyllacea)	Applications in Plant Sciences	Article	2168-0450	Jan	2014	2	1	NA		10.3732/apps.1300064
235	Ovesna, J et al.	Microsatellite analysis indicates the specific genetic basis of Czech bolting garlic	Czech Journal of Genetics & Plant Breeding	Article	1212-1975	NA	2014	50	3	226	234	NA
236	Tiwari, JK et al.	Assessment of genetic purity of four rice cultivars using microsatellite and ISSR markers	Seed Science & Technology	Article	0251-0952	NA	2014	42	2	227	236	NA
237	Mayer, C et al.	Development and multiplexing of microsatellite markers using pyrosequencing in a tetraploid plant, *Vaccinium uliginosum* (Ericaceae)	Plant Ecology & Evolution	Article	2032-3913	NA	2014	147	2	285	289	10.5091/plecevo.2014.831
238	Celikkol Akcay, U et al.	Genetic stability in a predominating Turkish olive cultivar, Gemlik, assessed by RAPD, microsatellite, and AFLP marker systems	Turkish Journal of Botany	Article	1300-008X	NA	2014	38	3	430	438	10.3906/bot-1309-23
239	Gilmore, BS et al.	Short-read DNA sequencing yields microsatellite markers for *Rheum*	Journal of the American Society for Horticultural Science	Article	0003-1062	Jan	2014	139	1	22	29	NA
240	Li, PB et al.	Cytoplasmic diversity of the cotton genus as revealed by chloroplast microsatellite markers	Genetic Resources & Crop Evolution	Article	0925-9864	Jan	2014	61	1	107	119	10.1007/s10722-013-0018-9
241	Achrem, M et al.	Assessment of genetic relationships among *Secale* taxa by using ISSR and IRAP markers and the chromosomal distribution of the AAC microsatellite sequence	Turkish Journal of Botany	Article	1300-008X	NA	2014	38	2	213	225	10.3906/bot-1207-26
242	Raghami, M et al.	Genetic diversity among melon accessions from Iran and their relationships with melon germplasm of diverse origins using microsatellite markers	Plant Systematics & Evolution	Article	0378-2697	Jan	2014	300	1	139	151	10.1007/s00606-013-0866-y
243	Mudalkar, S et al.	De novo transcriptome analysis of an imminent biofuel crop, *Camelina sativa* L. using Illumina GAIIX sequencing platform and identification of SSR markers	Plant Molecular Biology	Article	0167-4412	Jan	2014	84	1-2	159	171	10.1007/s11103-013-0125-1
244	Beghe, D et al.	Identification and characterization of ancient Italian chestnut using nuclear microsatellite markers	Scientia Horticulturae	Article	0304-4238	Dec	2013	164	NA	50	57	10.1016/j.scienta.2013.09.009
245	Iquebal, MA et al.	First whole genome based microsatellite DNA marker database of tomato for mapping and variety	BMC Plant Biology	Article	1471-2229	Dec	2013	13	NA	NA		10.1186/1471-2229-13-197

	Author	Publication title	Source	Publication type	ISSN	Publication date		Vol	Issue	Pages		DOI
		identification										
246	Arroyo, JM et al.	Isolation of 18 microsatellite loci in the desert mistletoe *Phoradendron californicum* (Santalaceae) via 454 pyrosequencing	Applications in Plant Sciences	Article	2168-0450	Dec	2013	1	12	NA		10.3732/apps.1300048
247	Fan, Q et al.	Development and characterization of microsatellite markers from the transcriptome of *Firmiana danxiaensis* (Malvaceae s.l.)	Applications in Plant Sciences	Article	2168-0450	Dec	2013	1	12	NA		10.3732/apps.1300047
248	Kesselring, H et al.	New microsatellite markers for *Anthyllis vulneraria* (Fabaceae), analyzed with spreadex gel electrophoresis	Applications in Plant Sciences	Article	2168-0450	Dec	2013	1	12	NA		10.3732/apps.1300054
249	Kissling, J et al.	Novel microsatellite loci for *Sebaea aurea* (Gentianaceae) and cross-amplification in related species	Applications in Plant Sciences	Article	2168-0450	Dec	2013	1	12	NA		10.3732/apps.1300056
250	Zhang, LR et al.	Development of 12 polymorphic microsatellite loci in the high alpine perennial *Primula halleri* (Primulaceae)	Applications in Plant Sciences	Article	2168-0450	Dec	2013	1	12	NA		10.3732/apps.1300052
251	Zhang, PF et al.	Development and characterization of 11 polymorphic microsatellite markers in *Tapiscia sinensis* (Staphyleaceae)	Applications in Plant Sciences	Article	2168-0450	Dec	2013	1	12	NA		10.3732/apps.1300051
252	Jani, TR et al.	Chloroplast microsatellite based molecular and computational studies in selected bamboo species	Vegetos	Article	0970-4078	Dec	2013	26	2	403	415	10.5958/j.2229-4473.26.2.105
253	Upadhyay, A et al.	Microsatellite analysis to rationalize grape germplasm in India and development of a molecular database	Plant Genetic Resources	Article	1479-2621	Dec	2013	11	3	225	233	10.1017/S1479262113000117
254	Juntheikki-Palovaara, I et al.	Microsatellite markers for common lilac (*Syringa vulgaris* L.)	Plant Genetic Resources	Article	1479-2621	Dec	2013	11	3	279	282	10.1017/S1479262113000166
255	Javed, MA et al.	Construction of microsatellite linkage map and detection of segregation distortion in *indica* rice (*Oryza sativa* L.)	Pakistan Journal of Botany	Article	0556-3321	Dec	2013	45	6	2085	2092	NA
256	Mathithumilan, B et al.	Development and characterization of microsatellite markers for *Morus* spp. and assessment of their transferability to other closely related species	BMC Plant Biology	Article	1471-2229	Dec	2013	13	NA	NA		10.1186/1471-2229-13-194
257	Adamski, DJ et al.	Cross-amplification of nonnative *Acacia* species in the Hawaiian Islands using microsatellite markers from *Acacia koa*	Plant Biosystems	Article	1126-3504	Dec	2013	147	4	1088	1091	10.1080/11263504.2012.749958
258	Tsy, JMLP et al.	Nuclear microsatellite variation in Malagasy baobabs (*Adansonia*, Bombacoideae, Malvaceae) reveals past hybridization and introgression	Annals of Botany	Article	0305-7364	Dec	2013	112	9	1759	1773	10.1093/aob/mct230
259	Penha, HA et al.	Development of microsatellite markers in sweet passion fruit, and identification of length and conformation polymorphisms within repeat sequences	Plant Breeding	Article	0179-9541	Dec	2013	132	6	731	735	10.1111/pbr.12083
260	Bhardwaj, P et al.	Development and utilization of genomic and genic microsatellite markers in Assam tea (*Camellia assamica* ssp *assamica*) and related *Camellia* species	Plant Breeding	Article	0179-9541	Dec	2013	132	6	748	763	10.1111/pbr.12101
261	Wang, YL	Chloroplast microsatellite diversity of *Opisthopappus* Shih (Asteraceae) endemic to China	Plant Systematics & Evolution	Article	0378-2697	Dec	2013	299	10	1849	1858	10.1007/s00606-013-0840-8
262	Motilal, LA et al.	Microsatellite-aided detection of genetic redundancy improves management of the International Cocoa Genebank, Trinidad	Tree Genetics & Genomes	Article	1614-2942	Dec	2013	9	6	1395	1411	10.1007/s11295-013-0645-5
263	Bruegmann, T; Fladung, M	Potentials and limitations of the cross-species transfer of nuclear microsatellite marker in six species belonging to three sections of the genus *Populus* L.	Tree Genetics & Genomes	Article	1614-2942	Dec	2013	9	6	1413	1421	10.1007/s11295-013-0647-3
264	Zhang, JJ et al.	Microsatellite genetic variation in the Chinese endemic *Eucommia ulmoides* (Eucommiaceae): implications for conservation	BotanicalJournal of the Linnean Society	Article	0024-4074	Dec	2013	173	4	775	785	10.1111/boj.12116
265	Liu, LL et al.	Development and integration of EST-SSR markers into an established linkage map in switchgrass	Molecular Breeding	Article	1380-3743	Dec	2013	32	4	923	931	10.1007/s11032-013-9921-1
266	Yamada, Y et al.	Microsatellite variability of sulfonylurea-resistant and susceptible populations of *Schoenoplectus juncoides* (Cyperaceae) in Kinki, Japan	Weed Research	Article	0043-1737	Dec	2013	53	6	429	439	10.1111/wre.12049
267	Moura, EF et al.	Identification of duplicates of cassava accessions sampled on the North Region of Brazil using microsatellite markers	Acta Amazonica	Article	0044-5967	Dec	2013	43	4	461	467	NA
268	Ahn, YK et al.	*De novo* transcriptome assembly and novel	Botanical Studies	Article	1999-3110	Nov	2013	54	NA	NA		10.1186/1999-3110-54-58

	Author	Publication title	Source	Publication type	ISSN	Publication date	Vol	Issue	Pages	DOI
		microsatellite marker information in *Capsicum annuum* varieties Saengryeg211 and Saengryeg213								
269	Ahrens, CW; James, EA	Characterization of microsatellite markers for the vulnerable grassland forb *Senecio macrocarpus* (Asteraceae)	Applications in Plant Sciences	Article	2168-0450	Nov 2013	1	11	NA	10.3732/apps.1300041
270	Hodkinson, TR et al.	Nuclear SSR markers for *Miscanthus*, *Saccharum*, and related grasses (Saccharinae, Poaceae)	Applications in Plant Sciences	Article	2168-0450	Nov 2013	1	11	NA	10.3732/apps.1300042
271	Ison, JL et al.	Development and evaluation of microsatellite markers for a native prairie perennial, *Echinacea angustifolia* (Asteraceae)	Applications in Plant Sciences	Article	2168-0450	Nov 2013	1	11	NA	10.3732/apps.1300049
272	Reis, TS et al.	Characterization of 10 microsatellite loci for *Bathysa australis* (Rubiaceae)	Applications in Plant Sciences	Article	2168-0450	Nov 2013	1	11	NA	10.3732/apps.1300055
273	Waza, SA et al.	Fingerprinting and purity testing of rice hybrids using microsatellite markers	Indian Journal of Genetics & Plant Breeding	Article	0019-5200	Nov 2013	73	4	443 445	10.5958/j.0975-6906.73.4.067
274	Innark, P et al.	Evaluation of genetic diversity in cucumber (*Cucumis sativus* L.) germplasm using agro-economic traits and microsatellite markers	Scientia Horticulturae	Article	0304-4238	Oct 2013	162	NA	278 284	10.1016/j.scienta.2013.08.029
275	Distefano, G et al.	Genetic diversity and relationships among Italian and foreign almond germplasm as revealed by microsatellite markers	Scientia Horticulturae	Article	0304-4238	Oct 2013	162	NA	305 312	10.1016/j.scienta.2013.08.030
276	Senan, S et al.	Novel polymorphic microsatellite markers from turmeric, *Curcuma longa* L. (Zingiberaceae)	Acta Botanica Croatica	Article	0365-0588	Oct 2013	72	2	407 412	10.2478/botcro-2013-0002
277	Kaewwongwal, A et al.	Genetic diversity and population structure of *Vigna exilis* and *Vigna grandiflora* (Phaseoleae, Fabaceae) from Thailand based on microsatellite variation	Botany-Botanique	Article	1916-2790	Oct 2013	91	10	653 661	10.1139/cjb-2013-0029
278	Izzah, NK et al.	Microsatellite-based analysis of genetic diversity in 91 commercial *Brassica oleracea* L. cultivars belonging to six varietal groups	Genetic Resources & Crop Evolution	Article	0925-9864	Oct 2013	60	7	1967 1986	10.1007/s10722-013-9966-3
279	Gavrilenko, T et al.	Genetic diversity and origin of cultivated potatoes based on plastid microsatellite polymorphism	Genetic Resources & Crop Evolution	Article	0925-9864	Oct 2013	60	7	1997 2015	10.1007/s10722-013-9968-1
280	Nantoume, AD et al.	Genetic differentiation of watermelon landrace types in Mali revealed by microsatellite (SSR) markers	Genetic Resources & Crop Evolution	Article	0925-9864	Oct 2013	60	7	2129 2141	10.1007/s10722-013-9980-5
281	Torres-Hernandez, S et al.	Genetic variability in *Malacomeles denticulata* (Rosaceae) from central Mexico revealed with SSR markers	Genetic Resources & Crop Evolution	Article	0925-9864	Oct 2013	60	7	2191 2200	10.1007/s10722-013-0041-x
282	Wasala, SK; Prasanna, BM	Microsatellite marker-based diversity and population genetic analysis of selected lowland and mid-altitude maize landrace accessions of India	Journal of Plant Biochemistry & Biotechnology	Article	0971-7811	Oct 2013	22	4	392 400	10.1007/s13562-012-0167-5
283	Bali, S et al.	Development of a set of genomic microsatellite markers in tea (*Camellia* L.) (Camelliaceae)	Molecular Breeding	Article	1380-3743	Oct 2013	32	3	735 741	10.1007/s11032-013-9902-4
284	Li, Y; Maki, M	Development of microsatellite markers for *Leucosceptrum japonicum* and *L. stellipilum* (Lamiaceae)	Applications in Plant Sciences	Article	2168-0450	Oct 2013	1	10	NA	10.3732/apps.1300038
285	Lopez-Roberts, MC et al.	Development of microsatellite markers in *Cratylia mollis* and their transferability to *C. argentea* (Fabaceae)	Applications in Plant Sciences	Article	2168-0450	Oct 2013	1	10	NA	10.3732/apps.1300015
286	Stingemore, JA et al.	Development of microsatellite markers for two Australian *Persoonia* (proteaceae) species using two different techniques	Applications in Plant Sciences	Article	2168-0450	Oct 2013	1	10	NA	10.3732/apps.1300023
287	Van Etten, ML et al.	Microsatellite markers for the New Zealand endemic tree *Fuchsia excorticata* (Onagraceae)	Applications in Plant Sciences	Article	2168-0450	Oct 2013	1	10	NA	10.3732/apps.1300045
288	Yamashiro, T et al.	Development of microsatellite markers for *Isodon longitubus* (Lamiaceae)	Applications in Plant Sciences	Article	2168-0450	Oct 2013	1	10	NA	10.3732/apps.1300028
289	Fang, M et al.	Development of microsatellite markers for *Croomia japonica* and cross-amplification in its congener	Scientia Horticulturae	Article	0304-4238	Sep 2013	161	NA	228 232	10.1016/j.scienta.2013.07.014
290	Gismondi, A; Canini, A	Microsatellite analysis of Latial *Olea europaea* L. cultivars	Plant Biosystems	Article	1126-3504	Sep 2013	147	3	686 691	10.1080/11263504.2012.751066
291	Ravishankar, KV et al.	Development and characterisation of microsatellite markers for wild banana (*Musa balbisiana*)	Journal of Horticultural Science & Biotechnology	Article	1462-0316	Sep 2013	88	5	605 609	NA
292	Radhika, V et al.	In silico identification and validation of microsatellite markers from onion EST sequences	Journal of Horticultural Science & Biotechnology	Article	1462-0316	Sep 2013	88	5	664 670	NA
293	Nemorin, A et al.	Microsatellite and flow cytometry analysis to help understand the origin of *Dioscorea alata* polyploids	Annals of Botany	Article	0305-7364	Sep 2013	112	5	811 819	10.1093/aob/mct145

	Author	Publication title	Source	Publication type	ISSN	Publication date	Vol	Issue	Pages	DOI
294	Jia, GQ et al.	Molecular diversity and population structure of Chinese green foxtail [*Setaria viridis* (L.) Beauv.] revealed by microsatellite analysis	Journal of Experimental Botany	Article	0022-0957	Sep 2013	64	12	3645 3655	10.1093/jxb/ert198
295	Angrizani, RC et al.	Development and characterization of microsatellite markers for the endangered amazonian tree *Aniba rosaeodora* (Lauraceae)	Applications in Plant Sciences	Article	2168-0450	Sep 2013	1	9	NA	10.3732/apps.1200516
296	Gaskin, JF et al.	Microsatellite markers for Russian olive (*Elaeagnus angustifolia*; Elaeagnaceae)	Applications in Plant Sciences	Article	2168-0450	Sep 2013	1	9	NA	10.3732/apps.1300013
297	Jennings, TN et al.	Microsatellite primers for the Pacific Northwest conifer *Callitropsis nootkatensis* (Cupressaceae)	Applications in Plant Sciences	Article	2168-0450	Sep 2013	1	9	NA	10.3732/apps.1300025
298	Ribeiro, DO et al.	Isolation of microsatellite markers for the red mangrove, *Rhizophora mangle* (Rhizophoraceae)	Applications in Plant Sciences	Article	2168-0450	Sep 2013	1	9	NA	10.3732/apps.1300003
299	Sakazono, S et al.	Development and characterization of microsatellite markers for *Lilium longiflorum* (Liliaceae)	Applications in Plant Sciences	Article	2168-0450	Sep 2013	1	9	NA	10.3732/apps.1300014
300	Wadl, PA et al.	Isolation and characterization of microsatellite loci for *Cornus sanguinea* (Cornaceae)	Applications in Plant Sciences	Article	2168-0450	Sep 2013	1	9	NA	10.3732/apps.1300012
301	Zhang, CM et al.	Development and characterization of microsatellite markers for sour jujube (*Ziziphus jujuba* var. *spinosa*)	Indian Journal of Genetics & Plant Breeding	Article	0019-5200	Aug 2013	73	3	338 341	10.5958/j.0975-6906.73.3.052
302	Ramu, P et al.	Assessment of genetic diversity in the sorghum reference set using EST-SSR markers	Theoretical & Applied Genetics	Article	0040-5752	Aug 2013	126	8	2051 2064	10.1007/s00122-013-2117-6
303	Kasthurirengan, S et al.	*In vitro* propagation and assessment of genetic stability of micropropagated *Samanea saman* (rain tree) using microsatellite markers	Acta Physiologiae Plantarum	Article	0137-5881	Aug 2013	35	8	2467 2474	10.1007/s11738-013-1281-2
304	Surapaneni, M et al.	Population structure and genetic analysis of different utility types of mango (*Mangifera indica* L.) germplasm of Andhra Pradesh state of India using microsatellite markers	Plant Systematics & Evolution	Article	0378-2697	Aug 2013	299	7	1215 1229	10.1007/s00606-013-0790-1
305	Sharma, PN et al.	Genetic diversity of two Indian common bean germplasm collections based on morphological and microsatellite markers	Plant Genetic Resources	Article	1479-2621	Aug 2013	11	2	121 130	10.1017/S1479262112000469
306	Culley, TM et al.	Development of 16 microsatellite markers within the *Camassia* (Agavaceae) species complex and amplification in related taxa	Applications in Plant Sciences	Article	2168-0450	Aug 2013	1	8	NA	10.3732/apps.1300001
307	Krohn, AL et al.	Microsatellite primers in the foundation tree species *Pinus edulis* and *P. monophylla* (Pinaceae)	Applications in Plant Sciences	Article	2168-0450	Aug 2013	1	8	NA	10.3732/apps.1200552
308	Liu, L et al.	Isolation and characterization of microsatellite markers in *Beilschmiedia roxburghiana* (Lauraceae)	Applications in Plant Sciences	Article	2168-0450	Aug 2013	1	8	NA	10.3732/apps.1200549
309	Matheny, H et al.	High-throughput microsatellite marker development for the distylous herb *Primula mistassinica* (Primulaceae)	Applications in Plant Sciences	Article	2168-0450	Aug 2013	1	8	NA	10.3732/apps.1300002
310	Shirk, RY et al.	Development and characterization of microsatellite primers in *Geranium carolinianum* (Geraniaceae) with 454 sequencing	Applications in Plant Sciences	Article	2168-0450	Aug 2013	1	8	NA	10.3732/apps.1300006
311	Xie, CX et al.	Development of the first chloroplast microsatellite loci in *Ginkgo biloba* (Ginkgoaceae)	Applications in Plant Sciences	Article	2168-0450	Aug 2013	1	8	NA	10.3732/apps.1300019
312	Pineda-Martos, R et al.	Genetic diversity of *Orobanche cumana* populations from Spain assessed using SSR markers	Weed Research	Article	0043-1737	Aug 2013	53	4	279 289	10.1111/wre.12022
313	Pardo, C et al.	Development of microsatellite loci for *Phymatolithon calcareum* (Corallinales, Rhodophyta) in the European Atlantic using NGS technology	Phycologia	Meeting Abstract	0031-8884	Aug 2013	52	4	85 85	NA
314	Chen, CM et al.	Isolation and characterization of 20 polymorphic microsatellite markers for *Juglans mandshurica* (Juglandaceae)	Applications in Plant Sciences	Article	2168-0450	Jul 2013	1	7	NA	10.3732/apps.1200009
315	Deletre, M et al.	Microsatellite markers for the yam bean *Pachyrhizus* (Fabaceae)	Applications in Plant Sciences	Article	2168-0450	Jul 2013	1	7	NA	10.3732/apps.1200551
316	Jain, N et al.	Discovery of est-derived microsatellite primers in the legume *Lens culinaris* (Fabaceae)	Applications in Plant Sciences	Article	2168-0450	Jul 2013	1	7	NA	10.3732/apps.1200539
317	Jiang, WX et al.	Development of polymorphic microsatellite markers for *Phyllostachys edulis* (Poaceae), an important bamboo species in China	Applications in Plant Sciences	Article	2168-0450	Jul 2013	1	7	NA	10.3732/apps.1200012
318	Ng, CH et al.	Isolation and characterization of microsatellite markers for *Shorea platyclados* (Dipterocarpaceae)	Applications in Plant Sciences	Article	2168-0450	Jul 2013	1	7	NA	10.3732/apps.1200538
319	Pan, Y et al.	Development of microsatellite markers in the oil-	Applications in Plant Sciences	Article	2168-0450	Jul 2013	1	7	NA	10.3732/apps.1200004

	Author	Publication title	Source	Publication type	ISSN	Publication date	Vol	Issue	Pages	DOI
		producing species *Vernicia fordii* (Euphorbiaceae), a potential biodiesel feedstock								
320	Witherup, C et al.	Development of microsatellite loci in *Artocarpus altilis* (Moraceae) and cross-amplification in congeneric species	Applications in Plant Sciences	Article	2168-0450	Jul 2013	1	7	NA	10.3732/apps.1200423
321	Gasi, F et al.	Assessment of European pear (*Pyrus communis* L.) Genetic resources in Bosnia and Herzegovina using microsatellite markers	Scientia Horticulturae	Article	0304-4238	Jun 2013	157	NA	74 83	10.1016/j.scienta.2013.04.017
322	Rodriguez, D et al.	Polymorphic microsatellite markers in pineapple (*Ananas comosus* (L.) Merrill)	Scientia Horticulturae	Article	0304-4238	Jun 2013	156	NA	127 130	10.1016/j.scienta.2013.03.026
323	Lavor, P et al.	Transferability of 10 nuclear microsatellite primers to *Vriesea minarum* (Bromeliaceae), a narrowly endemic and threatened species from Brazil	Brazilian Journal of Botany	Article	1806-9959	Jun 2013	36	2	165 168	10.1007/s40415-013-0012-7
324	Wang, MM et al.	Complexity of *indica-japonica* varietal differentiation in Bangladesh rice landraces revealed by microsatellite markers	Breeding Science	Editorial Material	1344-7610	Jun 2013	63	2	227 232	10.1270/jsbbs.63.227
325	Fang, DD et al.	A microsatellite-based genome-wide analysis of genetic diversity and linkage disequilibrium in upland cotton (*Gossypium hirsutum* L.) cultivars from major cotton-growing countries	Euphytica	Article	0014-2336	Jun 2013	191	3	391 401	10.1007/s10681-013-0886-2
326	Yu, JY et al.	Transferability of rice SSR markers to *Miscanthus sinensis*, a potential biofuel crop	Euphytica	Article	0014-2336	Jun 2013	191	3	455 468	10.1007/s10681-013-0915-1
327	Zeng, WF et al.	Microsatellite polymorphism is likely involved in phytoene synthase activity in *Citrus*	Plant Cell Tissue & Organ Culture	Article	0167-6857	Jun 2013	113	3	449 458	10.1007/s11240-012-0285-8
328	Wei, ZZ et al.	Genetic diversity and population structure in Chinese indigenous poplar (*Populus simonii*) populations using microsatellite markers	Plant Molecular Biology Reporter	Article	0735-9640	Jun 2013	31	3	620 632	10.1007/s11105-012-0527-2
329	Duan, YF et al.	Genetic diversity of androdioecious *Osmanthus fragrans* (Oleaceae) cultivars using microsatellite markers	Applications in Plant Sciences	Article	2168-0450	Jun 2013	1	6	NA	10.3732/apps.1200092
330	Falahati-Anbaran, M et al.	Development of microsatellite markers for the neotropical vine *Dalechampia scandens* (Euphorbiaceae)	Applications in Plant Sciences	Article	2168-0450	Jun 2013	1	6	NA	10.3732/apps.1200492
331	Fan, DM et al.	Development of microsatellite loci for *Cyclocarya paliurus* (Juglandaceae), a monotypic species in subtropical China	Applications in Plant Sciences	Article	2168-0450	Jun 2013	1	6	NA	10.3732/apps.1200524
332	Fant, JB et al.	Characterization of microsatellite loci in *Castilleja sessiliflora* and transferability to 24 *Castilleja* species (Orobanchaceae)	Applications in Plant Sciences	Article	2168-0450	Jun 2013	1	6	NA	10.3732/apps.1200564
333	Mansour, H et al.	Development of 13 microsatellite markers in the endangered sinai primrose (*Primula boveana*, Primulaceae)	Applications in Plant Sciences	Article	2168-0450	Jun 2013	1	6	NA	10.3732/apps.1200515
334	Martins, AR et al.	Development and characterization of microsatellite markers for the medicinal plant *Smilax brasiliensis* (Smilacaceae) and related species	Applications in Plant Sciences	Article	2168-0450	Jun 2013	1	6	NA	10.3732/apps.1200507
335	Tambarussi, EV et al.	Microsatellite markers for *Cariniana legalis* (Lecythidaceae) and their transferability to *C. estrellensis*	Applications in Plant Sciences	Article	2168-0450	Jun 2013	1	6	NA	10.3732/apps.1200493
336	Li, H; Geng, SL	Development and characterization of microsatellite markers for *Derris elliptica* (Fabaceae), an insecticide-producing plant	Scientia Horticulturae	Article	0304-4238	May 2013	154	NA	54 60	10.1016/j.scienta.2013.02.026
337	Sohrabi, M et al.	Genetic divergence of Malaysian upland rices revealed by microsatellite markers	Plant Omics	Article	1836-0661	May 2013	6	3	175 182	NA
338	Xiao, Y et al.	Development of microsatellite markers in *Cocos nucifera* and their application in evaluating the level of genetic diversity of *Cocos nucifera*	Plant Omics	Article	1836-0661	May 2013	6	3	193 200	NA
339	Behera, L et al.	Assessment of genetic diversity of rainfed lowland rice genotypes using microsatellite markers	Indian Journal of Genetics & Plant Breeding	Article	0019-5200	May 2013	73	2	142 152	10.5958/j.0975-6906.73.2.021
340	Jiang, X et al.	Distinguishing morphologically similar *Zostera species* (*Z. caespitosa* and *Z. marina*) using microsatellite DNA markers on leaf fragments	Aquatic Botany	Article	0304-3770	May 2013	107	NA	59 62	10.1016/j.aquabot.2013.01.006
341	Mattioni, C et al.	Microsatellite markers reveal a strong geographical structure in European populations of *Castanea sativa*	American Journal of Botany	Article	0002-9122	May 2013	100	5	951 961	10.3732/ajb.1200194

	Author	Publication title	Source	Publication type	ISSN	Publication date	Vol	Issue	Pages	DOI
		(Fagaceae): evidence for multiple glacial refugia								
342	Wende, A et al.	Genetic interrelationships among medium to late maturing tropical maize inbred lines using selected SSR markers	Euphytica	Article	0014-2336	May 2013	191	2	269 277	10.1007/s10681-012-0826-6
343	Albertse, EH; Joshi, SV	Microsatellite DNA fingerprinting and cultivar identification in sugarcane using a semi-automated genetic analyser	South African Journal of Botany	Meeting Abstract	0254-6299	May 2013	86	NA	171 171	10.1016/j.sajb.2013.02.123
344	Bessega, CF et al.	New microsatellite loci for *Prosopis alba* and *P. chilensis* (Fabaceae)	Applications in Plant Sciences	Article	2168-0450	May 2013	1	5	NA	10.3732/apps.1200324
345	Deng, Q et al.	Microsatellite loci for an old rare species, *Pseudotaxus chienii*, and transferability in *Taxus wallichiana* var. *mairei* (Taxaceae)	Applications in Plant Sciences	Article	2168-0450	May 2013	1	5	NA	10.3732/apps.1200456
346	Ishibashi, CDA et al.	Isolation of microsatellite markers in a chaparral species endemic to Southern California, *Ceanothus megacarpus* (Rhamnaceae)	Applications in Plant Sciences	Article	2168-0450	May 2013	1	5	NA	10.3732/apps.1200393
347	Nevill, PG et al.	Microsatellite primers identified by 454 sequencing in the floodplain tree species *Eucalyptus victrix* (Myrtaceae)	Applications in Plant Sciences	Article	2168-0450	May 2013	1	5	NA	10.3732/apps.1200402
348	Nevill, PG et al.	Development of microsatellite loci for the riparian tree species *Melaleuca argentea* (Myrtaceae) using 454 sequencing	Applications in Plant Sciences	Article	2168-0450	May 2013	1	5	NA	10.3732/apps.1200401
349	Oguri, E et al.	Microsatellite markers for *Leucobryum boninense* (Leucobryaceae), endemic to the bonin islands, japan	Applications in Plant Sciences	Article	2168-0450	May 2013	1	5	NA	10.3732/apps.1200399
350	Ohki, N; Setoguchi, H	New microsatellite markers for *Tricyrtis macrantha* (Convallariaceae) and cross-amplification in closely related species	Applications in Plant Sciences	Article	2168-0450	May 2013	1	5	NA	10.3732/apps.1200247
351	Twyford, AD et al.	Development and characterization of microsatellite markers for central American *Begonia* sect. *Gireoudia* (Begoniaceae)	Applications in Plant Sciences	Article	2168-0450	May 2013	1	5	NA	10.3732/apps.1200499
352	McCleary, T et al.	EST-SSR markers reveal synonymies, homonymies and relationships inconsistent with putative pedigrees in chestnut cultivars	Genetic Resources & Crop Evolution	Article	0925-9864	Apr 2013	60	4	1209 1222	10.1007/s10722-012-9912-9
353	Manca, A et al.	Evaluation of genetic diversity in a *Camelina sativa* (L.) Crantz collection using microsatellite markers and biochemical traits	Genetic Resources & Crop Evolution	Article	0925-9864	Apr 2013	60	4	1223 1236	10.1007/s10722-012-9913-8
354	Adugna, A et al.	Population genetic structure of in situ wild *Sorghum bicolor* in its Ethiopian center of origin based on SSR markers	Genetic Resources & Crop Evolution	Article	0925-9864	Apr 2013	60	4	1313 1328	10.1007/s10722-012-9921-8
355	Li, FG et al.	Generation and analysis of expressed sequence tags for microsatellite marker development in *Calamus simplicifolius* C. F. Wei	Molecular Breeding	Article	1380-3743	Apr 2013	31	4	867 877	10.1007/s11032-013-9840-1
356	Li, HT et al.	Development of a core set of single-locus SSR markers for allotetraploid rapeseed (*Brassica napus* L.)	Theoretical & Applied Genetics	Article	0040-5752	Apr 2013	126	4	937 947	10.1007/s00122-012-2027-z
357	Caddah, MK et al.	Species boundaries inferred from microsatellite markers in the *Kielmeyera coriacea* complex (Calophyllaceae) and evidence of asymmetric hybridization	Plant Systematics & Evolution	Article	0378-2697	Apr 2013	299	4	731 741	10.1007/s00606-012-0755-9
358	Madhou, M et al.	Fingerprinting and analysis of genetic diversity of litchi (*Litchi chinensis* Sonn.) accessions from different germplasm collections using microsatellite markers	Tree Genetics & Genomes	Article	1614-2942	Apr 2013	9	2	387 396	10.1007/s11295-012-0560-1
359	Phuekvilai, P; Wolff, K	Characterization of microsatellite loci in *Tilia platyphyllos* (Malvaceae) and cross-amplification in related species	Applications in Plant Sciences	Article	2168-0450	Apr 2013	1	4	NA	10.3732/apps.1200386
360	Ross, AA et al.	Microsatellite markers in the western prairie fringed orchid, *Platanthera praeclara* (Orchidaceae)	Applications in Plant Sciences	Article	2168-0450	Apr 2013	1	4	NA	10.3732/apps.1200413
361	Sakata, Y et al.	Isolation and characterization of microsatellite loci in the invasive herb *Solidago altissima* (Asteraceae)	Applications in Plant Sciences	Article	2168-0450	Apr 2013	1	4	NA	10.3732/apps.1200313
362	Wei, N et al.	Polymorphic microsatellite loci for *Virola sebifera* (Myristicaceae) derived from shotgun 454 pyrosequencing	Applications in Plant Sciences	Article	2168-0450	Apr 2013	1	4	NA	10.3732/apps.1200295
363	Zou, Y et al.	Development and characterization of microsatellite	Applications in Plant Sciences	Article	2168-0450	Apr 2013	1	4	NA	10.3732/apps.1200457

	Author	Publication title	Source	Publication type	ISSN	Publication date	Vol	Issue	Pages	DOI
		markers for *Alpinia oxyphylla* (Zingiberaceae)								
364	Fajardo, D et al.	Discrimination of American cranberry cultivars and assessment of clonal heterogeneity using microsatellite markers	Plant Molecular Biology Reporter	Article	0735-9640	Apr 2013	31	2	264 271	10.1007/s11105-012-0497-4
365	Emanuelli, F et al.	Genetic diversity and population structure assessed by SSR and SNP markers in a large germplasm collection of grape	BMC Plant Biology	Article	1471-2229	Mar 2013	13	NA	NA	10.1186/1471-2229-13-39
366	Ganeva, G et al.	Frost tolerance in winter wheat cultivars: different effects of chromosome 5A and association with microsatellite alleles	Biologia Plantarum	Article	0006-3134	Mar 2013	57	1	184 188	10.1007/s10535-012-0267-z
367	Zamani-Nour, S et al.	Cytoplasmic diversity of *Brassica napus* L., *Brassica oleracea* L. and *Brassica rapa* L. as determined by chloroplast microsatellite markers	Genetic Resources & Crop Evolution	Article	0925-9864	Mar 2013	60	3	953 965	10.1007/s10722-012-9891-x
368	Cabral, AL et al.	The use of microsatellite polymorphisms to characterise and compare genetic variability in *Avena strigosa* and *A. barbata*	Genetic Resources & Crop Evolution	Article	0925-9864	Mar 2013	60	3	1153 1163	10.1007/s10722-012-9911-x
369	Leonardia, AAP et al.	Population genetic structure of the tropical moss *Acanthorrhynchium papillatum* as measured with microsatellite markers	Plant Biology	Article	1435-8603	Mar 2013	15	2	384 394	10.1111/j.1438-8677.2012.00640.x
370	Dal Grande, et al.	Microsatellite primers in the lichen symbiotic alga *Trebouxia decolorans* (Trebouxiophyceae)	Applications in Plant Sciences	Article	2168-0450	Mar 2013	1	3	NA	10.3732/apps.1200400
371	Gajurel, JP et al.	Development and characterization of microsatellite loci in the endangered species *Taxus wallichiana* (Taxaceae)	Applications in Plant Sciences	Article	2168-0450	Mar 2013	1	3	NA	10.3732/apps.1200281
372	Liu, HB et al.	Development and characterization of microsatellite markers in *Prunus sibirica* (Rosaceae)	Applications in Plant Sciences	Article	2168-0450	Mar 2013	1	3	NA	10.3732/apps.1200074
373	Liu, T et al.	Development and characterization of microsatellite markers for *Melastoma dodecandrum* (Melastomataceae)	Applications in Plant Sciences	Article	2168-0450	Mar 2013	1	3	NA	10.3732/apps.1200294
374	Pereira, MF et al.	Shotgun sequencing for microsatellite identification in *Ilex paraguariensis* (Aquifoliaceae)	Applications in Plant Sciences	Article	2168-0450	Mar 2013	1	3	NA	10.3732/apps.1200245
375	Yu, JH et al.	Development and characterization of polymorphic microsatellite loci in *Phellodendron amurense* (Rutaceae)	Applications in Plant Sciences	Article	2168-0450	Mar 2013	1	3	NA	10.3732/apps.1200321
376	Mengesha, WA et al.	Genetic diversity and population structure of Guinea yams and their wild relatives in South and South West Ethiopia as revealed by microsatellite markers	Genetic Resources & Crop Evolution	Article	0925-9864	Feb 2013	60	2	529 541	10.1007/s10722-012-9856-0
377	Bassil, N et al.	Nuclear and chloroplast microsatellite markers to assess genetic diversity and evolution in hazelnut species, hybrids and cultivars	Genetic Resources & Crop Evolution	Article	0925-9864	Feb 2013	60	2	543 568	10.1007/s10722-012-9857-z
378	Wang, JY; Chuang, KC	Development of novel microsatellite markers for effective applications in *Anthurium* cultivar identification	Euphytica	Article	0014-2336	Feb 2013	189	3	421 431	10.1007/s10681-012-0799-5
379	Sullivan, AR et al.	Development and characterization of genomic and gene-based microsatellite markers in North American red oak species	Plant Molecular Biology Reporter	Article	0735-9640	Feb 2013	31	1	231 239	10.1007/s11105-012-0495-6
380	Assoumane, A et al.	Highlighting the occurrence of tetraploidy in *Acacia senegal* (L.) Willd. and genetic variation patterns in its natural range revealed by DNA microsatellite markers	Tree Genetics & Genomes	Article	1614-2942	Feb 2013	9	1	93 106	10.1007/s11295-012-0537-0
381	Fatemi, M et al.	Cost-effective microsatellite markers for *Banksia integrifolia* (Proteaceae)	Applications in Plant Sciences	Article	2168-0450	Feb 2013	1	2	NA	10.3732/apps.1200130
382	Liu, Y et al.	Development of microsatellite markers for *Lagerstroemia indica* (Lythraceae) and related species	Applications in Plant Sciences	Article	2168-0450	Feb 2013	1	2	NA	10.3732/apps.1200203
383	Stojanova, B	Isolation and characterization of microsatellite markers for the cleistogamous species *Lamium amplexicaule* (Lamiaceae)	Applications in Plant Sciences	Article	2168-0450	Feb 2013	1	2	NA	10.3732/apps.1200259
384	Wu, ZG et al.	Development of microsatellite markers in the hexaploid aquatic macrophyte, *Myriophyllum spicatum* (Haloragaceae)	Applications in Plant Sciences	Article	2168-0450	Feb 2013	1	2	NA	10.3732/apps.1200230
385	Rauscher, G; Simko, I	Development of genomic SSR markers for fingerprinting lettuce (*Lactuca sativa* L.) cultivars and mapping genes	BMC Plant Biology	Article	1471-2229	Jan 2013	13	NA	NA	10.1186/1471-2229-13-11
386	Guo, WX et al.	Development and characterization of microsatellite	Applications in Plant Sciences	Article	2168-0450	Jan 2013	1	1	NA	10.3732/apps.1200211

	Author	Publication title	Source	Publication type	ISSN	Publication date	Vol	Issue	Pages	DOI
		loci for smooth cordgrass, *Spartina alterniflora* (Poaceae)								
387	Tang, AJ et al.	Development of 11 microsatellite loci for an endangered herb, *Paraisometrum mileense* (Gesneriaceae), in Southwest China	Applications in Plant Sciences	Article	2168-0450	Jan 2013	1	1	NA	10.3732/apps.1200133
388	Yamashiro, A et al.	Isolation and characterization of microsatellite markers for *Canavalia cathartica* and C. *lineata* (Fabaceae)	Applications in Plant Sciences	Article	2168-0450	Jan 2013	1	1	NA	10.3732/apps.1200111
389	Zhang, L et al.	Isolation and characterization of 27 microsatellite markers for the endemic species *Diplarche multiflora* (Ericaceae)	Applications in Plant Sciences	Article	2168-0450	Jan 2013	1	1	NA	10.3732/apps.1200235
390	Zhou, HF et al.	Isolation and characterization of microsatellite loci for a bioenergy grass, *Miscanthus sacchariflorus* (Poaceae)	Applications in Plant Sciences	Article	2168-0450	Jan 2013	1	1	NA	10.3732/apps.1200210
391	Kimura, MK et al.	Isolation and characterization of chloroplast microsatellite markers in the invasive tree species *Robinia pseudoacacia* L.	Silvae Genetica	Article	0037-5349	NA 2013	62	4-5	207 209	NA
392	Shepherd, M et al.	Microsatellite markers for *Eucalyptus pilularis* (Subgenus *Eucalyptus*); sourcing genetic markers outside the subgenus	Silvae Genetica	Article	0037-5349	NA 2013	62	4-5	246 255	NA
393	Moraes, RCS et al.	Microsatellite markers for an endemic Atlantic Forest tree, *Manilkara multifida* (Sapotaceae)	AoB Plants	Article	2041-2851	NA 2013	5	NA	NA	10.1093/aobpla/plt006
394	Chessa, I et al.	Polymorphic microsatellite DNA markers in *Opuntia* spp. collections	VII International Congress on Cactus Pear and Cochinealse, Acta Horticulturae	Proceedings Paper	0567-7572BN 978-90-66055-36-0	Oct 2013	995	NA	35 41	NA
395	Guo, DL et al.	Characterization of grape cultivars from China using microsatellite markers	Czech Journal of Genetics & Plant Breeding	Article	1212-1975	NA 2013	49	4	164 170	NA
396	Niu, HY et al.	Short Note: Development and characterization of 16 new polymorphic microsatellite loci for *Schima superba* (Theaceae)	Silvae Genetica	Article	0037-5349	NA 2013	62	3	124 127	NA
397	Milner, ML et al.	Microsatellite variation for phylogenetic, phylogeographic and population-genetic studies in *Lomatia* (Proteaceae)	Australian Systematic Botany	Article	1030-1887	NA 2013	26	3	186 195	10.1071/SB13002
398	Musilova, M et al.	Genetic variability for coloured caryopses in common wheat varieties determined by microsatellite markers	Czech Journal of Genetics & Plant Breeding	Article	1212-1975	NA 2013	49	3	116 122	NA
399	Danner, MA et al.	Mendelian segregation in eight microsatellite loci from hand- and open-pollinated progenies of *Araucaria angustifolia* (Bert.) O. Kuntze (Araucariaceae)	Silvae Genetica	Article	0037-5349	NA 2013	62	1-2	18 25	NA
400	Zhang, J et al.	The diploid origins of allopolyploid rose species studied using single nucleotide polymorphism haplotypes flanking a microsatellite repeat	Journal of Horticultural Science & Biotechnology	Article	1462-0316	Jan 2013	88	1	85 92	NA
401	Lendvay, B et al.	Characterization of nuclear microsatellite markers for the narrow endemic *Syringa josikaea* Jacq. fil. ex Rchb.	Notulae Botanicae Horti Agrobotanici Cluj-Napoca	Article	0255-965X	Jan-Jun 2013	41	1	301 305	NA
402	Hamwieh, A et al.	Genetic diversity of microsatellite alleles located at quantitative resistance loci for Ascochyta blight resistance in a global collection of chickpea germplasm	Phytopathologia Mediterranea	Article	0031-9465	NA 2013	52	1	183 191	NA
403	Li, WJ et al.	Microsatellite DNA markers indicate quantitative trait loci controlling resistance to pea root rot caused by *Fusarium avenaceum*	Canadian Journal of Plant Pathology	Meeting Abstract	0706-0661	Jan 2013	35	1	117 117	NA
404	Shah, SM et al.	Genetic diversity in Basmati and non-Basmati rice varieties based on microsatellite markers	Pakistan Journal of Botany	Article	0556-3321	Jan 2013	45	NA	423 431	NA
405	Wee, AKS et al.	Microsatellite loci for *Avicennia alba* (Acanthaceae), *Sonneratia alba* (Lythraceae) and *Rhizophora mucronata* (Rhizophoraceae)	Journal of Tropical Forest Science	Article	0128-1283	Jan 2013	25	1	131 136	NA
406	Gasi, F et al.	Evaluation of apple (*Malus* x *domestica*) genetic resources in Bosnia and Herzegovina using microsatellite markers	Hortscience	Article	0018-5345	Jan 2013	48	1	13 21	NA
407	Gilmore, B et al.	Microsatellite marker development in peony using next generation sequencing	Journal of the American Society for Horticultural Science	Article	0003-1062	Jan 2013	138	1	64 74	NA
408	Rodriguez, E et al.	Pb^{2+} exposure induced microsatellite instability in	Plant Physiology &	Article	0981-9428	Jan 2013	62	NA	19 22	10.1016/j.plaphy.2012.10.006

	Author	Publication title	Source	Publication type	ISSN	Publication date		Vol	Issue	Pages		DOI
		Pisum sativum in a locus related with glutamine metabolism	Biochemistry									
409	Jaikishan, I et al.	Development of microsatellite markers targeting (GATA) (n) motifs in sorghum [*Sorghum bicolor* (L.) Moench]	Molecular Breeding	Article	1380-3743	Jan	2013	31	1	223	231	10.1007/s11032-012-9770-3
410	Twyford, AD et al.	Population history and seed dispersal in widespread Central American *Begonia* species (Begoniaceae) inferred from plastome-derived microsatellite markers	Botanical Journal of the Linnean Society	Article	0024-4074	Jan	2013	171	1	260	276	10.1111/j.1095-8339.2012.01265.x
411	Lund, B et al.	Detection of duplicates among repatriated Nordic spring barley (*Hordeum vulgare* L. s.l.) accessions using agronomic and morphological descriptors and microsatellite markers	Genetic Resources & Crop Evolution	Article	0925-9864	Jan	2013	60	1	1	11	10.1007/s10722-012-9809-7
412	Moretzsohn, MC et al.	A study of the relationships of cultivated peanut (*Arachis hypogaea*) and its most closely related wild species using intron sequences and microsatellite markers	Annals of Botany	Article	0305-7364	Jan	2013	111	1	113	126	10.1093/aob/mcs237
413	Zehdi, S et al.	Molecular polymorphism and genetic relationships in date palm (*Phoenix dactylifera* L.): The utility of nuclear microsatellite markers	Scientia Horticulturae	Article	0304-4238	Dec	2012	148	NA	255	263	10.1016/j.scienta.2012.10.011
414	Singh, V et al.	Genetic analysis of Basmati RILs and characterization of major fragrance gene by microsatellite markers	Vegetos	Article	0970-4078	Dec	2012	25	2	266	272	NA
415	Abe, H et al.	Isolation and characterization of microsatellite loci in a polyploid alpine herb, *Callianthemum miyabeanum* (Ranunculaceae)	American Journal of Botany	Article	0002-9122	Dec	2012	99	12	E484	E486	10.3732/ajb.1200202
416	Chen, JL et al.	Microsatellite markers for *Kleinia neriifolia*, an endemic Asteraceae species on the Canary Islands	American Journal of Botany	Article	0002-9122	Dec	2012	99	12	E474	E476	10.3732/ajb.1200166
417	Ramos, SLF et al.	Microsatellite loci for tucuma of Amazonas (*Astrocaryum aculeatum*) and amplification in other Arecaceae	American Journal of Botany	Article	0002-9122	Dec	2012	99	12	E508	E510	10.3732/ajb.1100607
418	Gao, LM et al.	Microsatellite markers developed for *Corallodiscus lanuginosus* (Gesneriaceae) and their cross-species transferability	American Journal of Botany	Article	0002-9122	Dec	2012	99	12	E490	E492	10.3732/ajb.1200178
419	Krapp, F et al.	A set of plastid microsatellite loci for the genus *Dyckia* (Bromeliaceae) derived from 454 pyrosequencing	American Journal of Botany	Article	0002-9122	Dec	2012	99	12	E470	E473	10.3732/ajb.1200153
420	Li, C et al.	Development of microsatellite markers for the endangered medicinal plant *Launaea arborescens* (Asteraceae)	American Journal of Botany	Article	0002-9122	Dec	2012	99	12	E481	E483	10.3732/ajb.1200126
421	Nunziata, SO et al.	Characterization of 42 polymorphic microsatellite loci in *Mimulus ringens* (Phrymaceae) using Illumina sequencing	American Journal of Botany	Article	0002-9122	Dec	2012	99	12	E477	E480	10.3732/ajb.1200180
422	Takayama, K et al.	Development of microsatellite markers in species of *Erigeron* (Asteraceae) endemic to the Juan Fernandez Archipelago, Chile	American Journal of Botany	Article	0002-9122	Dec	2012	99	12	E487	E489	10.3732/ajb.1200218
423	Sun, JC et al.	Comparative genetic structure within single-origin pairs of rice (*Oryza sativa* L.) landraces from *in situ* and *ex situ* conservation programs in Yunnan of China using microsatellite markers	Genetic Resources & Crop Evolution	Article	0925-9864	Dec	2012	59	8	1611	1623	10.1007/s10722-011-9786-2
424	Grover, A et al.	Random genomic scans at microsatellite loci for genetic diversity estimation in cold-adapted *Lepidium latifolium*	Plant Genetic Resources	Article	1479-2621	Dec	2012	10	3	224	231	10.1017/S1479262112000299
425	Urrestarazu, J et al.	Genetic diversity and structure of local apple cultivars from Northeastern Spain assessed by microsatellite markers	Tree Genetics & Genomes	Article	1614-2942	Dec	2012	8	6	1163	1180	10.1007/s11295-012-0502-y
426	Nemorin, A et al.	Inheritance pattern of tetraploid *Dioscorea alata* and evidence of double reduction using microsatellite marker segregation analysis	Molecular Breeding	Article	1380-3743	Dec	2012	30	4	1657	1667	10.1007/s11032-012-9749-0
427	Bansal, UK et al.	Microsatellite mapping identifies TTKST-effective stem rust resistance gene in wheat cultivars VL404 and Janz	Molecular Breeding	Article	1380-3743	Dec	2012	30	4	1757	1765	10.1007/s11032-012-9759-y
428	Yumnam, JS et al.	Evaluation of genetic diversity of chilli landraces from North Eastern India based on morphology, SSR markers and the *Pun1* locus	Plant Molecular Biology Reporter	Article	0735-9640	Dec	2012	30	6	1470	1479	10.1007/s11105-012-0466-y

	Author	Publication title	Source	Publication type	ISSN	Publication date	Vol	Issue	Pages	DOI
429	Santos, ESL et al.	New polymorphic microsatellite loci for *Theobroma cacao*: isolation and characterization of microsatellites from enriched genomic libraries	Biologia Plantarum	Article	0006-3134	Dec 2012	56	4	789 792	10.1007/s10535-012-0134-y
430	Kumari, M et al.	Identification of microsatellite markers associated with staygreen trait in wheat RILs	Indian Journal of Genetics & Plant Breeding	Article	0019-5200	Nov 2012	72	4	415 420	NA
431	Sandhu, N et al.	Microsatellite diversity among aerobic and lowland *indica* rice genotypes with differential water requirements	Indian Journal of Genetics & Plant Breeding	Article	0019-5200	Nov 2012	72	4	463 467	NA
432	Gupta, SK et al.	Development of EST-derived microsatellite markers in mungbean [*Vigna radiata* (L.) Wilczek] and their transferability to other *Vigna* species	Indian Journal of Genetics & Plant Breeding	Article	0019-5200	Nov 2012	72	4	468 471	NA
433	Rehman, AU et al.	Microsatellite marker-based identification of mother plants for the reliable propagation of olive (*Olea europaea* L.) cultivars in Australia	Journal of Horticultural Science & Biotechnology	Article	1462-0316	Nov 2012	87	6	647 653	NA
434	Brzyski, JR et al.	Characterization of 12 polymorphic microsatellite markers in the liverwort *Marchantia inflexa* (Marchantiaceae)	American Journal of Botany	Article	0002-9122	Nov 2012	99	11	E440 E442	10.3732/ajb.1200187
435	Chiang, YC et al.	Characterization of 21 microsatellite markers from cogongrass, *Imperata cylindrica* (Poaceae), a weed species distributed worldwide	American Journal of Botany	Article	0002-9122	Nov 2012	99	11	E428 E430	10.3732/ajb.1200152
436	Crichton, RJ et al.	Isolation of microsatellite primers for *Melampyrum sylvaticum* (Orobanchaceae), an endangered plant in the United Kingdom	American Journal of Botany	Article	0002-9122	Nov 2012	99	11	E457 E459	10.3732/ajb.1200103
437	Duan, TT et al.	Development of microsatellite markers from *Mussaenda pubescens* (Rubiaceae)	American Journal of Botany	Article	0002-9122	Nov 2012	99	11	E437 E439	10.3732/ajb.1200188
438	Ju, LP et al.	Microsatellite primers for the endangered beech tree, *Fagus hayatae* (Fagaceae)	American Journal of Botany	Article	0002-9122	Nov 2012	99	11	E453 E456	10.3732/ajb.1200118
439	Kartzinel, TR et al.	Microsatellite primers for the neotropical epiphyte *Epidendrum firmum* (Orchidaceae)	American Journal of Botany	Article	0002-9122	Nov 2012	99	11	E450 E452	10.3732/ajb.1200232
440	Matter, P et al.	Eleven microsatellite markers for the mountain clover *Trifolium montanum* (Fabaceae)	American Journal of Botany	Article	0002-9122	Nov 2012	99	11	E447 E449	10.3732/ajb.1200102
441	Misiewicz, TM et al.	Microsatellite primers for an Amazonian lowland tropical tree, *Protium subserratum* (Burseraceae)	American Journal of Botany	Article	0002-9122	Nov 2012	99	11	E465 E467	10.3732/ajb.1200229
442	Morgante, PG et al.	Development of microsatellite markers for pimenta *Pseudocaryophyllus* (Myrtaceae), a wild South American species	American Journal of Botany	Article	0002-9122	Nov 2012	99	11	E434 E436	10.3732/ajb.1200167
443	Tnah, LH et al.	Isolation and characterization of microsatellite markers for an important tropical tree, *Aquilaria malaccensis* (Thymelaeaceae)	American Journal of Botany	Article	0002-9122	Nov 2012	99	11	E431 E433	10.3732/ajb.1200165
444	Ahmad, S et al.	Assessment of genetic diversity in 35 *Pisum sativum* accessions using microsatellite markers	Canadian Journal of Plant Science	Article	0008-4220	Nov 2012	92	6	1075 1081	10.4141/CJPS2011-261
445	Diekmann, K et al.	New chloroplast microsatellite markers suitable for assessing genetic diversity of *Lolium perenne* and other related grass species	Annals of Botany	Article	0305-7364	Nov 2012	110	6	1327 1339	10.1093/aob/mcs044
446	Zhang, DL et al.	Identifying loci influencing grain number by microsatellite screening in bread wheat (*Triticum aestivum* L.)	Planta	Article	0032-0935	Nov 2012	236	5	1507 1517	10.1007/s00425-012-1708-9
447	Wang, HX et al.	Development and cross-species/genera transferability of microsatellite markers discovered using 454 genome sequencing in chokecherry (*Prunus virginiana* L.)	Plant Cell Reports	Article	0721-7714	Nov 2012	31	11	2047 2055	10.1007/s00299-012-1315-z
448	Alamuti, MK	Extensive genetic diversity in Iranian pomegranate (*Punica granatum* L.) germplasm revealed by microsatellite markers	Scientia Horticulturae	Article	0304-4238	Oct 2012	146	NA	104 114	10.1016/j.scienta.2012.07.029
449	Matter, P et al.	Eight microsatellite markers for the bulbous buttercup *Ranunculus bulbosus* (Ranunculaceae)	American Journal of Botany	Article	0002-9122	Oct 2012	99	10	E399 E401	10.3732/ajb.1200101
450	Oliveira, FA et al.	Microsatellite markers for *Plathymenia reticulata* (Leguminosae)	American Journal of Botany	Article	0002-9122	Oct 2012	99	10	E391 E393	10.3732/ajb.1200051
451	Poncet, V et al.	Microsatellite markers for *Amborella* (Amborellaceae), a monotypic genus endemic to New Caledonia	American Journal of Botany	Article	0002-9122	Oct 2012	99	10	E411 E414	10.3732/ajb.1200131
452	Su, HL et al.	Isolation and characterization of polymorphic microsatellite loci in the endangered plant *Dipteronia*	American Journal of Botany	Article	0002-9122	Oct 2012	99	10	E425 E427	10.3732/ajb.1200151

	Author	Publication title	Source	Publication type	ISSN	Publication date		Vol	Issue	Pages		DOI
		sinensis (Sapindaceae)										
453	Tacuatia, LO et al.	Development and characterization of nine microsatellite loci for *Sisyrinchium micranthum* (Iridaceae)	American Journal of Botany	Article	0002-9122	Oct	2012	99	10	E402	E404	10.3732/ajb.1200105
454	Wang, LH et al.	Development and characterization of 59 polymorphic cDNA-SSR markers for the edible oil crop *Sesamum indicum* (Pedaliaceae)	American Journal of Botany	Article	0002-9122	Oct	2012	99	10	E394	E398	10.3732/ajb.1200081
455	Yu, JH et al.	Isolation and characterization of 13 novel polymorphic microsatellite markers for *Pinus koraiensis* (Pinaceae)	American Journal of Botany	Article	0002-9122	Oct	2012	99	10	E421	E424	10.3732/ajb.1200145
456	Tong, et al.	Large-scale development of microsatellite markers in *Nicotiana tabacum* and construction of a genetic map of flue-cured tobacco	Plant Breeding	Article	0179-9541	Oct	2012	131	5	674	680	10.1111/j.1439-0523.2012.01984.x
457	Blair, MW et al.	First use of microsatellite markers in a large collection of cultivated and wild accessions of tepary bean (*Phaseolus acutifolius* A. Gray)	Theoretical & Applied Genetics	Article	0040-5752	Oct	2012	125	6	1137	1147	10.1007/s00122-012-1900-0
458	Hou, BW et al.	Genetic diversity assessment and *ex situ* conservation strategy of the endangered *Dendrobium officinale* (Orchidaceae) using new trinucleotide microsatellite markers	Plant Systematics & Evolution	Article	0378-2697	Oct	2012	298	8	1483	1491	10.1007/s00606-012-0651-3
459	Upadhyaya, HD et al.	SSR markers linked to kernel weight and tiller number in *Sorghum* identified by association mapping	Euphytica	Article	0014-2336	Oct	2012	187	3	401	410	10.1007/s10681-012-0726-9
460	Giovannini, D et al.	Assessment of genetic variability in Italian heritage peach resources from Emilia-Romagna using microsatellite markers	Journal of Horticultural Science & Biotechnology	Article	1462-0316	Sep	2012	87	5	435	440	NA
461	Barrandeguy, ME et al.	Development of microsatellite markers for *Anadenanthera colubrina* var. *cebil* (Fabaceae), a native tree from South America	American Journal of Botany	Article	0002-9122	Sep	2012	99	9	E372	E374	10.3732/ajb.1200078
462	Clivati, D et al.	Microsatellite markers developed for *Utricularia reniformis* (Lentibulariaceae)	American Journal of Botany	Article	0002-9122	Sep	2012	99	9	E375	E378	10.3732/ajb.1200080
463	Dong, YR et al.	Sixteen novel microsatellite markers developed for *Dendrocalamus sinicus* (Poaceae), the strongest woody bamboo in the world	American Journal of Botany	Article	0002-9122	Sep	2012	99	9	E347	E349	10.3732/ajb.1200029
464	Mfegue, CV et al.	Microsatellite markers for population studies of *Phytophthora megakarya* (Pythiaceae), a cacao pathogen in Africa	American Journal of Botany	Article	0002-9122	Sep	2012	99	9	E353	E356	10.3732/ajb.1200053
465	Nemati, Z et al.	Isolation and characterization of a first set of polymorphic microsatellite markers in saffron, *Crocus sativus* (Iridaceae)	American Journal of Botany	Article	0002-9122	Sep	2012	99	9	E340	E343	10.3732/ajb.1100531
466	Nie, XJ et al.	Development of chromosome-arm-specific microsatellite markers in *Triticum aestivum* (Poaceae) using NGS technology	American Journal of Botany	Article	0002-9122	Sep	2012	99	9	E369	E371	10.3732/ajb.1200077
467	Pramod, S et al.	Gene expression assays for actin, ubiquitin, and three microsatellite-encoding genes in *Helianthus annuus* (Asteraceae)	American Journal of Botany	Article	0002-9122	Sep	2012	99	9	E350	E352	10.3732/ajb.1200045
468	Veliz, D et al.	Characterization of novel microsatellite markers for *Eschscholzia californica* (Papaveraceae), an invasive species in central chile	American Journal of Botany	Article	0002-9122	Sep	2012	99	9	E366	E368	10.3732/ajb.1200076
469	Vik, U et al.	Microsatellite markers for *Hylocomium splendens* (Hylocomiaceae)	American Journal of Botany	Article	0002-9122	Sep	2012	99	9	E344	E346	10.3732/ajb.1200028
470	Wang, L et al.	Development of 35 microsatellite markers from heat stress transcription factors in *Populus simonii* (Salicaceae)	American Journal of Botany	Article	0002-9122	Sep	2012	99	9	E357	E361	10.3732/ajb.1200056
471	Honig, JA et al.	Classification of Kentucky bluegrass (*Poa pratensis* L.) cultivars and accessions based on microsatellite (Simple Sequence Repeat) markers	Hortscience	Article	0018-5345	Sep	2012	47	9	1356	1366	NA
472	Arismendi, MJ et al.	Characterization of genetic diversity of stone fruit rootstocks used in Chile by means of microsatellite markers	Journal of the American Society for Horticultural Science	Article	0003-1062	Sep	2012	137	5	302	310	NA
473	Guo, J et al.	Population structure of the wild soybean (*Glycine soja*) in China: implications from microsatellite analyses	Annals of Botany	Article	0305-7364	Sep	2012	110	4	777	785	10.1093/aob/mcs142
474	Garcia-Fernandez, A et al.	Isolation and characterization of 10 microsatellite loci in *Cneorum tricoccon* (Cneoraceae), a Mediterranean	American Journal of Botany	Article	0002-9122	Aug	2012	99	8	E307	E309	10.3732/ajb.1100589

	Author	Publication title	Source	Publication type	ISSN	Publication date		Vol	Issue	Pages		DOI
		relict plant										
475	Gong, W et al.	Development of microsatellite markers from *Cercis chinensis* (Fabaceae)	American Journal of Botany	Article	0002-9122	Aug	2012	99	8	E337	E339	10.3732/ajb.1200065
476	Heer, K et al.	Anonymous and EST-based microsatellite DNA markers that transfer broadly across the fig tree genus (*Ficus*, Moraceae)	American Journal of Botany	Article	0002-9122	Aug	2012	99	8	E330	E333	10.3732/ajb.1200032
477	Koffi, KG et al.	Characterization of new microsatellite loci isolated from *Santiria trimera* (Burseraceae)	American Journal of Botany	Article	0002-9122	Aug	2012	99	8	E334	E336	10.3732/ajb.1200041
478	Lin, H et al.	Microsatellite markers for *Duperrea pavettifolia* (Rubiaceae)	American Journal of Botany	Article	0002-9122	Aug	2012	99	8	E310	E312	10.3732/ajb.1100609
479	McLay, TGB et al.	Microsatellite markers for the endangered root holoparasite *Dactylanthus taylorii* (Balanophoraceae) from 454 pyrosequencing	American Journal of Botany	Article	0002-9122	Aug	2012	99	8	E323	E325	10.3732/ajb.1200013
480	Mishima, K et al.	Isolation and characterization of microsatellite markers for *Thujopsis dolabrata* var. *hondai* (Cupressaceae)	American Journal of Botany	Article	0002-9122	Aug	2012	99	8	E317	E319	10.3732/ajb.1200010
481	Qi, XS et al.	Development of 12 microsatellite markers for *Platycrater arguta* (Hydrangeaceae) endemic to East Asia	American Journal of Botany	Article	0002-9122	Aug	2012	99	8	E304	E306	10.3732/ajb.1100582
482	Santos, FRC et al.	Isolation and characteristics of eight novel polymorphic microsatellite loci in *Lippia alba* (Verbenaceae)	American Journal of Botany	Article	0002-9122	Aug	2012	99	8	E301	E303	10.3732/ajb.1100578
483	Skogen, KA et al.	Microsatellite primers in *Oenothera harringtonii* (Onagraceae), an annual endemic to the shortgrass prairie of Colorado	American Journal of Botany	Article	0002-9122	Aug	2012	99	8	E313	E316	10.3732/ajb.1200003
484	Zeng, LY et al.	Microsatellite markers for *Saussurea gnaphalodes* (Asteraceae), a native himalayan mountain species	American Journal of Botany	Article	0002-9122	Aug	2012	99	8	E326	E329	10.3732/ajb.1200019
485	Sathuvalli, VR; Mehlenbacher, SA	Characterization of American hazelnut (*Corylus americana*) accessions and *Corylus americana* x *Corylus avellana* hybrids using microsatellite markers	Genetic Resources & Crop Evolution	Article	0925-9864	Aug	2012	59	6	1055	1075	10.1007/s10722-011-9743-0
486	James, BT et al.	Development of microsatellite markers in autopolyploid sugarcane and comparative analysis of conserved microsatellites in sorghum and sugarcane	Molecular Breeding	Article	1380-3743	Aug	2012	30	2	661	669	10.1007/s11032-011-9651-1
487	Shiferaw, E et al.	Exploring the genetic diversity of Ethiopian grass pea (*Lathyrus sativus* L.) using EST-SSR markers	Molecular Breeding	Article	1380-3743	Aug	2012	30	2	789	797	10.1007/s11032-011-9662-y
488	Jimenez, OR; Korpelainen, H	Microsatellite markers reveal promising genetic diversity and seed trait associations in common bean landraces (*Phaseolus vulgaris* L.) from Nicaragua	Plant Genetic Resources	Article	1479-2621	Aug	2012	10	2	108	118	10.1017/S1479262112000081
489	Geleta, M et al.	Assigning *Brassica* microsatellite markers to the nine C-genome chromosomes using *Brassica rapa* var. *trilocularis-B. oleracea* var. *alboglabra* monosomic alien addition lines	Theoretical & Applied Genetics	Article	0040-5752	Aug	2012	125	3	455	466	10.1007/s00122-012-1845-3
490	Franzoni, J et al.	Application of microsatellite markers to evaluate the heterozygosity from the popcorn composite CMS-43 (*Zea mays* L.) during eight cycles of selection	Plant Breeding	Article	0179-9541	Aug	2012	131	4	479	485	10.1111/j.1439-0523.2012.01981.x
491	Yang, M et al.	Comparative analysis of genetic diversity of lotus (*Nelumbo*) using SSR and SRAP markers	Scientia Horticulturae	Article	0304-4238	Jul	2012	142	NA	185	195	10.1016/j.scienta.2012.05.021
492	Islam, MN et al.	DNA fingerprinting and genotyping of cotton varieties using SSR markers	Notulae Botanicae Horti Agrobotanici Cluj-Napoca	Article	0255-965X	Jul-Dec	2012	40	2	261	265	NA
493	Ashizawa, K et al.	Development of microsatellite markers in a riparian shrub, *Spiraea thunbergii* (Rosaceae)	American Journal of Botany	Article	0002-9122	Jul	2012	99	7	E283	E285	10.3732/ajb.1100587
494	Lindsay, DL et al.	Novel microsatellite loci for *Agave parryi* and cross-amplification in *Agave palmeri* (Agavaceae)	American Journal of Botany	Article	0002-9122	Jul	2012	99	7	E295	E297	10.3732/ajb.1200033
495	Martins, APV et al.	Microsatellite markers for *Vellozia gigantea* (Velloziaceae), a narrowly endemic species to the Brazilian campos rupestres	American Journal of Botany	Article	0002-9122	Jul	2012	99	7	E289	E291	10.3732/ajb.1100611
496	Pate, SJ et al.	Development and characterization of microsatellite markers for *Actaea racemosa* (black cohosh, Ranunculaceae)	American Journal of Botany	Article	0002-9122	Jul	2012	99	7	E274	E276	10.3732/ajb.1100577
497	Qian, ML et al.	Development of microsatellite markers for the invasive weed *Parthenium hysterophorus* (Asteraceae)	American Journal of Botany	Article	0002-9122	Jul	2012	99	7	E277	E279	10.3732/ajb.1100579
498	Sun, MZ et al.	Genomic and est-derived microsatellite markers for *Iris laevigata* (Iridaceae) and other congeneric species	American Journal of Botany	Article	0002-9122	Jul	2012	99	7	E286	E288	10.3732/ajb.1100608

	Author	Publication title	Source	Publication type	ISSN	Publication date		Vol	Issue	Pages		DOI
499	Wu, CA et al.	Microsatellite loci in *Ipomopsis aggregata* (Polemoniaceae) and cross-species applicability for ecological genetics studies	American Journal of Botany	Article	0002-9122	Jul	2012	99	7	E298	E300	10.3732/ajb.1100612
500	Zhu, L; Lou, AR	Development and characterization of nine highly polymorphic microsatellite primers for *Platycladus orientalis* (Cupressaceae)	American Journal of Botany	Article	0002-9122	Jul	2012	99	7	E280	E282	10.3732/ajb.1100583
501	Zhang, HY et al.	Identification and validation of a core set of microsatellite markers for genetic diversity analysis in watermelon, *Citrullus lanatus* Thunb. Matsum. & Nakai	Euphytica	Article	0014-2336	Jul	2012	186	2	329	342	10.1007/s10681-011-0574-z
502	Velez, MD; Ibanez, J	Assessment of the uniformity and stability of grapevine cultivars using a set of microsatellite markers	Euphytica	Article	0014-2336	Jul	2012	186	2	419	432	10.1007/s10681-012-0633-0
503	Papi, RM et al.	Genetic variation of *Fraxinus angustifolia* natural populations in Greece based on nuclear and chloroplast microsatellite markers	European Journal of Forest Research	Article	1612-4669	Jul	2012	131	4	1151	1161	10.1007/s10342-011-0586-1
504	Turi, NA et al.	Genetic diversity in the locally collected *Brassica* species of Pakistan based on microsatellite markers	Pakistan Journal of Botany	Article	0556-3321	Jun	2012	44	3	1029	1035	NA
505	Billot, C et al.	A reference microsatellite kit to assess for genetic diversity of *Sorghum bicolor* (Poaceae)	American Journal of Botany	Article	0002-9122	Jun	2012	99	6	E245	E250	10.3732/ajb.1100548
506	Bressan, ED et al.	Development of microsatellite primers for *Jatropha curcas* (Euphorbiaceae) and transferability to congeners	American Journal of Botany	Article	0002-9122	Jun	2012	99	6	E237	E239	10.3732/ajb.1100532
507	Chiang, YC et al.	Characterization of microsatellite loci from *Litsea hypophaea* (Lauraceae), a tree endemic to Taiwan	American Journal of Botany	Article	0002-9122	Jun	2012	99	6	E251	E254	10.3732/ajb.1100551
508	Jeong, KS et al.	Isolation and characterization of microsatellite markers from *Tiarella polyphylla* (Saxifragaceae)	American Journal of Botany	Article	0002-9122	Jun	2012	99	6	E255	E257	10.3732/ajb.1100553
509	Jiang, JX et al.	Development of novel chloroplast microsatellite markers for *Miscanthus* species (Poaceae)	American Journal of Botany	Article	0002-9122	Jun	2012	99	6	E230	E233	10.3732/ajb.1100518
510	Li, JM et al.	Development of microsatellite markers in *Parakmeria nitida* (Magnoliaceae)	American Journal of Botany	Article	0002-9122	Jun	2012	99	6	E234	E236	10.3732/ajb.1100526
511	Li, L et al.	Isolation and characterization of microsatellite markers from *Clematoclethra scandens* (Actinidiaceae)	American Journal of Botany	Article	0002-9122	Jun	2012	99	6	E258	E261	10.3732/ajb.1100556
512	Vik, U et al.	Microsatellite markers for *Bistorta vivipara* (Polygonaceae)	American Journal of Botany	Article	0002-9122	Jun	2012	99	6	E226	E229	10.3732/ajb.1100504
513	Xu, C et al.	Microsatellite primers for the endangered aquatic herb, *Ottelia acuminata* (Hydrocharitaceae)	American Journal of Botany	Article	0002-9122	Jun	2012	99	6	E262	E264	10.3732/ajb.1100563
514	Xue, JH et al.	Polymorphic chloroplast microsatellite loci in *Nelumbo* (Nelumbonaceae)	American Journal of Botany	Article	0002-9122	Jun	2012	99	6	E240	E244	10.3732/ajb.1100547
515	Keneni, G et al.	Genetic diversity and population structure of Ethiopian chickpea (*Cicer arietinum* L.) germplasm accessions from different geographical origins as revealed by microsatellite markers	Plant Molecular Biology Reporter	Article	0735-9640	Jun	2012	30	3	654	665	10.1007/s11105-011-0374-6
516	Ji, Y et al.	Development of polymorphic microsatellite loci in *Momordica charantia* (Cucurbitaceae) and their transferability to other cucurbit species	Scientia Horticulturae	Article	0304-4238	Jun1	2012	140	NA	115	118	10.1016/j.scienta.2012.03.024
517	Sardos, J et al.	Genetic diversity of taro (*Colocasia esculenta* (L.) Schott) in Vanuatu (Oceania): an appraisal of the distribution of allelic diversity (DAD) with SSR markers	Genetic Resources & Crop Evolution	Article	0925-9864	Jun	2012	59	5	805	820	10.1007/s10722-011-9720-7
518	Haque, MA et al.	Microsatellite mapping of genes for semi-dwarfism and branched spike in *Triticum durum* Desf. var. *ramosoobscurum* Jakubz. "Vetvistokoloskaya"	Genetic Resources & Crop Evolution	Article	0925-9864	Jun	2012	59	5	831	837	10.1007/s10722-011-9722-5
519	Georgi, L et al.	Cranberry microsatellite marker development from assembled next-generation genomic sequence	Molecular Breeding	Article	1380-3743	Jun	2012	30	1	227	237	10.1007/s11032-011-9613-7
520	Wang, YH et al.	Identification of SSR markers associated with height using pool-based genome-wide association mapping in sorghum	Molecular Breeding	Article	1380-3743	Jun	2012	30	1	281	292	10.1007/s11032-011-9617-3
521	Gupta, D et al.	Integration of EST-SSR markers of *Medicago truncatula* into intraspecific linkage map of lentil and identification of QTL conferring resistance to ascochyta blight at seedling and pod stages	Molecular Breeding	Article	1380-3743	Jun	2012	30	1	429	439	10.1007/s11032-011-9634-2

	Author	Publication title	Source	Publication type	ISSN	Publication date		Vol	Issue	Pages		DOI
522	Koelling, J et al.	Development of new microsatellite markers (SSRs) for *Humulus lupulus*	Molecular Breeding	Article	1380-3743	Jun	2012	30	1	479	484	10.1007/s11032-011-9637-z
523	He, XY; Bjornstad, A	Diversity of North European oat analyzed by SSR, AFLP and DART markers	Theoretical & Applied Genetics	Article	0040-5752	Jun	2012	125	1	57	70	10.1007/s00122-012-1816-8
524	Singh, RK et al.	Rapid DNA extraction protocol for high throughput microsatellite/molecular analysis in pigeon pea	Vegetos	Article	0970-4078	Jun	2012	25	1	30	33	NA
525	Zhu, XC et al.	Evaluation of simple sequence repeat (SSR) markers from *Solanum* crop species for *Solanum elaeagnifolium*	Weed Research	Article	0043-1737	Jun	2012	52	3	217	223	10.1111/j.1365-3180.2012.00908.x
526	Liu, YL et al.	Characterization of microsatellite markers and their application for the assessment of genetic diversity among *Lotus* accessions	Journal of the American Society for Horticultural Science	Article	0003-1062	May	2012	137	3	180	188	NA
527	Trigiano, RN et al.	Ten polymorphic microsatellite loci identified from a small insert genomic library for *Peronospora tabacina*	Mycologia	Article	0027-5514	May-Jun	2012	104	3	633	640	10.3852/11-288
528	Allen, JM et al.	Development and characterization of microsatellite markers for *Berberis thunbergii* (Berberidaceae)	American Journal of Botany	Article	0002-9122	May	2012	99	5	E220	E222	10.3732/ajb.1100530
529	Martin, MA et al.	Microsatellite development for the relictual conifer *Araucaria araucana* (Araucariaceae) using next-generation sequencing	American Journal of Botany	Article	0002-9122	May	2012	99	5	E213	E215	10.3732/ajb.1100519
530	Chiou, CY et al.	Development and characterization of 38 polymorphic microsatellite markers from an economically important fruit tree, the Indian jujube	American Journal of Botany	Article	0002-9122	May	2012	99	5	E199	E202	10.3732/ajb.1100500
531	Cruz, MV et al.	Isolation and characterization of microsatellite markers for *Plathymenia reticulata* (Fabaceae)	American Journal of Botany	Article	0002-9122	May	2012	99	5	E210	E212	10.3732/ajb.1100511
532	Du, YJ et al.	Development of microsatellite markers for the dove tree, *Davidia involucrata* (Nyssaceae), a rare endemic from China	American Journal of Botany	Article	0002-9122	May	2012	99	5	E206	E209	10.3732/ajb.1100507
533	Garcia, M et al.	Portable microsatellite primers for *Ficus* (Moraceae)	American Journal of Botany	Article	0002-9122	May	2012	99	5	E187	E192	10.3732/ajb.1100485
534	Hyun, YS et al.	Development of polymorphic microsatellite markers for *Cymbidium goeringii* (Orchidaceae)	American Journal of Botany	Article	0002-9122	May	2012	99	5	E193	E198	10.3732/ajb.1100505
535	Li, ZZ et al.	Microsatellite primers in the endangered quillwort *Isoetes hypsophila* (Isoetaceae) and cross-amplification in *I. sinensis*	American Journal of Botany	Article	0002-9122	May	2012	99	5	E184	E186	10.3732/ajb.1100319
536	Wen, Q et al.	Development of polymorphic microsatellite markers in *Camellia chekiangoleosa* (Theaceae) using 454-ESTs	American Journal of Botany	Article	0002-9122	May	2012	99	5	E203	E205	10.3732/ajb.1100486
537	Gong, L; Deng, ZA	Selection and application of SSR markers for variety discrimination, genetic similarity and relation analysis in gerbera (*Gerbera hybrida*)	Scientia Horticulturae	Article	0304-4238	May	2012	138	NA	120	127	10.1016/j.scienta.2012.02.020
538	Parvaresh, M et al.	Molecular diversity and genetic relationship of pomegranate (*Punica granatum* L.) genotypes using microsatellite markers	Scientia Horticulturae	Article	0304-4238	May	2012	138	NA	244	252	10.1016/j.scienta.2012.02.038
539	Mitsui, Y; Setoguchi, H	Recent origin and adaptive diversification of *Ainsliaea* (Asteraceae) in the Ryukyu Islands: molecular phylogenetic inference using nuclear microsatellite markers	Plant Systematics & Evolution	Article	0378-2697	May	2012	298	5	985	996	10.1007/s00606-012-0608-6
540	Sargent, DJ et al.	A microsatellite linkage map for the cultivated strawberry (*Fragaria* x *ananassa*) suggests extensive regions of homozygosity in the genome that may have resulted from breeding and selection	Theoretical & Applied Genetics	Article	0040-5752	May	2012	124	7	1229	1240	10.1007/s00122-011-1782-6
541	Hung, KH et al.	Isolation and characterization of microsatellite loci from *Pinus massoniana* (Pinaceae)	Botanical Studies	Article	1817-406X	Apr	2012	53	2	191	196	NA
542	Cerqueira-Silva, CBM et al.	Development and characterization of microsatellite markers for the wild south American *Passiflora cincinnata* (Passifloraceae)	American Journal of Botany	Article	0002-9122	Apr	2012	99	4	E170	E172	10.3732/ajb.1100477
543	Feres, JM et al.	Development of microsatellite markers for *Anadenanthera colubrina* (Leguminosae), a neotropical tree species	American Journal of Botany	Article	0002-9122	Apr	2012	99	4	E154	E156	10.3732/ajb.1100446
544	Ho, CS et al.	Isolation and characterization of 15 microsatellite loci in four endangered *Amentotaxus* species (Taxaceae)	American Journal of Botany	Article	0002-9122	Apr	2012	99	4	E157	E159	10.3732/ajb.1100452
545	Ma, WW et al.	Development and characterization of microsatellite markers for *Emmenopterys henryi* (Rubiaceae), a rare tree from China	American Journal of Botany	Article	0002-9122	Apr	2012	99	4	E179	E181	10.3732/ajb.1100495

	Author	Publication title	Source	Publication type	ISSN	Publication date		Vol	Issue	Pages		DOI
546	Rakotondralambo, SOR et al.	Microsatellite markers isolated from the wild medicinal plant *Centella asiatica* (Apiaceae) from an enriched genomic library	American Journal of Botany	Article	0002-9122	Apr	2012	99	4	E176	E178	10.3732/ajb.1100441
547	Tew, JM et al.	Microsatellite development for an endangered riparian inhabitant, *Lilaeopsis schaffneriana* subsp *recurva* (Apiaceae)	American Journal of Botany	Article	0002-9122	Apr	2012	99	4	E164	E166	10.3732/ajb.1100517
548	Wang, L et al.	Isolation and characterization of microsatellite loci in the endangered tree *Diplopanax stachyanthus* (Araliaceae)	American Journal of Botany	Article	0002-9122	Apr	2012	99	4	E167	E169	10.3732/ajb.1100476
549	Wohrmann, T et al.	Development of microsatellite markers in *Fosterella rusbyi* (Bromeliaceae) using 454 pyrosequencing	American Journal of Botany	Article	0002-9122	Apr	2012	99	4	E160	E163	10.3732/ajb.1100470
550	Norouzi, M et al.	Chloroplast microsatellite diversity and population genetic structure of Iranian pomegranate (*Punica granatum* L.) genotypes	Scientia Horticulturae	Article	0304-4238	Apr1	2012	137	NA	114	120	10.1016/j.scienta.2012.01.034
551	Datta, S et al.	Conservation of microsatellite regions across legume genera enhances marker repertoire and genetic diversity study in *Phaseolus* genotypes	Plant Breeding	Article	0179-9541	Apr	2012	131	2	307	311	10.1111/j.1439-0523.2011.01892.x
552	Antonius, K et al.	Development of the Northern European *Ribes* core collection based on a microsatellite (SSR) marker diversity analysis	Plant Genetic Resources	Article	1479-2621	Apr	2012	10	1	70	73	10.1017/S1479262111000980
553	Kim, C et al.	Molecular identification of *Schoenoplectiella species* (Cyperaceae) by use of microsatellite markers	Plant Systematics & Evolution	Article	0378-2697	Apr	2012	298	4	811	817	10.1007/s00606-012-0592-x
554	Satish, K et al.	Molecular tagging and validation of microsatellite markers linked to the low germination stimulant gene (*lgs*) for Striga resistance in sorghum [*Sorghum bicolor* (L.) Moench]	Theoretical & Applied Genetics	Article	0040-5752	Apr	2012	124	6	989	1003	10.1007/s00122-011-1763-9
555	Barnaud, A et al.	Development of nuclear microsatellite markers for the fonio, *Digitaria exilis* (Poaceae), an understudied west african cereal	American Journal of Botany	Article	0002-9122	Mar	2012	99	3	E105	E107	10.3732/ajb.1100423
556	Burge, DO et al.	Microsatellite markers from *Ceanothus roderickii* (Rhamnaceae) using next-generation sequencing technology	American Journal of Botany	Article	0002-9122	Mar	2012	99	3	E127	E130	10.3732/ajb.1100431
557	Chiang, YC et al.	Development and characterization of 20 new polymorphic microsatellite markers from *Mangifera indica* (Anacardiaceae)	American Journal of Botany	Article	0002-9122	Mar	2012	99	3	E117	E119	10.3732/ajb.1100443
558	Croft, GK; Schaal, BA	Development of microsatellite markers in *Byrsonima crassifolia* (Malpighiaceae)	American Journal of Botany	Article	0002-9122	Mar	2012	99	3	E111	E113	10.3732/ajb.1100457
559	Gao, H et al.	Development of microsatellite primers of the largest seagrass, *Enhalus acoroides* (Hydrocharitaceae)	American Journal of Botany	Article	0002-9122	Mar	2012	99	3	E99	E101	10.3732/ajb.1100412
560	Kuester, AP; Nason, JD	Microsatellite loci for *Gossypium davidsonii* (Malvaceae) and other D-genome, Sonoran Desert endemic cotton species	American Journal of Botany	Article	0002-9122	Mar	2012	99	3	E91	E93	10.3732/ajb.1100421
561	Lowry, DB et al.	Microsatellite markers for the native texas perennial grass, *Panicum hallii* (Poaceae)	American Journal of Botany	Article	0002-9122	Mar	2012	99	3	E114	E116	10.3732/ajb.1100430
562	Niu, HY et al.	Isolation and characterization of 36 polymorphic microsatellite markers in *Schima superba* (Theaceae)	American Journal of Botany	Article	0002-9122	Mar	2012	99	3	E123	E126	10.3732/ajb.1100454
563	Ritter, LMO et al.	Development of microsatellite markers for *Qualea grandiflora* (Vochysiaceae), a typical species of the Brazilian cerrado	American Journal of Botany	Article	0002-9122	Mar	2012	99	3	E97	E98	10.3732/ajb.1100405
564	Souza, HAV et al.	Development of microsatellite markers for *Dimorphandra mollis* (Leguminosae), a widespread tree from the Brazilian cerrado	American Journal of Botany	Article	0002-9122	Mar	2012	99	3	E102	E104	10.3732/ajb.1100413
565	Tao, CC et al.	Microsatellite markers for the relictual dove tree, *Davidia involucrata* (Cornaceae)	American Journal of Botany	Article	0002-9122	Mar	2012	99	3	E108	E110	10.3732/ajb.1100414
566	Tong, X et al.	Development and characterization of polymorphic microsatellite markers in *Cyclobalanopsis glauca* (Fagaceae)	American Journal of Botany	Article	0002-9122	Mar	2012	99	3	E120	E122	10.3732/ajb.1100448
567	Matsumoto, Y et al.	Cross-species transferability of 86 cucumber (*Cucumis sativus* L.) microsatellite markers to gherkin (*C. anguria* L.)	Scientia Horticulturae	Article	0304-4238	Mar	2012	136	NA	110	114	10.1016/j.scienta.2012.01.009
568	Reddy, RN et al.	Characterization, development and mapping of unigene-derived microsatellite markers in sorghum [*Sorghum bicolor* (L.) Moench]	Molecular Breeding	Article	1380-3743	Mar	2012	29	3	543	564	10.1007/s11032-011-9571-0

	Author	Publication title	Source	Publication type	ISSN	Publication date		Vol	Issue	Pages		DOI
569	van Dijk, T et al.	Microsatellite allele dose and configuration establishment (MADCE): an integrated approach for genetic studies in allopolyploids	BMC Plant Biology	Article	1471-2229	Feb	2012	12	NA	NA	NA	10.1186/1471-2229-12-25
570	Lopez-Roberts, MC et al.	Microsatellite marker development for the threatened orchid *Masdevallia solomonii* (Orchidaceae)	American Journal of Botany	Article	0002-9122	Feb	2012	99	2	E66	E68	10.3732/ajb.1100364
571	Du, QZ et al.	Development of 15 novel microsatellite markers from cellulose synthase genes in *Populus tomentosa* (Salicaceae)	American Journal of Botany	Article	0002-9122	Feb	2012	99	2	E46	E48	10.3732/ajb.1100308
572	Gode, C et al.	Nuclear microsatellite loci for *Arabidopsis halleri* (Brassicaceae), a model species to study plant adaptation to heavy metals	American Journal of Botany	Article	0002-9122	Feb	2012	99	2	E49	E52	10.3732/ajb.1100320
573	Gowda, V et al.	Development and characterization of microsatellite loci for two caribbean *Heliconia* (Heliconiaceae: *H. bihai* and *H. caribaea*)	American Journal of Botany	Article	0002-9122	Feb	2012	99	2	E81	E83	10.3732/ajb.1100386
574	Li, ZZ et al.	Microsatellite primers in the Chinese dove tree, *Davidia involucrata* (Cornaceae), a relic species of the tertiary	American Journal of Botany	Article	0002-9122	Feb	2012	99	2	E78	E80	10.3732/ajb.1100365
575	Michalski, SG; Durka, W	Identification and characterization of microsatellite loci in the rush *Juncus effusus* (Juncaceae)	American Journal of Botany	Article	0002-9122	Feb	2012	99	2	E53	E55	10.3732/ajb.1100322
576	Soares, TN et al.	Development of microsatellite markers for the neotropical tree species *Dipteryx alata* (Fabaceae)	American Journal of Botany	Article	0002-9122	Feb	2012	99	2	E72	E73	10.3732/ajb.1100377
577	Wu, B et al.	Isolation and characterization of novel microsatellite markers for *Avena sativa* (Poaceae) (oat)	American Journal of Botany	Article	0002-9122	Feb	2012	99	2	E69	E71	10.3732/ajb.1100404
578	Yuan, N et al.	Development of microsatellite markers in heterostylous *Hedyotis chrysotricha* (Rubiaceae)	American Journal of Botany	Article	0002-9122	Feb	2012	99	2	E43	E45	10.3732/ajb.1100304
579	Babu BK et al.	Identification of candidate gene-based SSR markers for lysine and tryptophan metabolic pathways in maize (*Zea mays*)	Plant Breeding	Article	0179-9541	Feb	2012	131	1	20	27	10.1111/j.1439-0523.2011.01919.x
580	Fang, W et al.	Genetic diversity and relationship of clonal tea (*Camellia sinensis*) cultivars in China as revealed by SSR markers	Plant Systematics & Evolution	Article	0378-2697	Feb	2012	298	2	469	483	10.1007/s00606-011-0559-3
581	Arabnezhad, H et al.	Development, characterization and use of microsatellite markers for germplasm analysis in date palm (*Phoenix dactylifera* L.)	Scientia Horticulturae	Article	0304-4238	Feb1	2012	134	NA	150	156	10.1016/j.scienta.2011.11.032
582	Ma, JQ et al.	Microsatellite markers from tea plant expressed sequence tags (ESTs) and their applicability for cross-species/genera amplification and genetic mapping	Scientia Horticulturae	Article	0304-4238	Feb1	2012	134	NA	167	175	10.1016/j.scienta.2011.10.029
583	Gupta, S et al.	Sequence-based novel genomic microsatellite markers for robust genotyping purposes in foxtail millet [*Setaria italica* (L.) P. Beauv.]	Plant Cell Reports	Article	0721-7714	Feb	2012	31	2	323	337	10.1007/s00299-011-1168-x
584	Tranbarger, TJ et al.	SSR markers in transcripts of genes linked to post-transcriptional and transcriptional regulatory functions during vegetative and reproductive development of *Elaeis guineensis*	BMC Plant Biology	Article	1471-2229	Jan 3	2012	12	NA	NA		10.1186/1471-2229-12-1
585	Espinoza, S et al.	Genetic diversity and differentiation of Chilean plantations of *Pinus radiata* D. Don using microsatellite DNA markers	Silvae Genetica	Article	0037-5349	NA	2012	61	6	221	228	NA
586	Wen, Y et al.	Cross-species amplification of microsatellite loci for the endangered conifer, *Taxus chinensis* var. *mairei* (Taxaceae)	Silvae Genetica	Article	0037-5349	NA	2012	61	6	287	291	NA
587	Liesebach, H; Ewald, E	Optimisation of a multiplex PCR assay of nuclear microsatellite markers for population genetics and clone identification in *Robinia pseudoacacia* L.	Silvae Genetica	Article	0037-5349	NA	2012	61	4-5	142	148	NA
588	Bucci, C et al.	The use of microsatellite markers for germplasm management in spanish and Italian olive collections	IHC 2010: Olive Trends Symposium - From the Olive Tree to Olive Oil: New Trends & Future Challenges, Acta Horticulturae	Proceedings Paper	0567-7572BN 978-90-66055-94-0	Aug	2012	924	NA	349	354	NA
589	Tejaswini Madhavilatha, P et al.	Microsatellite markers in comparison with morphological characters for protection of plant cultivars in carnation	IHC 2010: International Symposium on Genomics & Genetic Transformation of Horticultural Crops, Acta Horticulturae	Proceedings Paper	0567-7572BN 978-90-66050-48-8	Aug	2012	929	NA	453	458	NA

	Author	Publication title	Source	Publication type	ISSN	Publication date		Vol	Issue	Pages		DOI
590	Cachi, AM; Wunsch, A	Identification of a microsatellite marker linked to self-compatibility in ‘Cristobalina’ sweet cherry	IHC 2010: International Symposium on Plant Physiology From Cell to Fruit Production System, Acta Horticulturae	Proceedings Paper	0567-7572BN 978-90-66051-18-8	Aug	2012	932	NA	73	77	NA
591	Naghavi, MR et al.	Genetic diversity in Iranian chickpea (*Cicer arietinum* L.) landraces as revealed by microsatellite markers	Czech Journal of Genetics & Plant Breeding	Article	1212-1975	NA	2012	48	3	131	138	NA
592	Jimenez, OR et al.	Genetic purity of common bean seed generations (*Phaseolus vulgaris* cv. INTA ROJO) as tested with microsatellite markers	Seed Science & Technology	Article	0251-0952	NA	2012	40	1	73	85	NA
593	Nookaraju, A; Agrawal, DC	Genetic homogeneity of *in vitro* raised plants of grapevine cv. Crimson Seedless revealed by ISSR and microsatellite markers	South African Journal of Botany	Article	0254-6299	Jan	2012	78	NA	302	306	10.1016/j.sajb.2011.08.009
594	Chen, YY et al.	Microsatellite analysis reveals the genetic structure and gene flow of the aquatic quillwort *Isoetes sinensis*, a critically endangered species in China	Aquatic Botany	Article	0304-3770	Jan	2012	96	1	52	57	10.1016/j.aquabot.2011.09.001
595	Barrett, MD et al.	Characterization and cross application of novel microsatellite markers for a rare sedge, *Lepidosperma gibsonii* (Cyperaceae)	American Journal of Botany	Article	0002-9122	Jan	2012	99	1	E14	E16	10.3732/ajb.1100357
596	Chiang, YC et al.	Characterization of 24 transferable microsatellite loci in four skullcaps (*Scutellaria*, Labiatae)	American Journal of Botany	Article	0002-9122	Jan	2012	99	1	E24	E27	10.3732/ajb.1100279
597	Cunha, CP et al.	New microsatellite markers for garlic, *Allium sativum* (Alliaceae)	American Journal of Botany	Article	0002-9122	Jan	2012	99	1	E17	E19	10.3732/ajb.1100278
598	Kameyama, Y	Development of microsatellite markers for *Cinnamomum camphora* (Lauraceae)	American Journal of Botany	Article	0002-9122	Jan	2012	99	1	E1	E3	NA
599	Nakamura, K et al.	Isolation of compound microsatellite markers in *Begonia fenicis* (Begoniaceae) endemic to East and Southeast Asian islands	American Journal of Botany	Article	0002-9122	Jan	2012	99	1	E20	E23	10.3732/ajb.1100297
600	Rodrigues, AG et al.	Development and characterization of polymorphic microsatellite markers for *Conopholis americana* (Orobanchaceae)	American Journal of Botany	Article	0002-9122	Jan	2012	99	1	E4	E6	10.3732/ajb.1100269
601	Setsuko, S et al.	Rapid development of microsatellite markers for *Pandanus boninensis* (Pandanaceae) by pyrosequencing technology	American Journal of Botany	Article	0002-9122	Jan	2012	99	1	E33	E37	10.3732/ajb.1100300
602	Setsuko, S et al.	Microsatellite markers derived from *Calophyllum inophyllum* (Clusiaceae) expressed sequence tags	American Journal of Botany	Article	0002-9122	Jan	2012	99	1	E28	E32	10.3732/ajb.1100299
603	Tang, M et al.	Microsatellite markers for the chinese endangered and endemic orchid *Cymbidium tortisepalum* (Orchidaceae)	American Journal of Botany	Article	0002-9122	Jan	2012	99	1	E11	E13	10.3732/ajb.1100307
604	Ravishankar, KV et al.	Development of SSR markers based on a survey of genomic sequences and their molecular analysis in banana (*Musa* spp.)	Journal Of Horticultural Science & Biotechnology	Article	1462-0316	Jan	2012	87	1	84	88	NA
605	Wu, B et al.	Recombinant microsatellite amplification: a rapid method for developing simple sequence repeat markers	Molecular Breeding	Article	1380-3743	Jan	2012	29	1	53	59	10.1007/s11032-010-9525-y
606	Carlier, JD et al.	A genetic map of pineapple (*Ananas comosus* (L.) Merr.) including SCAR, CAPS, SSR and EST-SSR markers	Molecular Breeding	Article	1380-3743	Jan	2012	29	1	245	260	10.1007/s11032-010-9543-9
607	Rahemi, A et al.	Genetic diversity of some wild almonds and related *Prunus* species revealed by SSR and EST-SSR molecular markers	Plant Systematics & Evolution	Article	0378-2697	Jan	2012	298	1	173	192	10.1007/s00606-011-0536-x
608	Serres-Giardi, L; Dogimont, C	How microsatellite diversity helps to understand the domestication history of melon	Cucurbitaceae 2012: Proceedings of the Xth Eucarpia Meeting on Genetics and Breeding of Cucurbitaceae	Proceedings Paper	NA	Oct	2012	NA	NA	254	263	NA
609	Yilmaz, N et al.	Evaluation of genetic relationships on single, triple and double cross melon (*Cucumis melo* var. *cantalupensis*) hybrids by SSR markers	Cucurbitaceae 2012: Proceedings of the Xth Eucarpia Meeting On Genetics and Breeding Of Cucurbitaceae	Proceedings Paper	NA	Oc	2012	NA	NA	544	550	NA
610	Kashiani, P et al.	Molecular characterization of tropical sweet corn inbred lines using microsatellite markers	Maydica	Article	0025-6153	NA	2012	57	1-4	154	163	NA
611	Ganopoulos, I et al.	Is the genetic diversity of small scattered forest tree populations at the southern limits of their range more prone to stochastic events? A wild cherry case study	Tree Genetics & Genomes	Article	1614-2942	Dec	2011	7	6	1299	1313	10.1007/s11295-011-0414-2

	Author	Publication title	Source	Publication type	ISSN	Publication date		Vol	Issue	Pages		DOI
		by microsatellite-based markers										
612	Araki, KS et al.	Isolation and characterization of microsatellite loci in a clonal herb, cardamine leucantha (Brassicaceae)	American Journal of Botany	Article	0002-9122	Dec	2011	98	12	E385	E387	10.3732/ajb.1100242
613	Segarra-Moragues, JG; Catalan, P	Characterization of microsatellite loci in *Festuca gautieri* (Poaceae) and transferability to *F. eskia* and *F. xpicoeuropeana*	American Journal of Botany	Article	0002-9122	Dec	2011	98	12	E360	E362	10.3732/ajb.1100267
614	Liu, WS et al.	Microsatellite primers in *Carex moorcroftii* (Cyperaceae), a dominant species of the steppe on the Qinghai-Tibetan Plateau	American Journal of Botany	Article	0002-9122	Dec	2011	98	12	E382	E384	10.3732/ajb.1100105
615	McEwen, JR et al.	Rapid isolation and cross-amplification of microsatellite markers in *Plectritis congesta* (Valerianaceae) with 454 sequencing	American Journal of Botany	Article	0002-9122	Dec	2011	98	12	E369	E371	10.3732/ajb.1100160
616	Nishizawa, T et al.	Development and characterization of a novel set of microsatellite markers for *Arisaema serratum* (Araceae)	American Journal of Botany	Article	0002-9122	Dec	2011	98	12	E378	E381	10.3732/ajb.1100274
617	Ohtsuki, T et al.	Isolation and characterization of microsatellite loci in the beach pea, *Lathyrus japonicus* (Fabaceae), in japan	American Journal of Botany	Article	0002-9122	Dec	2011	98	12	E375	E377	10.3732/ajb.1100268
618	Riley, L et al.	Microsatellite primers for the narrowly endemic shrub *Eriogonum giganteum* (Polygonaceae)	American Journal of Botany	Article	0002-9122	Dec	2011	98	12	E352	E355	10.3732/ajb.1100243
619	Wadl, PA et al.	Development of microsatellite loci for the endangered species *Pityopsis ruthii* (Asteraceae)	American Journal of Botany	Article	0002-9122	Dec	2011	98	12	E342	E345	10.3732/ajb.1100100
620	Yang, JY et al.	Chloroplast microsatellite primers for cacao (*Theobroma cacao*) and other Malvaceae	American Journal of Botany	Article	0002-9122	Dec	2011	98	12	E372	E374	10.3732/ajb.1100306
621	Zhang, ZR et al.	Development of 29 microsatellite markers for *Osmanthus fragrans* (Oleaceae), a traditional fragrant flowering tree of china	American Journal of Botany	Article	0002-9122	Dec	2011	98	12	E356	E359	10.3732/ajb.1100241
622	Mott, IW et al.	Simple sequence repeat (SSR) markers for *Elymus*, *Pseudoroegneria* and *Pascopyrum* species (Triticeae: Gramineae)	Plant Genetic Resources	Article	1479-2621	Dec	2011	9	4	489	494	10.1017/S1479262111000694
623	Couceiro, L et al.	Microsatellite development in *Rhodophyta* using high-throughput sequence data	Journal of Phycology	Article	0022-3646	Dec	2011	47	6	1258	1265	10.1111/j.1529-8817.2011.01075.x
624	Li, HT et al.	Development and genetic mapping of microsatellite markers from whole genome shotgun sequences in *Brassica oleracea*	Molecular Breeding	Article	1380-3743	Dec	2011	28	4	585	596	10.1007/s11032-010-9509-y
625	Li, JZ et al.	Development of microsatellite markers in canary seed (*Phalaris canariensis* L.)	Molecular Breeding	Article	1380-3743	Dec	2011	28	4	611	621	10.1007/s11032-010-9513-2
626	Njuguna, W et al.	Genetic diversity of diploid Japanese strawberry species based on microsatellite markers	Genetic Resources & Crop Evolution	Article	0925-9864	Dec	2011	58	8	1187	1198	10.1007/s10722-010-9652-7
627	Hou, XG et al.	Development of thirty new polymorphic microsatellite primers for *Paeonia suffruticosa*	Biologia Plantarum	Article	0006-3134	Dec	2011	55	4	708	710	10.1007/s10535-011-0172-x
628	Patzak, J; Matousek, J	Development and evaluation of expressed sequence tag-derived microsatellite markers for hop genotyping	Biologia Plantarum	Article	0006-3134	Dec	2011	55	4	761	765	10.1007/s10535-011-0183-7
629	Lu, L et al.	Genetic variation in pawpaw cultivars using microsatellite analysis	Journal of the American Society for Horticultural Science	Article	0003-1062	Nov	2011	136	6	415	421	NA
630	Dobrovolskaya, O et al.	Microsatellite mapping of *Ae. speltoides* and map-based comparative analysis of the S, G, and B genomes of Triticeae species	Theoretical & Applied Genetics	Article	0040-5752	Nov	2011	123	7	1145	1157	10.1007/s00122-011-1655-z
631	Dean, D et al.	Screening and characterization of 11 novel microsatellite markers from *Viburnum dilatatum*	Hortscience	Article	0018-5345	Nov	2011	46	11	1456	1459	NA
632	Chiang, YC et al.	Isolation of 16 polymorphic microsatellite markers from an endangered and endemic species, *Podocarpus nakaii* (Podocarpaceae)	American Journal of Botany	Article	0002-9122	Nov	2011	98	11	E306	E309	10.3732/ajb.1100229
633	Chung, KF et al.	Isolation and characterization of microsatellite loci in *Sassafras randaiense* (Lauraceae)	American Journal of Botany	Article	0002-9122	Nov	2011	98	11	E326	E329	10.3732/ajb.1100220
634	de Groot, GA et al.	Isolation of polymorphic microsatellite markers and tests of cross-amplification in four widespread european calcicole ferns	American Journal of Botany	Article	0002-9122	Nov	2011	98	11	E319	E322	10.3732/ajb.1100051
635	Ferreira-Ramos, R et al.	Microsatellite markers for *Aspidosperma polyneuron* (Apocynaceae), an endangered tropical tree species	American Journal of Botany	Article	0002-9122	Nov	2011	98	11	E300	E302	10.3732/ajb.1100222
636	Izuno, A et al.	Microsatellite loci in an endangered fern species, *Athyrium viridescentipes* (Woodsiaceae), and cross-species amplification	American Journal of Botany	Article	0002-9122	Nov	2011	98	11	E339	E341	10.3732/ajb.1100173

	Author	Publication title	Source	Publication type	ISSN	Publication date		Vol	Issue	Pages		DOI
637	Jennings, TN et al.	Microsatellite primers for the Pacific Northwest endemic conifer *Chamaecyparis lawsoniana* (Cupressaceae)	American Journal of Botany	Article	0002-9122	Nov	2011	98	11	E323	E325	10.3732/ajb.1100317
638	Man, YP et al.	Development of microsatellite markers in *Actinidia arguta* (Actinidiaceae) based on the NCBI data platform	American Journal of Botany	Article	0002-9122	Nov	2011	98	11	E310	E315	10.3732/ajb.1100182
639	Radosavljevic, I et al.	New microsatellite markers for *Salvia officinalis* (Lamiaceae) and cross-amplification in closely related species	American Journal of Botany	Article	0002-9122	Nov	2011	98	11	E316	E318	10.3732/ajb.1000462
640	Wang, YF et al.	Microsatellite primers in luohanguo (*Siraitia grosvenorii*, Cucurbitaceae), an economically important plant species	American Journal of Botany	Article	0002-9122	Nov	2011	98	11	E330	E332	10.3732/ajb.1100244
641	Miao, H et al.	A linkage map of cultivated cucumber (*Cucumis sativus* L.) with 248 microsatellite marker loci and seven genes for horticulturally important traits	Euphytica	Article	0014-2336	Nov	2011	182	2	167	176	10.1007/s10681-011-0410-5
642	Reid, A et al.	Construction of an integrated microsatellite and key morphological characteristic database of potato varieties on the EU common catalogue	Euphytica	Article	0014-2336	Nov	2011	182	2	239	249	10.1007/s10681-011-0462-6
643	Carneiro, FS et al.	Effects of selective logging on the mating system and pollen dispersal of *Hymenaea courbaril* L. (Leguminosae) in the Eastern Brazilian Amazon as revealed by microsatellite analysis	Forest Ecology And Management	Article	0378-1127	Nov	2011	262	9	1758	1765	10.1016/j.foreco.2011.07.023
644	Riahi, L et al.	Use of chloroplast microsatellite markers as a tool to elucidate polymorphism, classification and origin of Tunisian grapevines	Scientia Horticulturae	Article	0304-4238	Oct	2011	130	4	781	786	10.1016/j.scienta.2011.09.003
645	Chuang, HY et al.	Authentication of domestic Taiwan rice varieties based on fingerprinting analysis of microsatellite DNA markers	Botanical Studies	Article	1817-406X	Oct	2011	52	4	393	405	NA
646	Jiang, BA et al.	Retrotransposon- and microsatellite sequence-associated genomic changes in early generations of a newly synthesized allotetraploid *Cucumis* x *hytivus* Chen & Kirkbride	Plant Molecular Biology	Article	0167-4412	Oct	2011	77	3	225	233	10.1007/s11103-011-9804-y
647	Lee, CT et al.	Estimation of outcrossing rates in *Koompassia malaccensi*s from an open-pollinated population in Peninsular Malaysia using microsatellite markers	Journal of Tropical Forest Science	Article	0128-1283	Oct	2011	23	4	410	416	NA
648	Takayama, K et al.	A simple and cost-effective approach for microsatellite isolation in non-model plant species using small-scale 454 pyrosequencing	Taxon	Article	0040-0262	Oct	2011	60	5	1442	1449	NA
649	Elias, SM et al.	Microsatellite marker diversity and sequence polymorphism in the red gene locus of indigenous rice populations of Bangladesh	Plant Systematics & Evolution	Article	0378-2697	Oct	2011	296	3-4	157	165	10.1007/s00606-011-0482-7
650	Chen, C et al.	Isolation and characterization of microsatellite markers for *Dipteronia dyerana* (Sapindaceae), an endangered endemic species in china	American Journal of Botany	Article	0002-9122	Oct	2011	98	10	E271	E273	10.3732/ajb.1100185
651	Figueira, GM et al.	Development and characterization of microsatellite markers for *Hebanthe eriantha* (Amaranthaceae)	American Journal of Botany	Article	0002-9122	Oct	2011	98	10	E282	E283	10.3732/ajb.1100180
652	Harris, ESJ; Klooster, MR	Development of microsatellite markers for the medicinal plant *Isodon rubescens* (Lamiaceae) and related species	American Journal of Botany	Article	0002-9122	Oct	2011	98	10	E293	E295	10.3732/ajb.1100190
653	Munoz-Pajares, AJ et al.	Characterization of microsatellite loci in *Erysimum mediohispanicum* (Brassicaceae) and cross-amplification in related species	American Journal of Botany	Article	0002-9122	Oct	2011	98	10	E287	E289	10.3732/ajb.1100181
654	Jiang, JH et al.	Isolation and characterization of microsatellite loci in *Tsoongiodendron odorum* (Magnoliaceae)	American Journal of Botany	Article	0002-9122	Oct	2011	98	10	E284	E286	10.3732/ajb.1100221
655	Kophimai, Y et al.	Characterization of nuclear microsatellite loci in the calcareous fen specialist *Scorpidium cossonii* (Calliergonaceae)	American Journal of Botany	Article	0002-9122	Oct	2011	98	10	E290	E292	10.3732/ajb.1100144
656	Kriedt, RA et al.	Isolation, characterization, and cross-amplification of microsatellite markers for the *Petunia integrifolia* (Solanaceae) complex	American Journal of Botany	Article	0002-9122	Oct	2011	98	10	E277	E279	10.3732/ajb.1100178
657	Liu, H et al.	Development and characterization of microsatellite markers for *Panax notoginseng* (Araliaceae), a Chinese traditional herb	American Journal of Botany	Article	0002-9122	Oct	2011	98	10	E274	E276	10.3732/ajb.1100117

	Author	Publication title	Source	Publication type	ISSN	Publication date	Vol	Issue	Pages	DOI
658	Micheneau, C et al.	Development and characterization of microsatellite loci in *Pericopsis elata* (Fabaceae) using a cost-efficient approach	American Journal of Botany	Article	0002-9122	Oct 2011	98	10	E268 E270	10.3732/ajb.1100070
659	Nazareno, AG; dos Reis, MS	The same but different: monomorphic microsatellite markers as a new tool for genetic analysis	American Journal of Botany	Article	0002-9122	Oct 2011	98	10	E265 E267	10.3732/ajb.1100163
660	Wang, JY et al.	Identification and characterization of microsatellite markers from *Musa balbisiana*	Plant Breeding	Article	0179-9541	Oct 2011	130	5	584 590	10.1111/j.1439-0523.2011.01861.x
661	Gong, L; Deng, ZA	Development and characterization of microsatellite markers for caladiums (*Caladium* Vent.)	Plant Breeding	Article	0179-9541	Oct 2011	130	5	591 595	10.1111/j.1439-0523.2011.01863.x
662	Ono, NN et al.	Exploring the transcriptome landscape of pomegranate fruit peel for natural product biosynthetic gene and SSR marker discovery	Journal of Integrative Plant Biology	Article	1672-9072	Oct 2011	53	10	800 813	10.1111/j.1744-7909.2011.01073.x
663	Arias, RS et al.	Isolation and characterisation of the first microsatellite markers for *Cyperus rotundus*	Weed Research	Article	0043-1737	Oct 2011	51	5	451 460	10.1111/j.1365-3180.2011.00861.x
664	Carimi, F et al.	Intra-varietal genetic diversity of the grapevine (*Vitis vinifera* L.) cultivar Nero d'Avola as revealed by microsatellite markers	Genetic Resources & Crop Evolution	Article	0925-9864	Oct 2011	58	7	967 975	10.1007/s10722-011-9731-4
665	Loaisiga, CH et al.	Genetic diversity in seven populations of Nicaraguan teosinte (*Zea nicaraguensis* Iltis et Benz) as estimated by microsatellite variation	Genetic Resources & Crop Evolution	Article	0925-9864	Oct 2011	58	7	1021 1028	10.1007/s10722-010-9637-6
666	Ueno, S et al.	Generation of expressed sequence tags, development of microsatellite and single nucleotide polymorphism markers in *Primula sieboldii* E. Morren (Primulaceae) for analysis of genetic diversity in natural and horticultural populations	Breeding Science	Article	1344-7610	Sep 2011	61	3	234 243	10.1270/jsbbs.61.234
667	de Bang, TC et al.	A multiplex microsatellite marker kit for diversity assessment of large cassava (*Manihot esculenta* Crantz) germplasm collections	Plant Molecular Biology Reporter	Article	0735-9640	Sep 2011	29	3	655 662	10.1007/s11105-010-0273-2
668	Mirbabaee, SA et al.	Development of new microsatellite markers from an enriched genomic library of date palm (*Phoenix dactylifera* L.)	Journal of Horticultural Science & Biotechnology	Article	1462-0316	Sep 2011	86	5	539 541	NA
669	Baldauf, C et al.	Characterization of microsatellite loci in *Himatanthus drasticus* (Apocynaceae), a medicinal plant from the Brazilian savanna	American Journal of Botany	Article	0002-9122	Sep 2011	98	9	E244 E246	10.3732/ajb.1100135
670	Hopkins, SE; Taylor, DL	Microsatellite loci development in mycoheterotrophic *Corallorhiza maculata* (Orchidaceae) with amplification in *C. mertensiana*	American Journal of Botany	Article	0002-9122	Sep 2011	98	9	E253 E255	10.3732/ajb.1100061
671	Jiang, K et al.	A set of microsatellite primers for *Zostera japonica* (Zosteraceae)	American Journal of Botany	Article	0002-9122	Sep 2011	98	9	E236 E238	10.3732/ajb.1100296
672	Lu, ZL et al.	Isolation and characterization of 19 new microsatellite loci in *Colocasia esculenta* (Araceae)	American Journal of Botany	Article	0002-9122	Sep 2011	98	9	E239 E241	10.3732/ajb.1100067
673	Lucio, CCF et al.	Characterization of 12 microsatellite loci for *Hypochaeris chillensis* (Asteraceae) and cross-amplification in related species	American Journal of Botany	Article	0002-9122	Sep 2011	98	9	E262 E264	10.3732/ajb.1100177
674	Sun, J et al.	Development and characterization of 10 microsatellite loci in *Paeonia lactiflora* (Paeoniaceae)	American Journal of Botany	Article	0002-9122	Sep 2011	98	9	E242 E243	10.3732/ajb.1100083
675	Yu, XQ; Li, QM	Isolation and characterization of microsatellite markers for a worldwide invasive weed, *Chromolaena odorata* (Asteraceae)	American Journal of Botany	Article	0002-9122	Sep 2011	98	9	E259 E261	10.3732/ajb.1100169
676	Zhang, B et al.	Microsatellite markers for *Dayaoshania cotinifolia* (Gesneriaceae), a critically endangered perennial herb	American Journal of Botany	Article	0002-9122	Sep 2011	98	9	E256 E258	10.3732/ajb.1100170
677	Zhao, LL et al.	Development and characterization of microsatellite markers in the critically endangered species *Acer yangbiense* (Aceraceae)	American Journal of Botany	Article	0002-9122	Sep 2011	98	9	E247 E249	10.3732/ajb.1100142
678	Ganopoulos, IV et al.	Genetic diversity, structure and fruit trait associations in Greek sweet cherry cultivars using microsatellite based (SSR/ISSR) and morpho-physiological markers	Euphytica	Article	0014-2336	Sep 2011	181	2	237 251	10.1007/s10681-011-0416-z
679	Elmasulu, S et al.	Classification of 63 *Origanum* taxa based on microsatellite markers and essential oil composition	Planta Medica	Meeting Abstract	0032-0943	Aug 2011	77	12	1296 1296	NA
680	Ince, AG et al.	Transferability of EST-microsatellite markers to some *Labiatae* genera	Planta Medica	Meeting Abstract	0032-0943	Aug 2011	77	12	1360 1360	NA
681	Echt, CS et al.	Microsatellite DNA in genomic survey sequences and unigenes of loblolly pine	Tree Genetics & Genomes	Article	1614-2942	Aug 2011	7	4	773 780	10.1007/s11295-011-0373-7

	Author	Publication title	Source	Publication type	ISSN	Publication date	Vol	Issue	Pages	DOI
682	Fouet, O et al.	Structural characterization and mapping of functional EST-SSR markers in *Theobroma cacao*	Tree Genetics & Genomes	Article	1614-2942	Aug 2011	7	4	799 817	10.1007/s11295-011-0375-5
683	Chen, HK et al.	Development and characterization of polymorphic microsatellite primers in *Reaumuria soongorica* (Tamaricaceae)	American Journal of Botany	Article	0002-9122	Aug 2011	98	8	E221 E223	10.3732/ajb.1100112
684	Guan, BC et al.	Development and characterization of polymorphic microsatellite markers in *Dysosma pleiantha* (Berberidaceae)	American Journal of Botany	Article	0002-9122	Aug 2011	98	8	E210 E212	10.3732/ajb.1100107
685	Guan, LH et al.	Isolation and characterization of tetranucleotide microsatellite loci in *Pinus massoniana* (Pinaceae)	American Journal of Botany	Article	0002-9122	Aug 2011	98	8	E216 E217	10.3732/ajb.1100076
686	Ho, CW et al.	Development of 12 genic microsatellite loci for a biofuel grass, *Miscanthus sinensis* (Poaceae)	American Journal of Botany	Article	0002-9122	Aug 2011	98	8	E201 E203	10.3732/ajb.1100071
687	Imanishi, A et al.	Development of microsatellite markers for *Euryale ferox* (Nymphaeaceae), an endangered aquatic plant species in japan	American Journal of Botany	Article	0002-9122	Aug 2011	98	8	E233 E235	10.3732/ajb.1100056
688	Li, LF et al.	Genomic and est microsatellite markers for *Aquilegia flabellata* and cross-amplification in *A. oxysepala* (Ranunculaceae)	American Journal of Botany	Article	0002-9122	Aug 2011	98	8	E213 E215	10.3732/ajb.1100057
689	Liu, H et al.	Development and characterization of microsatellite markers for *Panax notoginseng* (Araliaceae), a chinese traditional herb	American Journal of Botany	Article	0002-9122	Aug 2011	98	8	E218 E220	10.3732/ajb.1100043
690	Pan, HW et al.	Development of microsatellite loci for *Cephalotaxus oliveri* (Cephalotaxaceae) and cross-amplification in *Cephalotaxus*	American Journal of Botany	Article	0002-9122	Aug 2011	98	8	E229 E232	10.3732/ajb.1100128
691	Perez, MF et al.	Isolation, characterization, and cross-species amplification of polymorphic microsatellite markers for *Pilosocereus machrisii* (Cactaceae)	American Journal of Botany	Article	0002-9122	Aug 2011	98	8	E204 E206	10.3732/ajb.1100033
692	Xu, W et al.	Microsatellite marker development in tung trees (*Vernicia montana* and *V. fordii*, Euphorbiaceae)	American Journal of Botany	Article	0002-9122	Aug 2011	98	8	E226 E228	10.3732/ajb.1100151
693	Yu, HY et al.	Development of polymorphic microsatellite markers for *Incarvillea sinensis* (Bignoniaceae)	American Journal of Botany	Article	0002-9122	Aug 2011	98	8	E224 E225	10.3732/ajb.1100052
694	Jakse, J et al.	Development of transcript-associated microsatellite markers for diversity and linkage mapping studies in hop (*Humulus lupulus* L.)	Molecular Breeding	Article	1380-3743	Aug 2011	28	2	227 239	10.1007/s11032-010-9476-3
695	Motilal, LA et al.	Microsatellite fingerprinting in the International Cocoa Genebank, Trinidad: accession and plot homogeneity information for germplasm management	Plant Genetic Resources	Article	1479-2621	Aug 2011	9	3	430 438	10.1017/S147926211100058X
696	Rodriguez-Suarez, C et al.	Applicability of chromosome-specific SSR wheat markers for the introgression of *Triticum urartu* in durum wheat breeding programmes	Plant Genetic Resources	Article	1479-2621	Aug 2011	9	3	439 444	10.1017/S147926211100061X
697	Khierallah, HSM et al.	Genetic diversity of Iraqi date palms revealed by microsatellite polymorphism	Journal of the American Society for Horticultural Science	Article	0003-1062	Jul 2011	136	4	282 287	NA
698	Cao, Y et al.	Evaluation of genetic identity and variation in cultivars of *Pyrus pyrifolia* (Burm.f.) Nakai from China using microsatellite markers	Journal of Horticultural Science & Biotechnology	Article	1462-0316	Jul 2011	86	4	331 336	NA
699	Caullet, CML et al.	Development of microsatellite markers in *Capsella* rubella and *Capsella bursa-pastoris* (Brassicaceae)	American Journal of Botany	Article	0002-9122	Jul 2011	98	7	E176 E179	10.3732/ajb.1100081
700	Corral, JM et al.	Isolation and characterization of microsatellite loci from apomictic *Hypericum perforatum* (Hypericaceae)	American Journal of Botany	Article	0002-9122	Jul 2011	98	7	E167 E169	10.3732/ajb.1100059
701	Covarrubias, S et al.	Isolation and characterization of microsatellite markers in *Distylous palicourea padifolia* (Rubiaceae)	American Journal of Botany	Article	0002-9122	Jul 2011	98	7	E164 E166	10.3732/ajb.1100042
702	Lee, DH et al.	Isolation and characterization of 10 microsatellite loci from Korean *Leontopodium japonicum* (Asteraceae)	American Journal of Botany	Article	0002-9122	Jul 2011	98	7	E183 E184	10.3732/ajb.1100065
703	Matakis, S et al.	Isolation and characterization of microsatellite markers for *Bothriochloa ischaemum* (Poaceae)	American Journal of Botany	Article	0002-9122	Jul 2011	98	7	E192 E194	10.3732/ajb.1100102
704	Matesanz, S et al.	Development and characterization of microsatellite markers for *Polygonum cespitosum* (Polygonaceae)	American Journal of Botany	Article	0002-9122	Jul 2011	98	7	E180 E182	10.3732/ajb.1100053
705	Nazareno, AG et al.	Microsatellite markers for *Butia eriospatha* (Arecaceae), a vulnerable palm species from the atlantic rainforest of Brazil	American Journal of Botany	Article	0002-9122	Jul 2011	98	7	E198 E200	10.3732/ajb.1100064
706	Zhang, J et al.	Development and polymorphism of microsatellite primers in *Ficus* pumila L. (Moraceae)	American Journal of Botany	Article	0002-9122	Jul 2011	98	7	E170 E172	10.3732/ajb.1000340
707	Zhang, ZR et al.	A set of novel microsatellite markers developed for	American Journal of Botany	Article	0002-9122	Jul 2011	98	7	E173 E175	10.3732/ajb.1000534

	Author	Publication title	Source	Publication type	ISSN	Publication date	Vol	Issue	Pages	DOI
		the traditional Tibetan medicinal plant *Halenia elliptica* (Gentianaceae)								
708	Zhou, HF et al.	Development of microsatellite markers for *Miscanthus sinensis* (Poaceae) and cross-amplification in other related species	American Journal of Botany	Article	0002-9122	Jul 2011	98	7	E195 E197	10.3732/ajb.1100040
709	Gadaleta, A et al.	Comparison of genomic and EST-derived SSR markers in phylogenetic analysis of wheat	Plant Genetic Resources	Article	1479-2621	Jul 2011	9	2	243 246	10.1017/S147926211100030X
710	Bindler, G et al.	A high density genetic map of tobacco (*Nicotiana tabacum* L.) obtained from large scale microsatellite marker development	Theoretical & Applied Genetics	Article	0040-5752	Jul 2011	123	2	219 230	10.1007/s00122-011-1578-8
711	Moreno-Sanz, P et al.	Microsatellite characterization of grapevine (*Vitis vinifera* L.) genetic diversity in Asturias (Northern Spain)	Scientia Horticulturae	Article	0304-4238	Jun 2011	129	3	433 440	10.1016/j.scienta.2011.04.013
712	Ge, Y et al.	Development and linkage mapping of unigene-derived microsatellite markers in *Brassica rapa* L.	Breeding Science	Article	1344-7610	Jun 2011	61	2	160 167	10.1270/jsbbs.61.160
713	Cupertino, FB et al.	Genetic diversity of *Eucalyptus* hybrids estimated by genomic and EST microsatellite markers	Biologia Plantarum	Article	0006-3134	Jun 2011	55	2	379 382	NA
714	Phumichai, C	Isolation of 55 microsatellite markers for *Jatropha curcas* and its closely related species	Biologia Plantarum	Article	0006-3134	Jun 2011	55	2	387 390	NA
715	Ruas, EA et al.	Isolation and characterization of eleven polymorphic microsatellite loci in *Aegiphila sellowiana* and their transferability	Biologia Plantarum	Article	0006-3134	Jun 2011	55	2	396 399	NA
716	Pil, MW et al.	Postglacial North-South expansion of populations of *Rhizophora mangle* (Rhizophoraceae) along the brazilian coast revealed by microsatellite analysis	American Journal of Botany	Article	0002-9122	Jun 2011	98	6	1031 1039	10.3732/ajb.1000392
717	Chen, JH et al.	Isolation and characterization of 20 new microsatellite loci in *Coriaria nepalensis* (Coriariaceae)	American Journal of Botany	Article	0002-9122	Jun 2011	98	6	E141 E143	10.3732/ajb.1100001
718	Dao, ZL et al.	Development of ten polymorphic microsatellite loci for *Fosbergia shweliensis* (Rubiaceae), a potentially crisis endangered tree	American Journal of Botany	Article	0002-9122	Jun 2011	98	6	E161 E163	10.3732/ajb.1100017
719	Jiang, K et al.	Microsatellite primers for vulnerable seagrass *Halophila beccarii* (Hydrocharitaceae)	American Journal of Botany	Article	0002-9122	Jun 2011	98	6	E155 E157	10.3732/ajb.1100032
720	Lin, YF et al.	Development of microsatellite markers in *Kmeria septentrionalis* (Magnoliaceae), an endangered chinese tree	American Journal of Botany	Article	0002-9122	Jun 2011	98	6	E158 E160	10.3732/ajb.1100039
721	Liu, WS et al.	Microsatellite primers in *Stipa purpurea* (Poaceae), a dominant species of the steppe on the Qinghai-Tibetan Plateau	American Journal of Botany	Article	0002-9122	Jun 2011	98	6	E150 E151	10.3732/ajb.1000444
722	Ohsako, T; Yamada, Y	Isolation and characterization of microsatellite loci in *Schoenoplectus juncoides* (Cyperaceae)	American Journal of Botany	Article	0002-9122	Jun 2011	98	6	E147 E149	10.3732/ajb.1100011
723	Sharma, H et al.	Identification and cross-species transferability of 112 novel unigene-derived microsatellite markers in tea (*Camellia sinensis*)	American Journal of Botany	Article	0002-9122	Jun 2011	98	6	E133 E138	10.3732/ajb.1000525
724	Siqueira, MVBM et al.	New microsatellite loci for water yam (*Dioscorea alata*, Dioscoreaceae) and cross-amplification for other *Dioscorea* species	American Journal of Botany	Article	0002-9122	Jun 2011	98	6	E144 E146	10.3732/ajb.1000513
725	Zhu, L et al.	Isolation and characterization of microsatellite primers for an invasive weed, *Solanum rostratum* (Solanaceae)	American Journal of Botany	Article	0002-9122	Jun 2011	98	6	E152 E154	10.3732/ajb.1100020
726	Narshimulu, G et al.	Potentiality of evenly distributed hypervariable microsatellite markers in marker-assisted breeding of rice	Plant Breeding	Article	0179-9541	Jun 2011	130	3	314 320	10.1111/j.1439-0523.2010.01834.x
727	Vargas, A et al.	Development and use of microsatellite markers for genetic diversity analysis of canahua (*Chenopodium pallidicaule* Aellen)	Genetic Resources & Crop Evolution	Article	0925-9864	Jun 2011	58	5	727 739	10.1007/s10722-010-9615-z
728	Khar, A et al.	Microsatellite marker based analysis of genetic diversity in short day tropical Indian onion and cross amplification in related *Allium* spp.	Genetic Resources & Crop Evolution	Article	0925-9864	Jun 2011	58	5	741 752	10.1007/s10722-010-9616-y
729	Cristofani-Yaly, M et al.	Transferability and level of heterozygosity of microsatellite markers in *Citrus* species	Plant Molecular Biology Reporter	Article	0735-9640	Jun 2011	29	2	418 423	10.1007/s11105-010-0241-x
730	Wang, SA et al.	Genetic diversity in *Apium graveolens* and related species revealed by SRAP and SSR markers	Scientia Horticulturae	Article	0304-4238	May 2011	129	1	1 8	10.1016/j.scienta.2011.03.020
731	Xu, W et al.	Development of novel chloroplast microsatellite markers for *Dendrobium officinale*, and cross-	Scientia Horticulturae	Article	0304-4238	May 2011	128	4	485 489	10.1016/j.scienta.2011.02.016

	Author	Publication title	Source	Publication type	ISSN	Publication date		Vol	Issue	Pages		DOI
		amplification in other *Dendrobium* species (Orchidaceae)										
732	Kaushik, A et al.	Phylogenetic relationships among various groups of rice (*Oryza sativa* L.) as revealed by microsatellite and transposable element-based marker analysis	Indian Journal Of Genetics & Plant Breeding	Article	0019-5200	May	2011	71	2	139	150	NA
733	Ang, CC et al.	Isolation and characterization of microsatellite loci in an endangered palm, *Johannesteijsmannia lanceolata* (Arecaceae)	American Journal of Botany	Article	0002-9122	May	2011	98	5	E117	E119	10.3732/ajb.1000494
734	Buehler, D et al.	Using the 454 pyrosequencing-based technique in the development of nuclear microsatellite loci in the alpine plant *Arabis alpina* (Brassicaceae)	American Journal of Botany	Article	0002-9122	May	2011	98	5	E103	E105	10.3732/ajb.1000488
735	Delmas, CEL et al.	Isolation and characterization of microsatellite loci in *Rhododendron ferrugineum* (Ericaceae) using pyrosequencing technology	American Journal of Botany	Article	0002-9122	May	2011	98	5	E120	E122	10.3732/ajb.1000533
736	Michalczyk, IM et al.	Identification and characterization of 12 microsatellite loci in *Cnidium dubium* (Apiaceae) using next-generation sequencing	American Journal of Botany	Article	0002-9122	May	2011	98	5	E127	E129	10.3732/ajb.1000429
737	Perez, F et al.	Microsatellite markers for the high Andean species *Schizanthus hookeri* and *S. grahamii* (Solanaceae)	American Journal of Botany	Article	0002-9122	May	2011	98	5	E114	E116	10.3732/ajb.1000487
738	Tnah, LH et al.	Microsatellite markers of an important medicinal plant, *Eurycoma longifolia* (Simaroubaceae), for DNA profiling	American Journal of Botany	Article	0002-9122	May	2011	98	5	E130	E132	10.3732/ajb.1000469
739	Yang, AH et al.	Chloroplast microsatellite markers in *Liriodendron tulipifera* (Magnoliaceae) and cross-species amplification in *L. chinense*	American Journal of Botany	Article	0002-9122	May	2011	98	5	E123	E126	10.3732/ajb.1000532
740	Korbecka, G et al.	Mixed mating in androdioecious *Mercurialis annua* inferred using progeny arrays and diploid-acting microsatellite loci in a hexaploid background	Annals of Botany	Article	0305-7364	May	2011	107	6	1057	1061	10.1093/aob/mcr028
741	Dunbar-Co, S et al.	Genetic structure among populations in the endemic Hawaiian *Plantago* lineage: insights from microsatellite variation	Plant Species Biology	Article	0913-557X	May	2011	26	2	134	144	10.1111/j.1442-1984.2011.00315.x
742	Arabnezhad, H et al.	Evaluation of genetic relationships among Iranian pistachios using microsatellite markers developed from *Pistacia khinjuk* Stocks	Scientia Horticulturae	Article	0304-4238	Apr	2011	128	3	249	254	10.1016/j.scienta.2011.01.028
743	Abreu, AG et al.	Development of microsatellite markers for *Aulonemia aristulata* (Poaceae) and cross-amplification in other bamboo species	American Journal of Botany	Article	0002-9122	Apr	2011	98	4	E90	E92	10.3732/ajb.1000511
744	Bajay, MM et al.	Development of a novel set of microsatellite markers for castor bean, *Ricinus communis* (Euphorbiaceae)	American Journal of Botany	Article	0002-9122	Apr	2011	98	4	E87	E89	10.3732/ajb.1000395
745	Benoit, L et al.	Polymorphic microsatellite loci from *Dacryodes edulis* (Burseraceae), a Central African rainforest and fruit-tree species	American Journal of Botany	Article	0002-9122	Apr	2011	98	4	E74	E75	10.3732/ajb.1000463
746	Huang, JL et al.	Isolation and characterization of 15 microsatellite markers from the spring orchid (*Cymbidium goeringii*) (Orchidaceae)	American Journal of Botany	Article	0002-9122	Apr	2011	98	4	E76	E77	10.3732/ajb.1000446
747	Ju, LP et al.	Microsatellite primers in the native perennial cycad *Cycas taitungensis* (Cycadaceae)	American Journal of Botany	Article	0002-9122	Apr	2011	98	4	E84	E86	10.3732/ajb.1000504
748	Liu, J et al.	Cross-species amplification and development of new microsatellite loci for *Taxus wallichiana* (Taxaceae)	American Journal of Botany	Article	0002-9122	Apr	2011	98	4	E70	E73	10.3732/ajb.1000445
749	Ravishankar, KV et al.	Development of new microsatellite markers from mango (*Mangifera indica*) and cross-species amplification	American Journal of Botany	Article	0002-9122	Apr	2011	98	4	E96	E99	10.3732/ajb.1000263
750	Waycott, M et al.	Microsatellite markers in the australian desert plant, *Solanum centrale* (Solanaceae)	American Journal of Botany	Article	0002-9122	Apr	2011	98	4	E81	E83	10.3732/ajb.1000356
751	Xie, XB et al.	Microsatellite primers in red bayberry, *Myrica rubra* (Myricaceae)	American Journal of Botany	Article	0002-9122	Apr	2011	98	4	E93	E95	10.3732/ajb.1000271
752	Hamdan, YAS et al.	Development and characterization of genomic microsatellite markers in safflower (*Carthamus tinctorius* L.)	Plant Breeding	Article	0179-9541	Apr	2011	130	2	237	241	10.1111/j.1439-0523.2010.01826.x
753	Le Guen, V et al.	Development and characterization of 296 new polymorphic microsatellite markers for rubber tree (*Hevea brasiliensis*)	Plant Breeding	Article	0179-9541	Apr	2011	130	2	294	296	10.1111/j.1439-0523.2010.01774.x
754	Hammadi, H et al.	Microsatellite diversity among Tunisian date palm	Pakistan Journal of Botany	Article	0556-3321	Apr	2011	43	2	1257	1264	NA

	Author	Publication title	Source	Publication type	ISSN	Publication date	Vol	Issue	Pages	DOI
		(*Phoenix dactylifera* L.) subpopulations								
755	Marwan et al.	Genetic diversity of selected chickpea elite lines and their progenitors based on microsatellite markers	Canadian Journal of Plant Science	Meeting Abstract	0008-4220	Mar 2011	91	2	401 401	NA
756	Kisha, TJ; Cramer, CS	Determining redundancy of short-day onion accessions in a germplasm collection using microsatellite and targeted region amplified polymorphic markers	Journal of the American Society For Horticultural Science	Article	0003-1062	Mar 2011	136	2	129 134	NA
757	Flatz, R et al.	Characterization of microsatellite loci in *Yucca brevifolia* (Agavaceae) and cross-amplification in related species	American Journal of Botany	Article	0002-9122	Mar 2011	98	3	E67 E69	10.3732/ajb.1000468
758	Flores-Renteria, L; Whipple, AV	A new approach to improve the scoring of mononucleotide microsatellite loci	American Journal of Botany	Article	0002-9122	Mar 2011	98	3	E51 E53	10.3732/ajb.1000428
759	Grmain-Aubrey, CC et al.	Microsatellite marker development for the federally listed *Prunus geniculata* (Rosaceae)	American Journal of Botany	Article	0002-9122	Mar 2011	98	3	E58 E60	10.3732/ajb.1000435
760	Liao, H et al.	Microsatellite markers in the traditional Chinese medicinal herb *Gynostemma pentaphyllum* (Cucurbitaceae)	American Journal of Botany	Article	0002-9122	Mar 2011	98	3	E61 E63	10.3732/ajb.1000456
761	Servick, SV et al.	Microsatellite marker development for *Galax urceolata* (Diapensiaceae)	American Journal of Botany	Article	0002-9122	Mar 2011	98	3	E48 E50	10.3732/ajb.1000427
762	Wang, B et al.	Microsatellite loci in *Vallisneria natans* (Hydrocharitaceae) and cross-reactivity with *V. spinulosa* and *V. denseserrulata*	American Journal of Botany	Article	0002-9122	Mar 2011	98	3	E44 E47	10.3732/ajb.1000441
763	Xu, XH et al.	Solation of compound microsatellite markers for the common mediterranean shrub *Smilax aspera* (Smilacaceae)	American Journal of Botany	Article	0002-9122	Mar 2011	98	3	E64 E66	10.3732/ajb.1000447
764	He, QA et al.	Genetic diversity and identity of Chinese loquat cultivars/accessions (*Eriobotrya japonica*) using apple SSR markers	Plant Molecular Biology Reporter	Article	0735-9640	Mar 2011	29	1	197 208	10.1007/s11105-010-0218-9
765	Wang, YW et al.	Development of 1,030 genomic SSR markers in switchgrass	Theoretical & Applied Genetics	Article	0040-5752	Mar 2011	122	4	677 686	10.1007/s00122-010-1477-4
766	Robson, PRH et al.	A flexible quantitative methodology for the analysis of gene-flow between conventionally bred maize populations using microsatellite markers	Theoretical % Applied Genetics	Article	0040-5752	Mar 2011	122	4	819 829	10.1007/s00122-010-1489-0
767	Zhao, H et al.	Transferability of microsatellite markers from *Brachypodium distachyon* to *Miscanthus sinensis*, a potential biomass crop	Journal of Integrative Plant Biology	Article	1672-9072	Mar 2011	53	3	232 245	10.1111/j.1744-7909.2010.01026.x
768	Mutegi, E et al.	Genetic structure and relationships within and between cultivated and wild sorghum (*Sorghum bicolor* (L.) Moench) in Kenya as revealed by microsatellite markers	Theoretical & Applied Genetics	Article	0040-5752	Mar 2011	122	5	989 1004	10.1007/s00122-010-1504-5
769	Rajarajan, K; Ganesamurthy, K	Genetic diversity analysis of sorghum [*Sorghum bicolor* (L.) Moench] genotypes for drought tolerance using SSR markers	Indian Journal of Genetics & Plant Breeding	Article	0019-5200	Feb 2011	71	1	17 24	NA
770	Yadav, HK et al.	EST-derived SSR markers in *Jatropha curcas* L.: development, characterization, polymorphism, and transferability across the species/genera	Tree Genetics & Genomes	Article	1614-2942	Feb 2011	7	1	207 219	10.1007/s11295-010-0326-6
771	Singh, A et al.	Identification of microsatellite markers linked to leaf rust adult plant resistance (APR) gene *Lr48* in wheat	Plant Breeding	Article	0179-9541	Feb 2011	130	1	31 34	10.1111/j.1439-0523.2010.01820.x
772	Sousa, ACB et al.	Development of microsatellite markers in guineagrass (*Panicum maximum* Jacq.) and their transferability to other tropical forage grass species	Plant Breeding	Article	0179-9541	Feb 2011	130	1	104 108	10.1111/j.1439-0523.2010.01779.x
773	Dadkhodaie, NA et al.	Mapping genes *Lr53* and *Yr35* on the short arm of chromosome 6B of common wheat with microsatellite markers and studies of their association with *Lr36*	Theoretical & Applied Genetics	Article	0040-5752	Feb 2011	122	3	479 487	10.1007/s00122-010-1462-y
774	Glennon, KL; Church, SA	Microsatellite primers for the North American bluets (*Houstonia* section *Amphiotis*, Rubiaceae)	American Journal of Botany	Article	0002-9122	Feb 2011	98	2	E28 E29	10.3732/ajb.1000294
775	Li, L et al.	Microsatellite markers for the Chinese herbaceous peony *Paeonia lactiflora* (Paeoniaceae)	American Journal of Botany	Article	0002-9122	Feb 2011	98	2	E16 E18	10.3732/ajb.1000410
776	Ma, Y et al.	Development and characterization of 21 est-derived microsatellite markers in *Vicia faba* (fava bean)	American Journal of Botany	Article	0002-9122	Feb 2011	98	2	E22 E24	10.3732/ajb.1000407
777	Moe, AM; Weiblen, GD	Development and characterization of microsatellite loci in dioecious figs (*Ficus*, Moraceae)	American Journal of Botany	Article	0002-9122	Feb 2011	98	2	E25 E27	10.3732/ajb.1000412
778	Nunez-Avila, MC et al.	Microsatellite markers for the relict tree *Aextoxicon*	American Journal of Botany	Article	0002-9122	Feb 2011	98	2	E30 E32	10.3732/ajb.1000425

	Author	Publication title	Source	Publication type	ISSN	Publication date		Vol	Issue	Pages		DOI
		punctatum: the only species in the Chilean endemic family Aextoxicaceae										
779	Schreiter, S et al.	Polymorphic microsatellite markers in the invasive shrub *Buddleja davidii* (Scrophulariaceae)	American Journal of Botany	Article	0002-9122	Feb	2011	98	2	E39	E40	10.3732/ajb.1000417
780	Shi, YS et al.	Development and characterization of polymorphic microsatellite markers in *Castanopsis sclerophylla* (Fagaceae)	American Journal of Botany	Article	0002-9122	Feb	2011	98	2	E19	E21	10.3732/ajb.1000400
781	Wu, ZH et al.	Development and characterization of microsatellite markers for *Sagittaria trifolia* var. *sinensis* (Alismataceae)	American Journal of Botany	Article	0002-9122	Feb	2011	98	2	E36	E38	10.3732/ajb.1000434
782	Kalia, RK et al.	Microsatellite markers: an overview of the recent progress in plants	Euphytica	Review	0014-2336	Feb	2011	177	3	309	334	10.1007/s10681-010-0286-9
783	Marconi, G et al.	Primer Note: Microsatellite-AFLP development for *Araucaria araucana* (Mol.) K. Koch, an endangered conifer of Chilean and Argentinean native forests	Silvae Genetica	Article	0037-5349	NA	2011	60	6	285	288	NA
784	Saddoud, O et al.	Using morphological characters and simple sequence repeat (SSR) markers to characterize Tunisian fig (*Ficus carica* L.) cultivars	Acta Biologica Cracoviensia Series Botanica	Article	0001-5296	NA	2011	53	2	7	14	10.2478/v10182-011-0019-y
785	Adeyemo, O et al.	Genetic diversity assessment and relationship among tropical-yellow endosperm maize inbred lines using SSR markers	Maydica	Article	0025-6153	NA	2011	56	1	43	49	NA
786	Lembicz, M et al.	Microsatellite identification of ramet genotypes in a clonal plant with phalanx growth: The case of *Cirsium rivulare* (Asteraceae)	Flora	Article	0367-2530	NA	2011	206	9	792	798	10.1016/j.flora.2011.04.006
787	Iwaizumi, MG et al.	Primer Note: Development of highly polymorphic nuclear microsatellite markers for hinoki (*Chamaecyparis obtusa*)	Silvae Genetica	Article	0037-5349	NA	2011	60	2	62	65	NA
788	Ginwal, HS et al.	Short Note: Cross-species amplification and characterization of pinus chloroplast microsatellite markers in *Cedrus deodara* Roxb.	Silvae Genetica	Article	0037-5349	NA	2011	60	2	65	69	NA
789	Feng, J et al.	Identification of microsatellite markers linked to quantitative trait loci controlling resistance to *Fusarium* root rot in field pea	Canadian Journal of Plant Science	Article	0008-4220	Jan	2011	91	1	199	204	10.4141/CJPS09176
790	Soriano, JM et al.	Development and characterization of microsatellite markers in pomegranate (*Punica granatum* L.)	Molecular Breeding	Article	1380-3743	Jan	2011	27	1	119	128	10.1007/s11032-010-9511-4
791	Kulbaba, MW; Worley, AC	Polymorphic microsatellite loci in *Polemonium brandegei* and *P. viscosum* (section *Melliosoma*, Polemoniaceae)	American Journal of Botany	Article	0002-9122	Jan	2011	98	1	E4	E6	10.3732/ajb.1000365
792	Pereira, MF et al.	Isolation and characterization of microsatellite loci in *Cabralea canjerana* (Meliaceae)	American Journal of Botany	Article	0002-9122	Jan	2011	98	1	E10	E12	10.3732/ajb.1000336
793	Xu, TT et al.	Development of microsatellite loci for *Aconitum gymnandrum* (Ranunculaceae), a species endemic to the Qinghai-Tibetan Plateau	American Journal of Botany	Article	0002-9122	Jan	2011	98	1	E7	E9	10.3732/ajb.1000418
794	Christelova, P et al.	A platform for efficient genotyping in *Musa* using microsatellite markers	AoB Plants	Article	2041-2851	NA	2011	NA	NA	NA		10.1093/aobpla/plr024
795	Montero-Rojas, M et al.	Molecular differentiation and diversity of cassava (*Manihot esculenta*) taken from 162 locations across Puerto Rico and assessed with microsatellite markers	AoB Plants	Article	2041-2851	NA	2011	NA	NA	NA		10.1093/aobpla/plr010
796	Huang, Z et al.	AFLP and SSR markers linked to the yellow seed colour gene in *Brassica juncea* L.	Czech Journal of Genetics & Plant Breeding	Article	1212-1975	NA	2011	47	4	149	155	NA
797	Golabadi, M et al.	Identification of microsatellite markers linked with yield components under drought stress at terminal growth stages in durum wheat	Euphytica	Article	0014-2336	Jan	2011	177	2	207	221	10.1007/s10681-010-0242-8
798	Takahashi, Y et al.	Comparison of genetic variation and differentiation using microsatellite markers among three rare threatened and one widespread toad lily species of *Tricyrtis* section Flavae (Convallariaceae) in Japan	Plant Species Biology	Article	0913-557X	Jan	2011	26	1	13	23	10.1111/j.1442-1984.2010.00297.x
799	Bassil, NV et al.	Quince (*Cydonia oblonga*) genetic relationships determined using microsatellite markers	XI International Pear Symposiumse, Acta Horticulturae	Proceedings Paper	0567-7572BN 978-90-66055-04-9	Nov	2011	909	NA	75	83	NA
800	Dossett, M et al.	High resolution melting detects sequence polymorphism in *Rubus occidentalis* monomorphic microsatellite markers	IHC2010: International Symposium on Berries: From Genomics to Sustainable	Proceedings Paper	0567-7572BN 978-90-66056-84-8	Aug	2011	926	NA	91	95	NA

	Author	Publication title	Source	Publication type	ISSN	Publication date	Vol	Issue	Pages	DOI
			Production, Quality and Health, Acta Horticulturae							
801	Qiu, LJ et al.	Exploiting EST databases for the development and characterization of EST-SSR markers in castor bean (*Ricinus communis* L.)	BMC Plant Biology	Article	1471-2229	Dec 2010	10	NA	NA NA	10.1186/1471-2229-10-278
802	Sun, Y et al.	Ten polymorphic microsatellite markers in *Michelia maudiae* (Magnoliaceae)	American Journal of Botany	Article	0002-9122	Dec 2010	97	12	E157 E158	10.3732/ajb.1000332
803	Wang, HW et al.	Development and characterization of microsatellite loci in *Taihangia rupestris* (Rosaceae), a rare cliff herb	American Journal of Botany	Article	0002-9122	Dec 2010	97	12	E136 E138	10.3732/ajb.1000334
804	Zhai, SN et al.	Isolation of compound microsatellite markers for the endangered plant *Neolitsea sericea* (Lauraceae)	American Journal of Botany	Article	0002-9122	Dec 2010	97	12	E139 E141	10.3732/ajb.1000348
805	Avramidou, E et al.	DNA fingerprinting of elite Greek wild cherry (*Prunus avium* L.) genotypes using microsatellite markers	Forestry	Article	0015-752X	Dec 2010	83	5	527 533	10.1093/forestry/cpq035
806	Gavrilenko, T et al.	A microsatellite and morphological assessment of the Russian National cultivated potato collection	Genetic Resources & Crop Evolution	Article	0925-9864	Dec 2010	57	8	1151 1164	10.1007/s10722-010-9554-8
807	Campoy, JA et al.	Developing microsatellite multiplex and megaplex PCR systems for high-throughput characterization of breeding progenies and linkage maps spanning the apricot (*Prunus armeciaca* L.) genome	Plant Molecular Biology Reporter	Article	0735-9640	Dec 2010	28	4	560 568	10.1007/s11105-010-0186-0
808	Caruso, M et al.	Microsatellite markers help to assess genetic diversity among *Opuntia ficus indica* cultivated genotypes and their relation with related species	Plant Systematics & Evolution	Article	0378-2697	Dec 2010	290	1-4	85 97	10.1007/s00606-010-0351-9
809	Stewart, JF et al.	Microsatellite versus AFLP analyses of pre-management introgression levels in loblolly pine (*Pinus taeda* L.) and shortleaf pine (*P. echinata* Mill.)	Tree Genetics & Genomes	Article	1614-2942	Dec 2010	6	6	853 862	10.1007/s11295-010-0296-8
810	Castillo, A et al.	Genetic structure and ecogeographical adaptation in wild barley (*Hordeum chilense* Roemer et Schultes) as revealed by microsatellite markers	BMC Plant Biology	Article	1471-2229	Nov 2010	10	NA	NA	10.1186/1471-2229-10-266
811	Honig, JA et al.	Isolation and characterization of 88 polymorphic microsatellite markers in Kentucky bluegrass (*Poa pratensis* L.)	Hortscience	Article	0018-5345	Nov 2010	45	11	1759 1763	NA
812	Choo, J et al.	Characterization of 14 microsatellite loci in a tropical palm, *Attalea phalerata* (Arecaceae)	American Journal of Botany	Article	0002-9122	Nov 2010	97	11	E105 E106	10.3732/ajb.1000281
813	Cidade, FW et al.	Microsatellite loci for *Paspalum atratum* (Poaceae) and cross-amplification in other species	American Journal of Botany	Article	0002-9122	Nov 2010	97	11	E107 E110	10.3732/ajb.1000207
814	Sexton, GJ et al.	Development and characterization of microsatellite loci for *Khaya senegalensis* (Meliaceae)	American Journal of Botany	Article	0002-9122	Nov 2010	97	11	E111 E113	10.3732/ajb.1000300
815	Viruel, J et al.	New microsatellite loci in the dwarf yams *Dioscorea* group *epipetrum* (Dioscoreaceae)	American Journal of Botany	Article	0002-9122	Nov 2010	97	11	E121 E123	10.3732/ajb.1000304
816	Wang, ZS et al.	Isolation and characterization of 50 nuclear microsatellite markers for *Cathaya argyrophylla*, a Chinese endemic conifer	American Journal of Botany	Article	0002-9122	Nov 2010	97	11	E117 E120	10.3732/ajb.1000270
817	Wu, W et al.	Development of microsatellite loci for the invasive weed *Wedelia trilobata* (Asteraceae)	American Journal of Botany	Article	0002-9122	Nov 2010	97	11	E114 E116	10.3732/ajb.1000327
818	Dixit, A et al.	Development of new microsatellite markers for molecular diversity analysis of *Citrus* species	Journal of Horticultural Science & Biotechnology	Article	1462-0316	Nov 2010	85	6	521 527	NA
819	Golubov, A et al.	Microsatellite instability in *Arabidopsis* increases with plant development	Plant Physiology	Article	0032-0889	Nov 2010	154	3	1415 1427	10.1104/pp.110.162933
820	Hasan, NA et al.	Polymorphic chloroplast microsatellite markers in the octoploid *Lepidium meyenii* (Brassicaceae) and cross-species amplification in lepidium	American Journal of Botany	Article	0002-9122	Oct 2010	97	10	E85 E88	10.3732/ajb.1000225
821	Jones, BL et al.	Isolation and characterization of microsatellite loci in *Santalum lanceolatum* and *Santalum leptocladum* (Santalaceae)	American Journal of Botany	Article	0002-9122	Oct 2010	97	10	E97 E98	10.3732/ajb.1000213
822	Wei, JQ et al.	Isolation and characterization of polymorphic microsatellite loci in *Camellia nitidissima* Chi (Theaceae)	American Journal of Botany	Article	0002-9122	Oct 2010	97	10	E89 E90	10.3732/ajb.1000234
823	Zeng, LY et al.	Microsatellite markers for the cushion rock jasmine, *Androsace tapete* (Primulaceae), a species endemic to the Qinghai-Tibetan Plateau	American Journal of Botany	Article	0002-9122	Oct 2010	97	10	E94 E96	10.3732/ajb.1000260
824	Zhang, L; Li, QM	Isolation and characterization of microsatellite	American Journal of Botany	Article	0002-9122	Oct 2010	97	10	E91 E93	10.3732/ajb.1000245

	Author	Publication title	Source	Publication type	ISSN	Publication date		Vol	Issue	Pages		DOI
		markers in an endangered species *Dracaena cambodiana* (Liliaceae)										
825	Muraya, MM et al.	Investigation of recent population bottlenecks in Kenyan wild sorghum populations (*Sorghum bicolor* (L.) Moench ssp *verticilliflorum* (Steud.) De Wet) based on microsatellite diversity and genetic disequilibria	Genetic Resources & Crop Evolution	Article	0925-9864	Oct	2010	57	7	995	1005	10.1007/s10722-010-9539-7
826	Gurcan, K; Mehlenbacher, SA	Development of microsatellite marker loci for European hazelnut (*Corylus avellana* L.) from ISSR fragments	Molecular Breeding	Article	1380-3743	Oct	2010	26	3	551	559	10.1007/s11032-010-9464-7
827	Kiani, M et al.	Microsatellite analysis of Iranian Damask rose (*Rosa damascena* Mill.) germplasm	Plant Breeding	Article	0179-9541	Oct	2010	129	5	551	557	10.1111/j.1439-0523.2009.01708.x
828	Sigrist, MS et al.	Development and characterization of microsatellite markers for turmeric (Curcuma *longa*)	Plant Breeding	Article	0179-9541	Oct	2010	129	5	570	573	10.1111/j.1439-0523.2009.01720.x
829	Puschenreiter, M et al.	Differentiation of metallicolous and non-metallicolous *Salix caprea* populations based on phenotypic characteristics and nuclear microsatellite (SSR) markers	Plant Cell And Environment	Article	0140-7791	Oct	2010	33	10	1641	1655	10.1111/j.1365-3040.2010.02170.x
830	Prado, MJ et al.	Detection of somaclonal variants in somatic embryogenesis-regenerated plants of *Vitis vinifera* by flow cytometry and microsatellite markers	Plant Cell Tissue & Organ Culture	Article	0167-6857	Oct	2010	103	1	49	59	10.1007/s11240-010-9753-1
831	Naval, MD et al.	Analysis of genetic diversity among persimmon cultivars using microsatellite markers	Tree Genetics & Genomes	Article	1614-2942	Oct	2010	6	5	677	687	10.1007/s11295-010-0283-0
832	Mnejja, M et al.	*Prunus* microsatellite marker transferability across rosaceous crops	Tree Genetics & Genomes	Article	1614-2942	Oct	2010	6	5	689	700	10.1007/s11295-010-0284-z
833	Martin, MA et al.	Genetic diversity in European chestnut populations by means of genomic and genic microsatellite markers	Tree Genetics & Genomes	Article	1614-2942	Oct	2010	6	5	735	744	10.1007/s11295-010-0287-9
834	Muzzalupo, I et al.	Intra-cultivar variability of three major olive cultivars grown in different areas of Central-Southern Italy and studied using microsatellite markers	Scientia Horticulturae	Article	0304-4238	Sep	2010	126	3	324	329	10.1016/j.scienta.2010.07.014
835	Gasi, F et al.	Genetic assessment of apple germplasm in Bosnia and Herzegovina using microsatellite and morphologic markers	Scientia Horticulturae	Article	0304-4238	Sep	2010	126	2	164	171	10.1016/j.scienta.2010.07.002
836	Chen, C et al.	New microsatellite markers for the rare plant *Cercidiphyllum japonicum* and their utility for *Cercidiphyllum* magnificum	American Journal of Botany	Article	0002-9122	Sep	2010	97	9	E82	E84	10.3732/ajb.1000165
837	Ohsako, T et al.	Spatial structure of microsatellite variability within and among populations of wild radish *Raphanus sativus* L. var. *hortensis* Backer f. *raphanistroides* Makino (Brassicaceae) in Japan	Breeding Science	Article	1344-7610	Sep	2010	60	3	195	202	NA
838	Li, JQ et al.	Population structure and genetic diversity in elite sugar beet germplasm investigated with SSR markers	Euphytica	Article	0014-2336	Sep	2010	175	1	35	42	10.1007/s10681-010-0161-8
839	Garcia-Moreno, MJ et al.	Transferability of non-genic microsatellite and gene-based sunflower markers to safflower	Euphytica	Article	0014-2336	Sep	2010	175	2	145	150	10.1007/s10681-010-0139-6
840	Wang, XQ et al.	Cross-amplification and characterization of microsatellite loci for the genus *Rhododendron*	Hortscience	Article	0018-5345	Sep	2010	45	9	1394	1397	NA
841	Karimi, R et al.	Molecular characterization of persian walnut populations in Iran with microsatellite markers	Hortscience	Article	0018-5345	Sep	2010	45	9	1403	1406	NA
842	Matsumoto, A et al.	Genetic diversity and structure of natural fragmented *Chamaecyparis obtusa* populations as revealed by microsatellite markers	Journal of Plant Research	Article	0918-9440	Sep	2010	123	5	689	699	10.1007/s10265-009-0299-4
843	Miranda, C et al.	Genetic diversity and structure in a collection of ancient Spanish pear cultivars assessed by microsatellite markers	Journal of the American Society for Horticultural Science	Article	0003-1062	Sep	2010	135	5	428	437	NA
844	de Oliveira, EJ et al.	Polymorphism of microsatellite markers in papaya (*Carica papaya* L.)	Plant Molecular Biology Reporter	Article	0735-9640	Sep	2010	28	3	519	530	10.1007/s11105-010-0180-6
845	Burle, ML et al.	Microsatellite diversity and genetic structure among common bean (*Phaseolus vulgaris* L.) landraces in Brazil, a secondary center of diversity	Theoretical & Applied Genetics	Article	0040-5752	Sep	2010	121	5	801	813	10.1007/s00122-010-1350-5
846	Gonzalez, LBP et al.	Development of microsatellite markers in *Lupinus luteus* (Fabaceae) and cross-species amplification in other lupine species	American Journal of Botany	Article	0002-9122	Aug	2010	97	8	E72	E74	10.3732/ajb.1000170
847	Wang, SZ et al.	Development and characterization of polymorphic	American Journal of Botany	Article	0002-9122	Aug	2010	97	8	E75	E78	10.3732/ajb.1000153

	Author	Publication title	Source	Publication type	ISSN	Publication date	Vol	Issue	Pages	DOI
		microsatellite markers in *Momordica charantia* (Cucurbitaceae)								
848	Wolko, L et al.	Genetic diversity of European pear cultivars (*Pyrus communis* L.) and wild pear (*Pyrus pyraster* (L.) Burgsd.) inferred from microsatellite markers analysis	Genetic Resources & Crop Evolution	Article	0925-9864	Aug 2010	57	6	801 806	10.1007/s10722-010-9587-z
849	van Treuren, R et al.	Microsatellite genotyping of apple (*Malus* x *domestica* Borkh.) genetic resources in the Netherlands: application in collection management and variety identification	Genetic Resources & Crop Evolution	Article	0925-9864	Aug 2010	57	6	853 865	10.1007/s10722-009-9525-0
850	Li, LN et al.	Isolation and characterization of 10 polymorphic microsatellite loci in *Paphiopedilum concolor* (Batem.) Pfitzer (Orchidaceae) and cross-species amplification	Hortscience	Article	0018-5345	Aug 2010	45	8	1286 1287	NA
851	Saxena, RK et al.	Application of SSR markers for molecular characterization of hybrid parents and purity assessment of ICPH 2438 hybrid of pigeonpea [*Cajanus cajan* (L.) Millspaugh]	Molecular Breeding	Article	1380-3743	Aug 2010	26	2	371 380	10.1007/s11032-010-9459-4
852	Rabbani, MA et al.	Genetic analysis of Basmati and non-Basmati Pakistani rice (*Oryza sativa* L.) cultivars using microsatellite markers	Pakistan Journal of Botany	Article	0556-3321	Aug 2010	42	4	2551 2564	NA
853	Gurcan, K et al.	Genetic diversity in hazelnut (*Corylus avellana* L.) cultivars from Black Sea countries assessed using SSR markers	Plant Breeding	Article	0179-9541	Aug 2010	129	4	422 434	10.1111/j.1439-0523.2009.01753.x
854	Ince, AG et al.	The first report of microsatellite primer pairs for genetic studies in jojoba [*Simmondsia chinensis* (Link) Schneider]	Planta Medica	Meeting Abstract	0032-0943	Aug 2010	76	12	1201 1201	NA
855	Brito, G et al.	Assessment of genetic stability of two micropropagated wild olive species using flow cytometry and microsatellite markers	Trees-Structure & Function	Article	0931-1890	Aug 2010	24	4	723 732	10.1007/s00468-010-0442-9
856	Park, YH et al.	Rose (*Rosa hybrida* L.) EST-derived microsatellite markers and their transferability to strawberry (*Fragaria* spp.)	Scientia Horticulturae	Article	0304-4238	Jul 2010	125	4	733 739	10.1016/j.scienta.2010.05.012
857	Curro, S et al.	New microsatellite loci for pomegranate, *Punica granatum* (Lythraceae)	American Journal of Botany	Article	0002-9122	Jul 2010	97	7	E58 E60	10.3732/ajb.1000143
858	Figueira, GM et al.	A set of microsatellite markers for *Arrabidaea chica* (Bignoniaceae), a medicinal liana from the neotropics	American Journal of Botany	Article	0002-9122	Jul 2010	97	7	E63 E64	10.3732/ajb.1000145
859	Li, XY et al.	Ten microsatellite markers in endangered species *Sauvagesia rhodoleuca* (Ochnaceae)	American Journal of Botany	Article	0002-9122	Jul 2010	97	7	E61 E62	10.3732/ajb.1000136
860	Huang, PH et al.	Isolation and characterization of 13 microsatellite loci from *Pedicularis rex* (lousewort)	Hortscience	Article	0018-5345	Jul 2010	45	7	1129 1131	NA
861	Mu, HP et al.	Genetic variation of *Ardisia crenata* in south China revealed by nuclear microsatellite	Journal of Systematics & Evolution	Article	1674-4918	Jul 2010	48	4	279 285	10.1111/j.1759-6831.2010.00081.x
862	Blair, MW et al.	Genetic mapping of microsatellite markers around the arcelin bruchid resistance locus in common bean	Theoretical & Applied Genetics	Article	0040-5752	Jul 2010	121	2	393 402	10.1007/s00122-010-1318-5
863	Gurcan, K et al.	Development, characterization, segregation, and mapping of microsatellite markers for European hazelnut (*Corylus avellana* L.) from enriched genomic libraries and usefulness in genetic diversity studies	Tree Genetics & Genomes	Article	1614-2942	Jul 2010	6	4	513 531	10.1007/s11295-010-0269-y
864	Devakumar, K et al.	Microsatellite analysis of distinct coconut accessions from Agatti and Kavaratti Islands, Lakshadweep, India	Scientia Horticulturae	Article	0304-4238	Jun 2010	125	3	309 315	10.1016/j.scienta.2010.04.012
865	Caser, M et al.	Are *Rhododendron* hybrids distinguishable on the basis of morphology and microsatellite polymorphism?	Scientia Horticulturae	Article	0304-4238	Jun 2010	125	3	469 476	10.1016/j.scienta.2010.04.037
866	Vik, U et al.	Microsatellite markers show decreasing diversity but unchanged level of clonality in *Dryas octopetala* (Rosaceae) with increasing latitude	American Journal of Botany	Article	0002-9122	Jun 2010	97	6	988 997	10.3732/ajb.0900215
867	Homolka, A et al.	Microsatellite markers in the tree peony, *Paeonia suffruticosa* (Paeoniaceae)	American Journal of Botany	Article	0002-9122	Jun 2010	97	6	E42 E44	10.3732/ajb.1000127
868	Li, Y et al.	Development of microsatellite loci for *Pinus koraiensis* (Pinaceae)	American Journal of Botany	Article	0002-9122	Jun 2010	97	6	E39 E41	10.3732/ajb.1000098
869	Sun, Y et al.	Development, characterization, and transferability of microsatellite markers for *Kirengeshoma palmata* (Hydrangeaceae)	American Journal of Botany	Article	0002-9122	Jun 2010	97	6	E48 E51	10.3732/ajb.1000134

	Author	Publication title	Source	Publication type	ISSN	Publication date		Vol	Issue	Pages		DOI
870	Xu, NN et al.	Microsatellite primers for *Halophila ovalis* and cross-amplification in *H. minor* (Hydrocharitaceae)	American Journal of Botany	Article	0002-9122	Jun	2010	97	6	E56	E57	10.3732/ajb.1000111
871	Ma, KH et al.	Assessment of genetic diversity and relationships among *Coix lacryma*-jobi accessions using microsatellite markers	Biologia Plantarum	Article	0006-3134	Jun	2010	54	2	272	278	10.1007/s10535-010-0047-6
872	Shepherd, M et al.	Geographical and historical determinants of microsatellite variation in *Eucalyptus pilularis*	Canadian Journal of Forest Research-Revue Canadienne De Recherche Forestiere	Article	0045-5067	Jun	2010	40	6	1051	1063	10.1139/X10-049
873	Araki, N et al.	Development of microsatellite markers in cultivated and wild species of sections *Cepa* and *Phyllodolon* in *Allium*	Euphytica	Article	0014-2336	Jun	2010	173	3	321	328	10.1007/s10681-009-0087-1
874	Bagavathiannan, MV et al.	Genetic diversity of feral alfalfa (*Medicago sativa* L.) populations occurring in Manitoba, Canada and comparison with alfalfa cultivars: an analysis using SSR markers and phenotypic traits	Euphytica	Article	0014-2336	Jun	2010	173	3	419	432	10.1007/s10681-010-0156-5
875	Carimi, F et al.	Microsatellite analyses for evaluation of genetic diversity among Sicilian grapevine cultivars	Genetic Resources & Crop Evolution	Article	0925-9864	Jun	2010	57	5	703	719	10.1007/s10722-009-9506-3
876	Weng, YQ et al.	An extended intervarietal microsatellite linkage map of cucumber, *Cucumis sativus* L.	Hortscience	Article	0018-5345	Jun	2010	45	6	882	886	NA
877	Lee, JH et al.	Isolation and characterization of 13 microsatellite loci from Korean *Quercus acuta* (Fagaceae)	Journal of Plant Biology	Article	1226-9239	Jun	2010	53	3	201	204	10.1007/s12374-010-9105-z
878	Ince, AG et al.	Polymorphic microsatellite markers transferable across *Capsicum* species	Plant Molecular Biology Reporter	Article	0735-9640	Jun	2010	28	2	285	291	10.1007/s11105-009-0151-y
879	Bowles, VG et al.	A phylogenetic investigation of *Carthamus* combining sequence and microsatellite data	Plant Systematics & Evolution	Article	0378-2697	Jun	2010	287	1-2	85	97	10.1007/s00606-010-0292-3
880	Chandna, R et al.	Variability in Indian bread wheat (*Triticum aestivum* L.) varieties differing in nitrogen efficiency as assessed by microsatellite markers	Protoplasma	Article	0033-183X	Jun	2010	242	1-4	55	67	10.1007/s00709-010-0122-z
881	Albaladejo, RG et al.	Isolation of microsatellite markers for the common mediterranean shrub *Myrtus communis* (Myrtaceae)	American Journal of Botany	Article	0002-9122	May	2010	97	5	E23	E25	10.3732/ajb.1000060
882	Arroyo, JM et al.	Isolation and characterization of 20 microsatellite loci for laurel species (*Laurus*, Lauraceae)	American Journal of Botany	Article	0002-9122	May	2010	97	5	E26	E30	10.3732/ajb.1000069
883	Kabat, SM et al.	Isolation and characterization of microsatellite loci in the common milkweed, *Asclepias syriaca* (Apocynaceae)	American Journal of Botany	Article	0002-9122	May	2010	97	5	E37	E38	10.3732/ajb.1000064
884	Luettmann, K et al.	Characterization of nuclear microsatellite loci in the neotropical tree *Parkia panurensis* (Fabaceae)	American Journal of Botany	Article	0002-9122	May	2010	97	5	E34	E36	10.3732/ajb.1000096
885	Raabova, J et al.	Development and multiplexing of microsatellite markers in the polyploid perennial herb, *Menyanthes trifoliata* (Menyanthaceae)	American Journal of Botany	Article	0002-9122	May	2010	97	5	E31	E33	10.3732/ajb.1000084
886	Oliveira, EJ et al.	Polymorphic microsatellite marker set for *Carica papaya* L. and its use in molecular-assisted selection	Euphytica	Article	0014-2336	May	2010	173	2	279	287	10.1007/s10681-010-0150-y
887	Zhou, W et al.	Isolation and characterization of 13 microsatellite loci from *Luculia pinceana* (Rubiaceae), a typical distylous species	Hortscience	Article	0018-5345	May	2010	45	5	840	841	NA
888	Wang, XW et al.	Development of microsatellite markers from crape myrtle (Lagerstroemia L.)	Hortscience	Article	0018-5345	May	2010	45	5	842	844	NA
889	Yuan, JH et al.	Hybrid origin of *Paeonia* x *yananensis* revealed by microsatellite markers, chloroplast gene sequences, and morphological characteristics	International Journal of Plant Sciences	Article	1058-5893	May	2010	171	4	409	420	10.1086/651228
890	Castillo, NRF et al.	Microsatellite markers for raspberry and blackberry	Journal of the American Society for Horticultural Science	Article	0003-1062	May	2010	135	3	271	278	NA
891	Moriguchi, Y et al.	Mating patterns in an indoor miniature *Cryptomeria japonica* seed orchard as revealed by microsatellite markers	New Forests	Article	0169-4286	May	2010	39	3	261	273	10.1007/s11056-009-9169-0
892	Van Inghelandt, D et al.	Population structure and genetic diversity in a commercial maize breeding program assessed with SSR and SNP markers	Theoretical & Applied Genetics	Article	0040-5752	May	2010	120	7	1289	1299	10.1007/s00122-009-1256-2
893	Nayak, SN et al.	Integration of novel SSR and gene-based SNP marker loci in the chickpea genetic map and establishment of new anchor points with *Medicago truncatula* genome	Theoretical & Applied Genetics	Article	0040-5752	May	2010	120	7	1415	1441	10.1007/s00122-010-1265-1
894	Barro-Kondombo, C et al.	Genetic structure among sorghum landraces as revealed by morphological variation and microsatellite	Theoretical & Applied Genetics	Article	0040-5752	May	2010	120	8	1511	1523	10.1007/s00122-010-1272-2

	Author	Publication title	Source	Publication type	ISSN	Publication date	Vol	Issue	Pages	DOI
		markers in three agroclimatic regions of Burkina Faso								
895	Neophytou, C et al.	Detecting interspecific and geographic differentiation patterns in two interfertile oak species (*Quercus petraea* (Matt.) Liebl. and *Q. robur* L.) using small sets of microsatellite markers	Forest Ecology & Management	Article	0378-1127	Apr 30 2010	259	10	2026 2035	10.1016/j.foreco.2010.02.013
896	Brzyski, JR	Isolation and characterization of microsatellite markers in the rare clonal plant, *Spiraea virginiana* (Rosaceae)	American Journal of Botany	Article	0002-9122	Apr 2010	97	4	E20 E22	10.3732/ajb.1000008
897	Wu, JC et al.	Isolation and characterization of twenty polymorphic microsatellite loci for *Moringa oleifera* (Moringaceae)	Hortscience	Article	0018-5345	Apr 2010	45	4	690 692	NA
898	Xu, P et al.	Development and polymorphism of *Vigna unguiculata* ssp *unguiculata* microsatellite markers used for phylogenetic analysis in asparagus bean (*Vigna unguiculata* ssp *sesquipedialis* (L.) Verdc.)	Molecular Breeding	Article	1380-3743	Apr 2010	25	4	675 684	10.1007/s11032-009-9364-x
899	Carlsen, T et al.	The evolutionary history of Beringian *Smelowskia* (Brassicaceae) inferred from combined microsatellite and DNA sequence data	Taxon	Article	0040-0262	Apr 2010	59	2	427 438	NA
900	Arakaki, M et al.	Characterization of polymorphic microsatellite loci in *Haageocereus* (Trichocereeae, Cactaceae)	American Journal of Botany	Article	0002-9122	Mar 2010	97	3	E17 E19	10.3732/ajb.1000026
901	Culley, TM; Stewart, JR	Microsatellite primers in *Rhamnus cathartica* (Rhamnaceae) and applicability in related taxa to assess hybridization events	American Journal of Botany	Article	0002-9122	Mar 2010	97	3	E7 E9	10.3732/ajb.0900394
902	Liu, Y et al.	Development of microsatellite markers for the moss *Ptychomitrium gardneri* (Ptychomitriaceae)	American Journal of Botany	Article	0002-9122	Mar 2010	97	3	E14 E16	10.3732/ajb.1000014
903	Simon, VI et al.	New microsatellite loci for *Narcissus papyraceus* (Amarillydaceae) and cross-amplification in other congeneric species	American Journal of Botany	Article	0002-9122	Mar 2010	97	3	E10 E13	10.3732/ajb.1000023
904	Karlin, EF et al.	Microsatellite analysis of *Sphagnum centrale*, *S. henryense,* and *S. palustre* (Sphagnaceae)	Bryologist	Article	0007-2745	Spring 2010	113	1	90 98	10.1639/0007-2745-113.1.90
905	Naghavi, MR et al.	Microsatellite analysis of genetic diversity and population genetic structure of *Aegilops tauschii* Coss. in Northern Iran	Genetic Resources & Crop Evolution	Article	0925-9864	Mar 2010	57	3	423 430	10.1007/s10722-009-9481-8
906	Wang, N et al.	Development and characterization of 15 microsatellite loci for *Rhododendron delavayi* Franch. (Ericaceae)	Hortscience	Article	0018-5345	Mar 2010	45	3	457 459	NA
907	Gurcan, K; Mehlenbacher, SA	Transferability of microsatellite markers in the Betulaceae	Journal of the American Society for Horticultural Science	Article	0003-1062	Mar 2010	135	2	159 173	NA
908	Lopez-Vinyallonga, S	Isolation and characterization of novel microsatellite markers for *Arctium minus* (Compositae)	American Journal of Botany	Article	0002-9122	Feb 2010	97	2	E4 E6	10.3732/ajb.0900376
909	Sharma, S et al.	Phenotypic characterization and nuclear microsatellite analysis reveal genomic changes and rearrangements underlying androgenesis in tetraploid potatoes (*Solanum tuberosum* L.)	Euphytica	Article	0014-2336	Feb 2010	171	3	313 326	10.1007/s10681-009-9983-7
910	Ganeva, G et al.	Genetic diversity assessment of Bulgarian durum wheat (*Triticum durum* Desf.) landraces and modern cultivars using microsatellite markers	Genetic Resources & Crop Evolution	Article	0925-9864	Feb 2010	57	2	273 285	10.1007/s10722-009-9468-5
911	Yan, HF et al.	Isolation and characterization of microsatellite loci for the ornamental plant *Primula obconica* Hance (Primulaceae)	Hortscience	Article	0018-5345	Feb 2010	45	2	314 315	NA
912	Tang, DQ et al.	Development, characterization and utilization of genbank microsatellite markers in *Phyllostachys pubescens* and related species	Molecular Breeding	Article	1380-3743	Feb 2010	25	2	299 311	10.1007/s11032-009-9333-4
913	Chandra, A; Tiwari, KK	Isolation and characterization of microsatellite markers from guineagrass (*Panicum maximum*) for genetic diversity estimate and cross-species amplification	Plant Breeding	Article	0179-9541	Feb 2010	129	1	120 124	10.1111/j.1439-0523.2009.01651.x
914	King, RA et al.	Characterisation and inheritance of nuclear microsatellite loci for use in population studies of the allotetraploid *Salix alba-Salix fragilis* complex	Tree Genetics & Genomes	Article	1614-2942	Feb 2010	6	2	247 258	10.1007/s11295-009-0245-6
915	Liesebach, H et al.	Clonal fingerprinting in the genus *Populus* L. by nuclear microsatellite loci regarding differences between sections, species and hybrids	Tree Genetics & Genomes	Article	1614-2942	Feb 2010	6	2	259 269	10.1007/s11295-009-0246-5
916	Prunier, R; Latimer, A	Microsatellite primers in the white proteas (*Protea* section *Exsertae*, Proteaceae), a rapidly radiating	American Journal of Botany	Article	0002-9122	Jan 2010	97	1	E1 E3	10.3732/ajb.0900326

	Author	Publication title	Source	Publication type	ISSN	Publication date	Vol	Issue	Pages	DOI
		lineage								
917	Huang, Y et al.	Development of 11 polymorphic microsatellite loci from *Primula amthystina* Franchet. (Primulaceae)	Hortscience	Article	0018-5345	Jan 2010	45	1	148 149	NA
918	Liao, SX et al.	Isolation and characterization of polymorphic microsatellite markers in *Calocedrus macrolepis* Kurz (Cupressaceae)	Hortscience	Article	0018-5345	Jan 2010	45	1	169 171	NA
919	Bassil, NV et al.	Microsatellite-based fingerprinting of Western blackberries from plants, IQF berries and puree	International Symposium on Molecular Markers in Horticulturese, Acta Horticulturae	Proceedings Paper	0567-7572BN 978-90-66052-28-4	Jul 2010	859	NA	73 80	NA
920	Bassil, NV et al.	Microsatellite markers distinguish Hawaiian ohelo from other *Vaccinium* L. section *Myrtillus* species	International Symposium on Molecular Markers in Horticulturese, Acta Horticulturae	Proceedings Paper	0567-7572BN 978-90-66052-28-4	Jul 2010	859	NA	81 88	NA
921	Coggeshall, MV; Woeste, KE	Microsatellite and phenological descriptors identify Eastern black walnut cultivars in Missouri, USA	International Symposium on Molecular Markers in Horticulturese, Acta Horticulturae	Proceedings Paper	0567-7572BN 978-90-66052-28-4	Jul 2010	859	NA	93 98	NA
922	Dossett, M et al.	Transferability of *Rubus* microsatellite markers to black raspberry	International Symposium on Molecular Markers in Horticulturese, Acta Horticulturae	Proceedings Paper	0567-7572BN 978-90-66052-28-4	Jul 2010	859	NA	103 109	NA
923	Viji, G et al.	Use of microsatellite markers to characterize genetic diversity of selected accessions of guava (*Psidium guajava*) in the United States	International Symposium on Molecular Markers in Horticulturese, Acta Horticulturae	Proceedings Paper	0567-7572BN 978-90-66052-28-4	Jul 2010	859	NA	169 176	NA
924	Grauke, LJ et al.	Plastid microsatellite markers in *Carya*	International Symposium on Molecular Markers in Horticulturese, Acta Horticulturae	Proceedings Paper	0567-7572BN 978-90-66052-28-4	Jul 2010	859	NA	237 246	NA
925	Nybom, H et al.	Microsatellite and rDNA analysis reveal unique reproduction in dogroses	International Symposium on Molecular Markers in Horticulturese, Acta Horticulturae	Proceedings Paper	0567-7572BN 978-90-66052-28-4	Jul 2010	859	NA	247 253	NA
926	Singode, A; Prasanna, BM	Analysis of genetic diversity in the North Eastern Himalayan maize landraces using microsatellite markers	Journal of Plant Biochemistry & Biotechnology	Article	0971-7811	Jan 2010	19	1	33 41	NA
927	Deng, X et al.	Development and characterization of polymorphic microsatellite markers in *Linum usitatissimum*	Journal of Plant Research	Article	0918-9440	Jan 2010	123	1	119 123	10.1007/s10265-009-0271-3
928	Cota, LC et al.	Preliminary studies on microsatellite marker analysis of resistance to common bunt in several wheat genotypes (*Triticum aestivum* L.)	Notulae Botanicae Horti Agrobotanici Cluj-Napoca	Article	0255-965X	NA 2010	38	2	42 47	NA
929	Ortego, J; Bonal, R	Natural hybridisation between kermes (*Quercus coccifera* L.) and holm oaks (*Q. ilex* L.) revealed by microsatellite markers	Plant Biology	Article	1435-8603	Jan 2010	12	1	234 238	10.1111/j.1438-8677.2009.00244.x
930	Kawase, D et al.	Population genetic structure of *Lilium japonicum* and serpentine plant *L. japonicum* var. *abeanum* by using developed microsatellite markers	Plant Biosystems	Article	1126-3504	NA 2010	144	1	29 37	10.1080/11263500903342721
931	Tang, S et al.	Assessment of genetic diversity and relationships of upland rice accessions from Southwest China using microsatellite markers	Plant Biosystems	Article	1126-3504	NA 2010	144	1	85 92	10.1080/11263500903454237
932	Koch, JL et al.	Use of microsatellite markers in an American beech (*Fagus grandifolia*) population and paternity testing	Silvae Genetica	Article	0037-5349	NA 2010	59	2-3	62 68	NA
933	Omondi, SF et al.	Cross-amplification and characterization of polymorphic microsatellite markers from *Acacia* (*Senegalia) mellifera* and *Acacia brevispica* to *Acacia senegal* (L.) Wild.	Silvae Genetica	Article	0037-5349	NA 2010	59	6	285 288	NA

NA, Not available
